# RNA Sequencing Analyses Reveal the Potential Mechanism of Pulmonary Injury Induced by Gallium Arsenide Particles in Human Bronchial Epithelioid Cells

**DOI:** 10.1038/s41598-020-65518-8

**Published:** 2020-05-26

**Authors:** Yabo Ouyang, Xiaodong Liu, Haibing Li, Shiwei Cui, Huifang Yan, Xingfu Pan

**Affiliations:** 1 Beijing YouAn Hospital, Capital Medical University, Beijing Institute of Hepatology, Beijing, 100069 China; 2 The Beijing Prevention and Treatment of Hospital of Occupational Disease for Chemical Industry, Beijing, 100093 China; 3 National Institute of Occupational Health and Poison Control, Chinese Center for Disease Control and Prevention, Beijing, 100050 China; 4 Beijing Precision Medicine and Transformation Engineering Technology Research Center of Hepatitis and Liver Cancer, Beijing, 100069 China

**Keywords:** Respiratory tract diseases, Risk factors

## Abstract

Extensive use of gallium arsenide (GaAs) has led to increased exposure to humans working in the semiconductor industry. This study employed physicochemical characterization of GaAs obtained from a workplace, cytotoxicity analysis of damage induced by GaAs in 16HBE cells, RNA-seq and related bioinformatic analysis, qRT-PCR verification and survival analysis to comprehensively understand the potential mechanism leading to lung toxicity induced by GaAs. We found that GaAs-induced abnormal gene expression was mainly related to the cellular response to chemical stimuli, the regulation of signalling, cell differentiation and the cell cycle, which are involved in transcriptional misregulation in cancer, the MAPK signalling pathway, the TGF-β signalling pathway and pulmonary disease-related pathways. Ten upregulated genes (FOS, JUN, HSP90AA1, CDKN1A, ESR1, MYC, RAC1, CTNNB1, MAPK8 and FOXO1) and 7 downregulated genes (TP53, AKT1, NFKB1, SMAD3, CDK1, E2F1 and PLK1) related to GaAs-induced pulmonary toxicity were identified. High expression of HSP90AA1, RAC1 and CDKN1A was significantly associated with a lower rate of overall survival in lung cancers. The results of this study indicate that GaAs-associated toxicities affected the misregulation of oncogenes and tumour suppressing genes, activation of the TGF-β/MAPK pathway, and regulation of cell differentiation and the cell cycle. These results help to elucidate the molecular mechanism underlying GaAs-induced pulmonary injury.

## Introduction

Gallium arsenide (GaAs) is a semiconductor material widely used in electronic devices, which are in particularly high demand as electronic components for communication equipment^1–3^. As one of the largest production and processing companies of GaAs, the increasing utilization of GaAs in electronic devices has raised concerns regarding its potential risks to worker health in China. Occupational exposure to GaAs occurs predominantly in the microelectronics industry when workers come into contact with the production of GaAs crystals, ingots and wafers, grinding and sawing operations, device fabrication, and sandblasting and clean-up activities^4–6^.

GaAs is a crystalline intermetallic solid composed of arsenic (As) and gallium (Ga)^7^. Biomonitoring of exposure to GaAs, primarily by measuring As in human tissues or body fluids, has several limitations because occupational exposure limits for arsenic have been established in many countries, and the analytical methods available for measuring GaAs are more sensitive than those for gallium^6^. GaAs is partly dissociated *in vivo* into inorganic As and Ga, but GaAs are found to have lower solubilities than dissolved arsenic^6^. There have been sporadic reports of health effects among workers exposed to this highly toxic arsenic compound^8^. Studies have shown that GaAs are toxic and carcinogenic^9–12^. Because of the ability of GaAs to cause extensive pulmonary damage in the rat, a TLV-TWA of 0.3 μg/m^3^ recommended with an A3-Confirmed Animal Carcinogen with Unknown Relevance to Humans, designation^13^. GaAs particles were observed in the alveolar spaces and in macrophages with significant elevations of lung lipids and proteins with intratracheal instillation of GaAs particulates^14^. Despite the increasing utilization of GaAs in electronic devices, the potential mechanism governing the effect of GaAs on workers through respiratory exposure under workplace conditions has not been elucidated.

An *in vitro* model is an important means to study the adverse effects of particulate pollutants on the respiratory tract and the means of action. To assess the effects of particle pollution on the respiratory tract, it is important to develop systems and methods that allow cultured cells to be repeatedly exposed to particles in the mixture. Human bronchial epithelioid cells (16HBE cells) have been successfully used in lung toxicity tests to evaluate the cytotoxicity and metabolism of PM2.5 and gaseous pollutants^15^.

The objective of this study was to evaluate the cytotoxic effects of GaAs particles on 16HBE cells, to obtain differentially expressed genes induced by GaAs particles, and to reveal potential mechanisms of pulmonary injury after exposure. To better understand the potential molecular mechanisms underlying the pulmonary toxicity of the GaAs particles, 16HBE cells were chosen as the *in vitro* exposure model, and then RNA-seq, related bioinformatic analysis, qRT-PCR verified analysis and survival curve analysis using The Cancer Genome Atlas (TCGA) data were conducted.

## Results

### Cytotoxicity induced by GaAs

**Figure 1:**
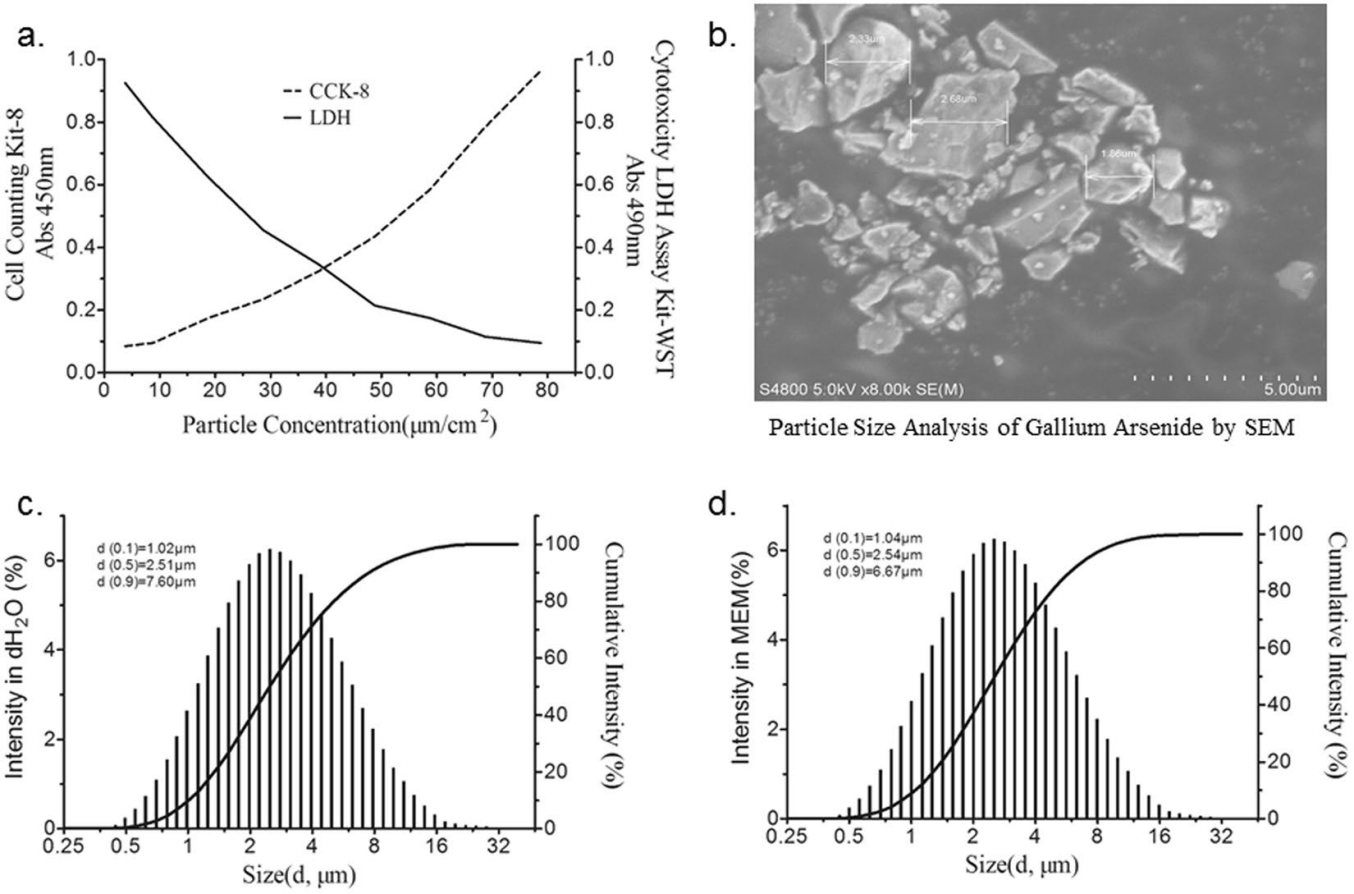
Assessment of cell viability and characterization of GaAs particles. (**a**) Cell viability measured with LDH and CCK-8. (**b**) Scanning electron microscope (SEM) images of the GaAs particles. Scale bar, 0.5 μm. (**c**) The hydrodynamic sizes of GaAs particles in dH2O. (**d**) The hydrodynamic sizes of GaAs particles in MEM culture medium.

To evaluate the possible cytotoxicity of GaAs particles on 16HBE cells, cell viability was measured after exposing the cells to 5.0, 10.0, 20.0, 30.0, 40.0, 50.0, 60.0, 70.0, and 80.0 μg/cm^2^ of GaAs particles for 24 h in 96-well plates. As shown in Fig. 1a, cell viability decreased and cell mortality increased gradually in a dose-dependent manner as the concentration increased. As the dose increased to 20.0 μg/cm^2^, the cell viability lost nearly 15%, as measured with LDH, and cell mortality increased nearly 20%, as measured with CCK-8, which was significant compared with that in the control group. Therefore, 20.0 μg/cm^2^ was chosen as the experimental dose for the following analysis.

### Physicochemical characterization of GaAs

The characteristics of GaAs particles are given in Fig. 1. GaAs particles appeared as agglomerates of different sizes, which were mainly composed of regular or irregularly shaped ultrafine particles and fine particles. The morphology of the particles obtained from SEM is mostly irregular, as shown in Fig. 1b. The hydrodynamic diameter of GaAs particles measured in dH_2_O and in MEM culture medium are shown in Fig. 1c,d. The hydrodynamic size (median) of GaAs particles is 2.51 μm or 2.54 μm when GaAs particles are suspended in dH_2_O or MEM, respectively.

The average concentrations of As and Ga in MEM culture medium after exposure for 24 h were 1.0 and 0.8 μg/mL. In the negative control, the concentrations were less than 0.02 μg/mL. Other heavy metals, including Be, Al, Ti, V, Cr, Mn, Co, Ni, Cu, Se, Sr, Zr, Mo, Pd, Ag, Cd, Cs, and Pb, were detected in MEM culture medium after exposure for 24 h. Only Mo and Pd were significantly different from the NC group, which was significantly higher than the control group. The average concentrations of Mo and Pd were 18.5 and 8.0 μg/L, respectively.

### Differential gene expression induced by GaAs

**Figure 2:**
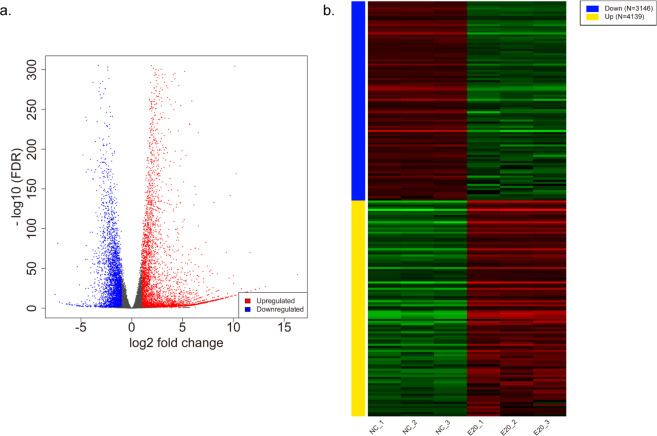
Differentially expressed genes (DEGs) induced by GaAs in 16HBE cells. (**a**) Volcano plot showing that 3146 genes were downregulated and 4139 genes were upregulated between the GaAs and NC groups. DEGs with log2FoldChange (log2FC)>1 are labelled in red; DEMs with log2FoldChange (log2FC) <−1 are labelled in blue (P < 0.05). (**b**) The DEG expression profiles of mRNAs are presented as a heatmap.

We performed RNA-seq to investigate the possible gene expression change in 16HBE cells induced by GaAs. After quality control of raw sequencing data, the clean reads were mapped to the human reference genome (hg19), and the median total mapping rates were 94.63% in the NC group (93.9%–94.79%) and 87.06% in the E20 group (86.85%–87.72%). The mRNA expression levels and transcripts were estimated with FPKMs. A total of 7285 mRNA transcripts, including 4139 upregulated and 3146 downregulated transcripts (p < 0.05), were differentially expressed in the EC20 group relative to the NC group (Fig. 2a). These differentially expressed mRNAs were used for subsequent analysis. Cluster analysis of differentially expressed mRNAs was conducted with heat maps (Fig. 2b). The top 20 differentially expressed upregulated and downregulated genes between the NC group and GaAs-treated group are listed in Table 1.

**Table 1:** The top 20 differentially expressed genes between GaAs and NC groups in 16HBE.

	Gene Symbol	Mean of readcount (GaAs)	Mean of readcount (NC)	log2FoldChange	padj
Up regulated genes	CRYAB	13354.47715	0	16.71152296	1.34E-44
	HSPA6	570082.8219	15.59458786	15.1533678	0
	RFPL4A	2874.493451	0.190242569	13.53369186	1.39E-29
	CT45A1	992.143666	0	12.96069412	4.14E-27
	SERPINA7	758.5311161	0	12.57396271	1.72E-25
	CCL26	1455.726687	0.228898208	12.55200595	1.52E-25
	NEFM	554.9236214	0	12.1230176	1.04E-23
	RFPL4AL1	433.9097466	0	11.7672136	2.42E-22
	KRTAP2-3	428.0256979	0	11.74782879	2.78E-22
	PRR9	781.3895624	0.190242569	11.65379393	5.04E-22
	FMR1NB	392.3592048	0	11.62220187	8.46E-22
	CD300LB	358.4648122	0	11.49310827	3.14E-21
	FGF19	336.4592526	0	11.40062334	5.94E-21
	ARC	4688.385657	1.676563105	11.35752989	4.56E-106
	MLC1	324.872728	0	11.35041552	8.96E-21
	ZNF556	314.8386264	0	11.30504573	1.32E-20
	TEX19	1042.109777	0.453565577	11.18744001	8.36E-27
	LBH	480.1538676	0.263323009	10.95168561	1.55E-19
	PRSS55	198.501012	0	10.63995065	3.96E-18
	ITK	190.6265953	0	10.5810697	5.95E-18
Down regulated genes	PLEKHS1	0.914321697	209.7064732	−7.761268855	4.09E-13
	PCSK9	5.228998183	932.7962655	−7.454393368	4.88E-61
	GBP4	0.457160848	55.93539395	−6.748976858	1.85E-07
	SLC16A7	0	24.04444067	−6.491580583	3.12E-06
	PRR15L	0	18.22963028	−6.092787428	2.85E-05
	KCNIP2	0	17.03020545	−5.990095124	5.25E-05
	VASH1	0.517292735	30.63114054	−5.878827459	1.56E-05
	UGT1A6	0.457160848	29.38882924	−5.816345507	2.16E-05
	CHST4	0	14.2640851	−5.738229644	0.000142003
	TNFSF10	0.937671191	51.45654047	−5.735989035	5.74E-07
	JMJD7	0	14.0599548	−5.714919551	0.000168574
	LCN12	0	12.61771595	−5.559550172	0.000360681
	FGD3	7.852244248	353.8214632	−5.512552741	9.16E-42
	DEFB131B	0	11.8362086	−5.468792097	0.000480387
	ALDH3B2	4.508505043	186.9671612	−5.420111415	1.07E-23
	GOLGA8O	0	11.38144781	−5.409920859	0.000662611
	FAM198B	0.517292735	20.6261176	−5.308205572	0.000235478
	EDDM13	0.457160848	19.45743674	−5.225385183	0.00028589
	MXRA5	0.517292735	17.57855567	−5.080106293	0.000506725
	TMX2-CTNND1	0	8.823688589	−5.052477188	0.003958624

### GO analysis of differential gene expression induced by GaAs

**Figure 3:**
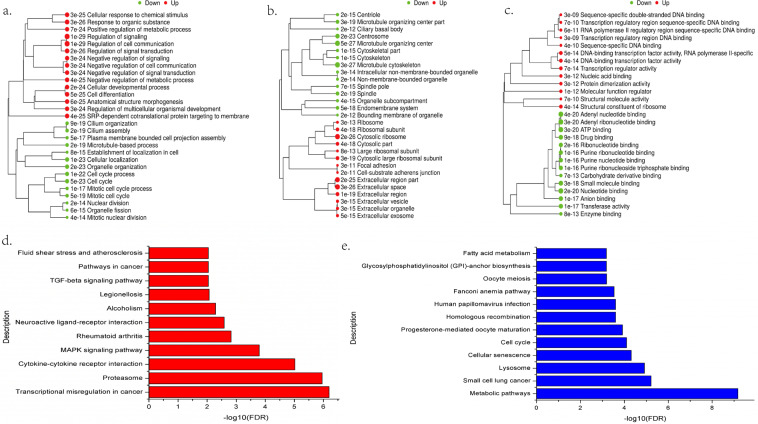
Significantly changed up- and downregulated GO terms and pathways of differentially expressed genes (DEGs) induced by GaAs in 16HBE cells. (**a**) Significant up- and downregulation GO terms during BP; (**b**) Significant up- and downregulation GO terms during CC; (**c**) Significant up- and downregulation GO terms during MF; (**d**) Significantly changed pathways of differentially expressed upregulated genes based on the KEGG database. −log10(FDR), negative logarithm of the adjusted *P* value. FDR < 0.05 was identified as a significantly changed pathway. (**e**) Significantly changed pathways of differentially expressed downregulated genes based on the KEGG database.

First, GO and KEGG analyses were performed on 7285 significantly dysregulated mRNAs in GaAs vs NC. We derived 85 highly enriched GO terms (adj. *P* val <0.05) and 27 significantly enriched pathways (adj. *P* val <0.05). The top 10 significant GO terms during biological process (BP), cellular component (CC) and molecular function (MF) induced by GaAs are displayed in Fig. 3a–c. Several GO terms, such as cellular response to chemical stimulus, regulation of signalling, cell differentiation, cell cycle, focal adhesion, transcription regulator activity and small molecule binding, were closely related to GaAs exposure. As shown in Fig. 3d, the significantly upregulated pathways induced by GaAs included transcriptional misregulation in cancer, cytokine-cytokine receptor interaction, MAPK signalling pathway, TGF-β signalling pathway and pathways in cancer. Significantly downregulated pathways, such as metabolic pathways, small cell lung cancer, cell cycle, and fatty acid metabolism, were detected (Fig. 3e). The summaries of genes involved in the significant up- and downregulation pathways are listed in Supplementary Table S2.

### DO analysis of differential gene expression induced by GaAs

**Figure 4:**
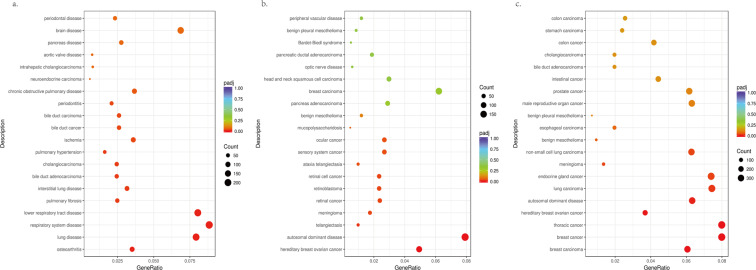
Disease ontology (DO) enrichment analysis of differentially expressed genes (DEGs) induced by GaAs in 16HBE cells. The x-axis indicates the number of enriched genes in the given DO category mapping to the size of the dots. The colour-coding indicates the adjusted p value. (**a**) Significantly upregulated Dos; (**b**) Significantly downregulated Dos; (**c**) Significantly dysregulated regulatory Dos.

DO (Disease Ontology) is a database describing human gene function and disease that can be used to consider the interactions and functions of differentially expressed genes with disease. The top 20 enrichment terms related to human disease were recognized based on the DO database. The P < 0.05 gene list was also analysed for DO annotation, enabling us to study gene-disease relationships. Significantly upregulated genes induced by GaAs in 16HBE using DO analysis may be related to lung disease, respiratory system disease, lower respiratory tract disease, pulmonary fibrosis, interstitial lung disease and chronic obstructive pulmonary disease (Fig. 4a). For example, hereditary breast ovarian cancer, retinal cancer and ocular cancer were relevant to the downregulation of DO-related diseases (Fig. 4b). Taken together, these results indicate that dysregulated DO of differentially expressed genes induced by GaAs in 16HBE may be related to lung carcinoma, non-small-cell lung carcinoma and thoracic cancer (Fig. 4c).

### Protein-protein interaction (PPI) analysis revealed the key genes triggered by GaAs

**Figure 5:**
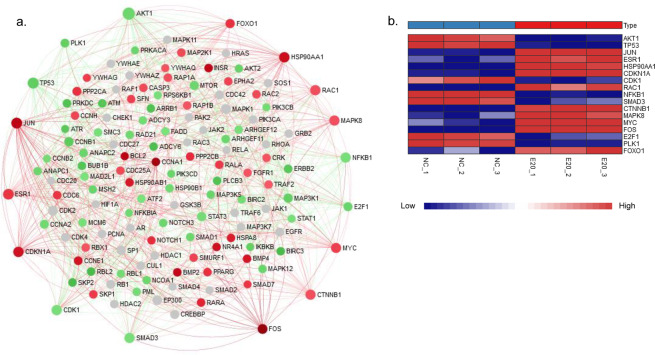
Protein-protein interaction (PPI) network of differentially expressed genes (DEGs) induced by GaAs in 16HBE cells. The PPI network was drawn using the NetworkAnalyst platform based on the STRING interactome. (**a**) The red circles represent upregulated genes, the green circles represent downregulated genes, and the grey circles indicate no DEGs. The top degree (>20) involved in the interaction network was screened; (**b**) The heatmap shows the expression level of the 17 key genes in the RNA-seq data.

PPI analysis of the STRING interactome was used to screen the key genes involved in GaAs-induced toxicity in 16HBE cells. According to the significant genes from the significant pathway and DO analysis, we next focused on the DEGs in the cell cycle pathway, pathways in cancer, transcriptional misregulation in cancer, small-cell lung cancer, the MAPK signalling pathway, the TGF-β signalling pathway and non-small-cell lung cancer, and the corresponding significant genes were mapped to the corresponding molecular interaction database (Fig. 5a). Seventeen key genes with the top degree (>20) involved in the above 6 pathway interaction network were screened (Table 2), which may play an important role in GaAs-induced pulmonary toxicity in 16HBE cells. These 17 key genes consisted of 10 upregulated genes (FOS, JUN, HSP90AA1, CDKN1A, ESR1, MYC, RAC1, CTNNB1, MAPK8 and FOXO1) and 7 downregulated genes (TP53, AKT1, NFKB1, SMAD3, CDK1, E2F1 and PLK1) (Fig. 5b).

**Table 2:** The top genes ranked by degree over 20 in PPI analysis.

Gene symbol	Description	KEGG Pathways	gene_chr	Degree	Betweenness	log2FoldChange (GaAs vs.NC)
AKT1	v-akt murine thymoma viral oncogene homolog 1, protein_coding	Small cell lung cancer	14	57	972.3	−1.961368001
TP53	tumor protein p53, protein_coding	Small cell lung cancer, Cell cycle	17	41	470.66	−2.592326592
JUN	jun proto-oncogene, protein_coding	Pathways in cancer, MAPK signaling pathway	1	39	282.42	4.837850274
ESR1	estrogen receptor 1, protein_coding	Pathways in cancer	6	37	337.46	1.787520803
HSP90AA1	heat shock protein 90 kDa alpha (cytosolic), class A member 1, protein_coding	Pathways in cancer	14	34	417.35	3.926057435
CDKN1A	cyclin-dependent kinase inhibitor 1A (p21, Cip1), protein_coding	Pathways in cancer, Transcriptional misregulation in cancer	6	34	301.04	3.632760311
CDK1	cyclin-dependent kinase 1, protein_coding	Cell cycle	10	33	346.53	−1.423420996
RAC1	ras-related C3 botulinum toxin substrate 1 (rho family, small GTP binding protein Rac1), protein_coding	Pathways in cancer, MAPK signaling pathway	7	33	297.85	1.045045893
NFKB1	nuclear factor of kappa light polypeptide gene enhancer in B-cells 1, protein_coding	Small cell lung cancer	4	32	372.86	−1.465472937
SMAD3	SMAD family member 3, protein_coding	Cell cycle	15	32	242.91	−1.428252719
CTNNB1	catenin (cadherin-associated protein), beta 1, 88 kDa, protein_coding	Pathways in cancer	3	32	264.91	1.011091149
MAPK8	mitogen-activated protein kinase 8, protein_coding	Pathways in cancer, MAPK signaling pathway	10	32	260.71	1.011586254
MYC	v-myc myelocytomatosis viral oncogene homolog (avian), protein_coding	Pathways in cancer, MAPK signaling pathway, Transcriptional misregulation in cancer, TGF-beta signaling pathway	8	31	190.63	1.439621447
FOS	FBJ murine osteosarcoma viral oncogene homolog, protein_coding	Pathways in cancer, MAPK signaling pathway	14	28	98.25	7.328134023
E2F1	E2F transcription factor 1, protein_coding	Small cell lung cancer, Cell cycle	20	21	72.8	−2.011988593
PLK1	polo-like kinase 1, protein_coding	Cell cycle	16	20	322.92	−2.341394476
FOXO1	forkhead box O1, protein_coding	Pathways in cancer, Transcriptional misregulation in cancer	13	23	93.93	1.906896342

### qRT-PCR verification and survival curve analysis

**Figure 6:**
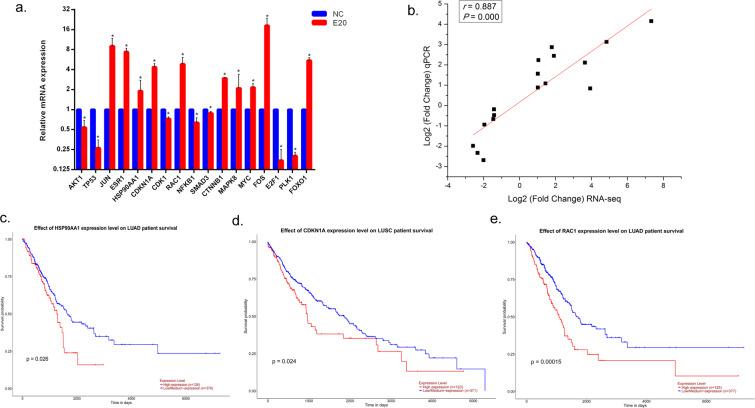
qRT-PCR verification and survival curve analysis. (**a**) Gene expression by qRT-PCR analysis. The fold changes were calculated by using the 2^−ΔΔCt^ method comparing the GaAs-treated group to the NC group. Data are expressed as the means ± S.D. from 3 biological repeats and 3 technical repeats. *P < 0.05 compared to control groups by Mann-Whitney test. (**b**) The potential correlation between the expression of dysregulated genes from RNA-seq and qRT-PCR verification was analysed using Spearman’s rank test. A P value less than 0.05 was considered statistically significant. (**c**) TCGA data from UALCAN demonstrated that high expression of HSP90AA1 predicted a significantly poor prognosis of lung adenocarcinoma (LUAD) patients (P < 0.05); (**d**) TCGA data from UALCAN demonstrated that high expression of RAC1 predicted a significantly poor prognosis of lung squamous cell carcinoma (LUSC) patients (P < 0.05); (**e**) TCGA data from UALCAN demonstrated that high expression of CDKN1A predicted a significantly poor prognosis of LUAD patients (P < 0.05).

To verify the RNA-seq analysis results, the expression of the above 17 significantly dysregulated genes selected from PPIs was verified using qRT-PCR (Fig. 6a). These selected genes were related to various pathways (Table 2), such as small-cell lung cancer (AKT1, TP53, NFKB1 and E2F1),

MAPK signalling pathway (JUN, RAC1, MAPK8, MYC and FOS) and transcriptional misregulation in cancer (CDKN1A, MYC and FOXO1). The potential correlation between the expression of the above 17 significantly dysregulated genes from RNA-seq and qRT-PCR verification was analysed with the Spearman rank order correlation test. We found that the gene expression results of qPCR were positively correlated with the corresponding results of RNA-seq (Fig. 6b; r = 0.887, P = 0.000). The results suggest that good consistency was obtained between RNA-seq analysis and qRT-PCR verification.

Then, we performed 10 upregulated genes for survival curve analysis using TCGA data by UALCAN. Further survival analyses on these key genes were employed to evaluate their effects on the survival of lung cancer. Figure 6c–e shows that HSP90AA1, RAC1 and CDKN1A expression levels were clearly related to the prognosis of lung adenocarcinoma (LUAD) or lung squamous cell carcinoma (LUSC) patients, which indicated that high expression of the three genes was significantly associated with a lower rate of overall survival.

## Discussion

Workers in the semiconductor manufacturing industry are potentially exposed to inhalable GaAs dusts. Because GaAs has a lower solubility than any other arsenic compound and is associated with lung toxicity, even linked to lung neoplasm risk, exploring the toxicogenomic effects of GaAs on human bronchial epithelial cells is critical to understanding the mechanisms of these adverse health effects^7,16^. In this study, we first examined the physicochemical characterization of GaAs from a workplace and cytotoxicity induced by GaAs and then conducted RNA-seq, related bioinformatic analysis, qRT-PCR verification and survival analysis to comprehensively understand potential genes and pathways that lead to lung toxicity induced by GaAs. Our aim was to elucidate the role of GaAs in 16HBE cells and to provide a mechanistic explanation for GaAs exposure increasing pulmonary injury risk in humans. This research was the first attempt to perform transcriptome sequencing analysis to fully understand GaAs-induced toxicity in 16HBE cells.

GaAs could release gallium and arsenic moieties *in vitro* though a relatively lower dissolution rate^17^. In our study, the dissolution rate of GaAs particles was approximately 2.3% after 24 h of exposure to 16HBE cells in the cell supernatant. In addition to dissolution of the above two metals, the toxic effect depends on particle size, exposure duration and exposure route^18^. We collected the GaAs from a workplace that reflects the real environmental exposure, in which the hydrodynamic median sizes of GaAs particles are 2.51 μm or 2.54 μm when suspended in dH_2_O or MEM, respectively. It was demonstrated that micron-sized GaAs particles can result in hazardous pulmonary effects in animal studies, which indicates that a smaller fraction of GaAs is a relatively more severe pneumotoxicant^19,20^. It was reported that a strong inflammatory response induced by solid GaAs in the lungs disappeared when the gallium and arsenic oxides dissolved, as shown in animal models, in which the unchanged GaAs particles were cleared from the lung^20^. Then, the toxic effects of micron-sized GaAs particles exposed to 16HBE were evaluated.

The lung is one of the target organs for the toxic effects of GaAs, as this organ has higher absorption after intratracheal administration than is observed after oral dosing^5^. In inhalation studies, lung retention of inhaled GaAs dust has been shown to be influenced by toxic effects from GaAs itself^19^. GaAs is a compound of gallium and arsenic that is responsible for the toxicity properties of these two metals. Gallium is considered mildly toxic because most of this metal is excreted through urine or faeces, which does not contribute significantly to the lung toxicity of GaAs^21^. Arsenic exposure has been shown to induce human tumourigenesis, and the lung is one of the main targets^22^. Toxicities of arsenic-induced carcinogenicity, including generation of oxidative stress, altered cell proliferation, changes in DNA methylation and co-carcinogenesis, though the exact molecular mechanism governing this toxicity is not well understood^18,23,24^. The toxicity of GaAs in pulmonary tissue includes inflammation, lung weight increase, fibrosis, seropurulent pneumonia and pneumocyte hyperplasia^18,20,25^.

Our results indicated that GaAs induced abnormal gene expression mainly related to cellular response to chemical stimulus, regulation of signalling, cell differentiation, the cell cycle and regulation of signalling. GaAs exposure in 16HBE cells induced the upregulation of cell differentiation-related genes and the downregulation of cell cycle-related genes, which indicated that GaAs is an inducer of cell differentiation. In 16HBE cells after 24 h of GaAs exposure, we also observed morphological changes, such as irregular shapes, smaller volumes and abnormal nuclei. GaAs-related dysregulated genes are involved in signal transduction pathways, such as transcriptional misregulation in cancer, cytokine-cytokine receptor interactions, the MAPK signalling pathway and the TGF-β signalling pathway. It has been summarized that arsenic-induced lung tumours may, through disruption of the PI3K/AKT signalling pathway, activate the EGFR signalling pathway and affect the NRF2 signalling pathway to play a role in carcinogenesis^22^. The TGF-β and MAPK pathways play critical roles in cell development and cell cycle regulation, even in tumour formation and metastasis. It has been shown that activation of MAPK and TGF-β could be induced by ROS accumulation, and the MAPK pathway and TGF-β pathways were closely related to lung fibrosis^26,27^. TGF-β can activate MAPK signalling, and inhibition of the TGF-β/MAPK pathway could protect against lung fibrosis^28,29^, which may explain the GaAs toxicity in pulmonary tissue. Then, a view of the global gene-disease relationships of GaAs exposure in 16HBE was obtained by DO analysis. Lung disease and respiratory system disease were found in significantly upregulated genes induced by GaAs in 16HBE. All dysregulated genes in the significant DO term were clustered into tumour-related diseases, including lung carcinoma and non-small-cell lung carcinoma. These results indicated that pulmonary disease-related pathways were affected when GaAs exposure occurred in 16HBE.

Then, we screened the 17 key genes with the top degree (>20) involved in significantly related pathways of GaAs-induced pulmonary toxicity in 16HBE cells, whose gene expression levels were verified by qPCR. Among these key genes, several oncogenes (FOS, JUN, MYC) and tumour suppressor genes (TP53) were associated with lung cancer development. ESR1/2 might directly or indirectly regulate oncogenic pathways in non-small-cell lung cancer, and FOXO1 expression is a favourable prognostic factor in non-small-cell lung cancer^30,31^. CTNNB1 has been found to be genetically mutated in various human cancers, including lung adenocarcinoma, whose gene expression was increased in lung tissue with pulmonary fibrosis^32^. MAPK8 was found to be potentially related to lung cancer^33^. Finally, the survival curve analysis of 10 upregulated genes using TCGA data indicated that high expression of HSP90AA1, RAC1 and CDKN1A induced by GaAs was clearly related to the lower survival probability in LUAD or LUSC patients. HSP90AA1 was found to be directly associated with lung cancer^34^. The overexpression of Rac1 was linked to aggressive growth and other malignant characteristics of tumours, and a high level of Rac1 could predict a poor prognosis in different types of cancer^35^. CDKN1A expression is upregulated in long-term oxidative stress-induced experimental bronchopulmonary dysplasia and in a hyperoxia-induced lung injury rat model^36,37^. The results of this study indicate that GaAs-associated toxicities affect the misregulation of oncogenes and tumour suppressing genes, activation of the TGF-β/MAPK pathway, and regulation of cell differentiation and the cell cycle.

## Methods

### Particle sample collection

GaAs particles, obtained from a manufacturer of gallium arsenide crystals in Beijing, were collected using an IOM personal sampler (SKC USA) equipped with a cylindrical body, 37 mm cassette, and PVC filter with enough time. Then, the samples were rinsed with distilled water, and the weight change was recorded to calculate the collection quality of the particles. We chose the wafer manufacturing process as the sampling site in which workers are only exposed to GaAs particles.

### Cell culture

The human bronchial epithelial cell line (16HBE14O-, abbreviated as 16HBE) was a gift from Dr D.C. Gruenert (University of California, San Francisco, USA). The cells were maintained in MEM culture medium (Gibco, USA) supplemented with 10% foetal bovine serum (Gibco, USA), 100 U/mL penicillin and 100 μg/mL streptomycin and cultured at 37 °C in a 5% CO_2_ humidified environment.

### Assessment of cell viability

To evaluate the cytotoxicity and cell viability induced by GaAs particles, the Cell Counting Kit-8 (CCK-8) assay and Cytotoxicity LDH Assay Kit-WST (LDH)^38^ assay were employed to assess mitochondrial dehydrogenase activity and loss of cell integrity, respectively. Three biological replicates were performed. Cytotoxicity EC_20_ (effective concentration resulting in 20%) of 16HBE cells exposed to GaAs particles was determined. The cells were exposed to GaAs particles at 5.0, 10.0, 20.0, 30.0, 40.0, 50.0, 60.0, 70.0, and 80.0 μg/cm^2^ for 24 h in a 96-well orifice plate. The cells were incubated for an additional 1 h at 37 °C. The detailed procedures of the CCK-8 assay and LDH assay are available as the product operation instructions. Medium-only treated cells were used as negative control exposures. Optical density at 450 nm and 490 nm was detected by microplate reader (Themo Multiskan MK3, USA), respectively. These data were used to determine appropriate GaAs particle exposure concentrations for subsequent assays.

### Physicochemical characterization of GaAs particles

Scanning electron microscopy (SEM HITACHI S4800) was used to examine the particle morphology. Characterization of GaAs particles was performed at a concentration identical to the cytotoxicity EC_20_. The hydrodynamic diameter of GaAs particles was determined by dynamic light scattering (DLS) using a MASTERSIZER 2000 (Malvern Instruments, Malvern, UK). GaAs particles were suspended in dH_2_O and in MEM at the concentration of cytotoxicity EC_20_ and sonicated prior to measurement.

Dissolution of GaAs particles was assessed in full MEM. GaAs particles were incubated at the concentration of Cytotoxicity EC_20_ for 24 h at 37 °C. Three biological replicates were performed. After incubation, the samples were centrifuged at 25,000 × g for 30 min, and the supernatant was used for Ga and As determination by inductively coupled plasma mass spectrometry (ICP-MS, Thermo).

### GaAs particle exposure

For experiments, the cells were seeded in culture plates at a density of 1 × 10^5^ cells/mL, allowed to attach for 24 h, and treated with GaAs particles suspended in MEM culture medium of certain concentrations for another 24 h. A suspension of GaAs particles was dispersed by a sonicator (Bioruptor UDC-200, Belgium), diluted to EC_20_ concentrations, and immediately added to 16HBE cells. In this study, 6-well culture plates were used, the bottom area of which was 9.6 cm^2^. To ensure that each unit area of cells was exposed to the same amount of GaAs particles, the exposure volume of the GaAs particle suspension was calculated according to the bottom area of the cell culture plate. Cells maintained in MEM culture medium without GaAs particles were used as the control group. Three biological replicates were performed.

### Total RNA extraction and RNA-seq

Total RNA was extracted from the 16HBE cells exposed to EC20 of GaAs particles and negative control (NC) using TRIzol reagent (Invitrogen, San Diego, CA, USA) according to the manufacturer’s instructions. Both groups were conducted in triplicate. A total amount of 3 μg RNA per sample was used as input material for the RNA sample preparations. Sequencing libraries were generated using the NEBNext® UltraTM RNA Library Prep Kit for Illumina® (NEB, USA), and index codes were added to attribute sequences to each sample. The clustering of the index-coded samples was performed on a cBot Cluster Generation System using TruSeq PE Cluster Kit v3-cBot-HS (Illumina). After cluster generation, the library preparations were sequenced on an Illumina HiSeq platform to generate 150 bp paired-end reads (Novogene, Beijing).

### Bioinformatic analysis

The raw sequence files generated from 6 files (bam) in this study has been deposited to NCBI’s Sequence Read Archive (SRA) database with the accession number PRJNA623863. Clean data (clean reads) were obtained by removing reads containing adapters, reads containing poly-N and low-quality reads from raw data using in-house Perl scripts (Novogene, Beijing). FPKM (fragments per kilobase of exon per million fragments) of each gene was calculated based on the length of the gene and read count mapped to this gene. Differential expression analysis of two groups (three biological replicates per group) was performed using the DESeq. 2 R package (1.16.1). The resulting P-values were adjusted using Benjamini and Hochberg’s approach for controlling the false discovery rate. Genes with an adjusted P-value <0.05 found by DESeq. 2 were assigned as differentially expressed.

Gene Ontology (GO) enrichment and Kyoto Encyclopedia of Genes and Genomes (KEGG) pathway analysis of differentially expressed genes was implemented using the clusterProfiler R package and iDEP program^39^, in which gene length bias was corrected. Identified GO terms and KEGG pathways with a corrected p < 0.05 were considered significantly enriched^40^. Disease ontology (DO) annotates human gene-disease relationships, which is important annotation in translating molecular findings from high-throughput data to clinical relevance^41^. DO disease terms and semantic associations were obtained through the DOSE R package, which provided DO terms with a corrected p < 0.05 were considered significantly enriched. Protein-protein interaction (PPI) analysis of differentially expressed genes was based on the STRING database, and the images were examined using the NetworkAnalyst platform^42^.

### Quantitative real-time PCR (qRT-PCR) analysis

The RNeasy Mini Kit (Qiagen, Hilden, Germany) was used to isolate total RNA from 16HBE cells exposed to EC20 of GaAs particles and NC, as described above for the GaAs particle exposure method. Reverse transcription to synthesize first-strand cDNA was conducted using the SuperScript II First-stand Synthesis System for RT-PCR (Invitrogen, USA). The gene expression levels were assessed with the use of TaqMan qRT-PCR assays to validate the GaAs particle-related key genes acquired from RNA-seq. All TaqMan qRT-PCR reactions were carried out using TaqMan® Fast Advanced Master Mix (Applied Biosystems, USA) on a ViiA 7 Real-Time PCR system (Applied Biosystems, USA) in 384-well plates. The relative mRNA expression levels of the target genes were normalized to GAPDH (housekeeping gene), and the fold change was calculated by using the 2^−ΔΔCt^ method. The primers and reaction conditions are listed in Supplementary Table S1.

### Survival analysis

The survival analysis of key GaAs particle exposure-related genes was performed on UALCAN (http://ualcan.path.uab.edu/) to conduct the Kaplan–Meier estimator^43^. The survival curves of samples with high gene expression and low/medium gene expression were compared by the log rank test. P values <0.05 were considered to be significant.

### Statistical analysis

Statistical analysis was performed using SPSS 17.0 software (SPSS, Chicago, IL, USA). The Mann-Whitney test was used to compare the qPCR results between groups. The potential correlation between the expression of dysregulated genes from RNA-seq and qRT-PCR verification was analysed using Spearman’s rank test. P values less than 0.05 were considered to be significant.

## Supplementary information

Supplementary table.
