# Supplementary material for: RNA Sequencing Analyses Reveal the Potential Mechanism of Pulmonary Injury Induced by Gallium Arsenide Particles in Human Bronchial Epithelioid Cells
Source: Sci Rep. 2020 May 26;10:8685. doi: 10.1038/s41598-020-65518-8 (PMC7250905; doi:10.1038/s41598-020-65518-8)
Supplement: Supplementary file 1 — Supplementary table. [file 41598_2020_65518_MOESM1_ESM.docx]

**Supplementary information**

**Title:** RNA Sequencing Analyses Reveal the Potential Mechanism of Pulmonary Injury Induced by Gallium Arsenide Particles in Human Bronchial Epithelioid Cells

**Authors’name:** Yabo Ouyang^1,4^, Xiaodong Liu^2^, Haibing Li^3^, Shiwei Cui^3^, Huifang Yan^3,*^, Xingfu Pan^2,*^

**Authors' information:**

^1^Beijing YouAn Hospital, Capital Medical University, Beijing Institute of Hepatology, Beijing, 100069, China.

^2^The Beijing Prevention and Treatment of Hospital of Occupational Disease for Chemical Industry, Beijing, 100093, China.

^3^National Institute of Occupational Health and Poison Control, Chinese Center for Disease Control and Prevention, Beijing, 100050, China.

^4^Beijing Precision Medicine and Transformation Engineering Technology Research Center of Hepatitis and Liver Cancer, 100069, China

***Correspondence:** X.P. (email: pan_xingfu@hotmail.com); H.Y. (email: yan_huifang@hotmail.com)

**Supplementary table**

1. Quantitative real-time PCR (qRT-PCR) analysis

The Taqman qPCR program on an ABI ViiA 7 Real-Time PCR system in 384-well plates was as follows: 2min at 55°C and 2min at 95°C, followed by 50 cycles of 15 s at 95°C, 30 s at 56°C and 50 s at 72°C, then 4°C holding.

Table S1 List of primers for qRT-PCR

| **Gene** | **Forward** | **Reverse** | **Probe** |
| --- | --- | --- | --- |
| AKT1 | GTGGGTGAGGTGGCTATGAC | ACGCCGCTGCTATTACAAGT | ACGTGGACATGGCCACCATTGCCGA |
| TP53 | CCTGAGGTTGGCTCTGACTG | CACGCACCTCAAAGCTGTTC | CGGCATGAACCGGAGGCCCATCCTC |
| JUN | CGTGAAGTGACGGACTGTTC | GTAGCCATAAGGTCCGCTCT | ACGCCTCGTTCCTCCCGTCCG |
| ESR1 | GACAGGGAGCTGGTTCACAT | AGGATCTCTAGCCAGGCACA | CCATGATCAGGTCCACCTTCTAGAA |
| HSP90AA1 | CCAGAGTGCTGAATACCCGC | TTAACAGGTGCCCTGCTTCTC | CTGAAGCCTCCTCGCCGCCGTTTCT |
| CDKN1A | CTTTGTCACCGAGACACCAC | CAGGTCCACATGGTCTTCCT | ACTCATCCCGGCCTCGCCGG |
| CDK1 | TGTGGCCAGAAGTGGAATCT | TGCCAGAAATTCGTTTGGCT | TGGAAACCAGGAAGCCTAGCATCCCA |
| RAC1 | CCGATTGCCGATGTGTTCTT | AGGATGATGGGAGTGTTGGG | CCTGAGGTGCGGCACCACTGT |
| NFKB1 | GCACCCTGACCTTGCCTATT | GGTCCATCTCCTTGGTCTGC | CAAGCAGAAGGTGGAGGGGACCGGC |
| SMAD3 | CAGCCGGTTTGGATTACAGG | GAGTCAAAGTCCCTGCTCCT | TGGCCACCTTCCTGGCAGGC |
| CTNNB1 | CTTACACCCACCATCCCACT | TGCACGAACAAGCAACTGAA | TGGCACCCTGCTCACGCAAAGGT |
| MAPK8 | GTTCCTGACGTGCACTCTTC | GGATGCTTCTTTGCACACCA | AGTCTGGCATGAACACACTGTGCC |
| MYC | CGGAAGGACTATCCTGCTGC | ACGTTGTGTGTTCGCCTCTT | AAATGCACCAGCCCCAGGTCCTCGG |
| FOS | CAGACTACGAGGCGTCATCC | CGTGGGAATGAAGTTGGCAC | CTTCTGCACGGACCTGGCCGTCTCC |
| E2F1 | CTACGTGACGTGTCAGGACC | AAACATCGATCGGGCCTTGT | CCTGAGACCCAGCTCCAAGCCGTGG |
| PLK1 | GCAGCGTGCAGATCAACTTC | GGAGACTCAGGCGGTATGTG | TTGTGCCCACTGATGGCAGCCGTGA |
| FOXO1 | GTGTCAGGCTGAGGGTTAGT | CTGCCAAGTCTGACGAAAGG | TGGACTGCTTCTCTCAGTTCCTGCTGT |

Table S2 The genes involved in the significantly up and down-regulated pathways.

| Direction | adj.Pval | nGenes | Pathways | Genes |
| --- | --- | --- | --- | --- |
| Up regulated | 6.42E-07 | 66 | Transcriptional misregulation in cancer | CDKN1A CEBPA CEBPB GADD45G CSF2 GADD45A DDIT3 DDX5 DUSP6 ETV4 ETV5 FCGR1A JMJD1C FOXO1 FLT1 NUPR1 GRIA3 H3F3A H3F3B HHEX HOXA9 ID2 IGFBP3 IL6 CXCL8 ITGB7 LMO2 MEIS1 MLF1 MMP3 MMP9 MYC MYCN GADD45B NGFR PAX5 PBX3 PDGFA SIX4 PPARG PRCC RARA BCL2A1 BCL6 BCL11B HIST2H3D SSX1 ZEB1 TFE3 TRAF1 KDM6A PAX8 IL1R2 NR4A3 HIST1H3A HIST1H3D HIST1H3C HIST1H3E HIST1H3I HIST1H3G HIST1H3J HIST1H3H RUNX2 RUNX1T1 CCNA1 CCND2 |
| Up regulated | 1.12E-06 | 25 | Proteasome | PSMD14 ADRM1 POMP PSMA1 PSMA3 PSMA4 PSMA5 PSMA7 PSMB1 PSMB2 PSMB4 PSMB7 PSMC1 PSMC2 PSMC3 PSMC4 PSMC5 PSMC6 PSMD2 PSMD3 PSMD7 PSMD8 PSMD12 PSMD13 SEM1 |
| Up regulated | 9.65E-06 | 88 | Cytokine-cytokine receptor interaction | CCL26 IL24 CCR7 ACVR1C IL31RA CSF1 CSF2 CSF3 CX3CR1 EDA CLCF1 IL17RA CERS1 MSTN GDF9 AMH AMHR2 IL37 IL36A IL17C XCR1 CXCL3 IL1A IL1B IL3RA IL6 IL6R IL7R CXCL8 CXCR1 IL10RA IL11 IL11RA IL12A IL12B IL12RB2 IL16 IL18 INHBA INHBB INHBC IL31 LIF LTA NGF NGFR NODAL OSM IL20 TNFRSF12A IL23A IL17D IL20RB TNFRSF19 IL36G ACKR3 TNFRSF17 CCL20 CCL22 CCL24 CXCL6 CX3CL1 CRLF2 BMP2 BMP4 BMP6 BMP7 THPO TNFRSF1B TNFSF4 IL1R2 CXCR4 INHBE RELT TSLP TNFSF14 TNFSF13 TNFSF9 TNFRSF10D TNFRSF10B TNFRSF10A IL1RL2 ACVR1 IL1RL1 IL32 TNFRSF8 TNFSF8 GDF15 |
| Up regulated | 0.000164983 | 84 | MAPK signaling pathway | PPP5D1 CACNG2 GADD45G MAP4K1 DUSP10 RASGRP4 PLA2G4E CSF1 GADD45A DDIT3 DUSP1 DUSP2 DUSP4 DUSP5 DUSP6 DUSP7 EFNA2 EPHA2 MECOM FGF1 FGF2 VEGFD RRAS2 FLT1 FLT4 FOS MAPK8IP2 RASGRP3 FGF21 PLA2G4D ANGPT2 MKNK2 NR4A1 HSPA2 HSPA6 HSPA8 HSPB1 IL1A IL1B INSR JUN JUND AREG STMN1 KITLG MYC GADD45B ATF4 NFATC1 NGF NGFR ANGPT4 PDGFA PDGFRB PGF PPM1B PPP3R1 PRKCG MAPK8 MAP2K1 MAP2K3 PTPN7 PTPRR RAC1 RAC2 RAP1A RASGRF1 CACNG6 VEGFA VEGFC CACNA1A CACNA1B CACNA1E CACNB4 CACNG1 CASP3 IKBKG PLA2G4C LAMTOR3 FGF18 CACNA1G CACNA2D2 MAPK8IP1 FGF19 |
| Up regulated | 0.001455269 | 32 | Rheumatoid arthritis | CSF1 CSF2 CTSL FLT1 FOS ATP6V0D2 ATP6V1C2 IL1A IL1B IL6 CXCL8 IL11 IL18 ITGAL ITGB2 JUN MMP1 MMP3 ATP6V1D IL23A ATP6V1H ATP6V1C1 ATP6V1E1 ATP6V0B ATP6V1G2 CCL20 CXCL6 TLR2 VEGFA TNFSF13 ATP6V0E1 ATP6V0D1 |
| Up regulated | 0.002638181 | 88 | Neuroactive ligand-receptor interaction | LPAR6 CGA GPR83 CHRM1 CHRM3 CHRM4 CHRNA2 CHRNA3 CHRNA4 CHRNA5 CHRNB2 RLN3 CNR2 ADM ADORA3 ADRA1B ADRA2B ADRA2C ADRB2 ADRB3 GPR156 DRD4 S1PR1 S1PR3 EDN2 F2R F2RL1 FPR3 GABRG2 GCGR GHRHR GIP GNRH1 LPAR4 GPR35 P2RY8 GRIA1 GRIA3 GRID1 GRIN2A GRIN2D GRM2 GRM4 GRM8 GRPR HCRTR1 HRH1 HTR1D HTR2B HTR7 RXFP4 KISS1 LHB MAS1 MC5R MLN NMB NPY1R NTSR1 OPRD1 OXTR P2RX3 P2RX7 GAL PPY CHRNA9 PRSS3 PYY PTGER1 PTGER2 PTGER3 SCT SSTR2 SSTR3 TACR2 TAC3 TACR1 C5AR1 UCN VIP CALCA GALR3 GALR2 APLN UCN2 GPR50 GLP2R GABBR2 |
| Up regulated | 0.005084693 | 52 | Alcoholism | HDAC5 ADCY5 HIST3H2BB FOSB SHC2 GNAS GNG4 GNG5 GNG7 GNGT2 GRIN2A GRIN2D HIST1H2AE H2AFX H2AFZ HIST1H2BD H3F3A H3F3B MAOA HIST2H2BF ATF4 SHC3 MAP2K1 HIST2H3D HIST2H2AA4 CALM2 HIST1H4I HIST1H2AL HIST1H2AC HIST2H2AA3 HIST1H2BG HIST1H2BN HIST1H2BE HIST1H2BC HIST1H2BO HIST2H2BE HIST1H3A HIST1H3D HIST1H3C HIST1H3E HIST1H3I HIST1H3G HIST1H3J HIST1H3H HIST1H4K HIST1H4H HIST1H4E HIST1H2BK HIST1H2BJ HIST3H2A CREB5 HDAC9 |
| Up regulated | 0.008512374 | 21 | Legionellosis | CLK1 EEF1A2 CXCL3 HSPA2 HSPA6 HSPA8 HSPD1 IL1B IL6 CXCL8 IL12A IL12B IL18 ITGB2 ARF1 NLRC4 RAB1A BNIP3 TLR2 VCP CASP3 |
| Up regulated | 0.009094526 | 30 | TGF-beta signaling pathway | FST LEFTY1 ACVR1C BAMBI AMH AMHR2 ID2 ID4 INHBA INHBB INHBC SMAD7 SMAD9 MYC NODAL PITX2 PPP2CA PPP2CB SMURF1 HAMP GREM2 BMP2 SKP1 BMP4 BMP6 BMP7 TGIF1 INHBE ACVR1 NOG |
| Up regulated | 0.009094526 | 124 | Pathways in cancer | BCL2L11 FRAT1 LPAR6 CDKN1A CEBPA GADD45G ADCY5 EGLN3 RASGRP4 CKS1B CKS2 GSTO2 CTNNB1 GADD45A NQO1 JAG1 ADCY4 ESR1 ETS1 MECOM F2R FGF1 FGF2 VEGFD LAMB4 FOXO1 FLT4 HEY1 HEY2 FOS DAPK2 ALK RASGRP3 FGF21 BBC3 GLI1 GLI3 GNAS GNG4 GNG5 GNG7 GNGT2 LAMA1 LPAR4 GSTM3 HMOX1 HES1 HSP90AA1 HSP90AB1 IL3RA IL6 IL6R IL7R CXCL8 IL12A IL12B IL12RB2 JUN LAMA2 LAMB3 KITLG MGST1 MMP1 MMP9 MYC GADD45B NFE2L2 NKX3-1 NOS2 NOTCH1 NOTCH4 PDGFA IL23A PDGFRB PGF PIM1 PMAIP1 DLL4 PPARG PRKCG MAPK8 MAP2K1 PTGER1 PTGER2 PTGER3 PTGS2 RAC1 RAC2 RALA RARA BCL2 BID SHH BMP2 SKP1 BMP4 ELOC TCF7 TRAF1 TXNRD1 VEGFA VEGFC WNT1 WNT2 WNT11 WNT2B PAX8 CXCR4 CALM2 WNT5B CAMK2D FZD8 FZD9 CASP3 IKBKG RUNX1T1 FGF18 CCNA1 CCND2 CCNE1 ARHGEF1 GSTO1 FGF19 RBX1 |
| Up regulated | 0.009094526 | 41 | Fluid shear stress and atherosclerosis | KLF2 GSTO2 CTNNB1 CTSL NQO1 DUSP1 FOS GSTM3 HMOX1 HSP90AA1 HSP90AB1 IL1A IL1B ITGB3 JUN MEF2A MGST1 MMP9 NCF2 NFE2L2 NOS3 NPPC PDGFA MAPK8 RAC1 RAC2 BCL2 BMP4 NCF1 THBD ACTG1 TXN SUMO1 VEGFA IL1R2 CALM2 IKBKG SQSTM1 ACVR1 ARHGEF2 GSTO1 |
| Down regulated | 6.73E-10 | 255 | Metabolic pathways | GNE PIGK DHRS9 TCIRG1 B3GNT3 BPNT1 POMT1 MAN1A2 B4GAT1 GALNT6 MAN1B1 B4GALT7 MGAT4A EARS2 GGT6 B3GALT6 PIGU CMPK2 GALM COX11 COX15 CPOX HGSNAT NANP ALG10B CYC1 MBOAT1 ATP6V0E2 CYP2J2 CYP51A1 DGKQ DBT DDC DHCR7 DHCR24 DHFR DHODH DLAT DLD AGL DNMT1 DPAGT1 DPYD EHHADH FUK PIKFYVE DHFR2 HACD2 EPHX2 ALAD EXT2 EXTL2 EXTL3 ACSL1 ALDH1B1 FASN ALDH3B1 FKTN ALDH3B2 FDFT1 FDPS FECH ALDH3A2 INPP5F SACM1L PLCH1 LPIN1 GANAB PIP5K1C QPRT NNT PIGN AMACR ALPP FUT2 LCLAT1 GAA PLA2G4F GALC GALNT2 GANC GLCE B3GAT3 OPLAH ACAD8 ST6GALNAC4 GLDC MCAT PIGW ANPEP C1GALT1C1 CYP2S1 GMPPB GMPPA ALG6 GYS1 HADHA HLCS HMBS HMGCR ACACB MMAB HSD17B1 HSD17B4 NDST1 B4GALNT4 ACADM IDH2 IDUA ACADS ACADSB IDO1 INPP4A INPP5D STT3A ITPKB IVD KHK ENO4 ACAT2 LAMA5 HACD4 ARSB MAN2A2 MGAT3 MPI MT-CO1 MT-CO3 MTHFD1 MTHFR MT-ND4L MT-ND5 MT-ND6 MTR MVD MVK NAGA NAGLU ACLY ACO1 NME3 NNMT PHOSPHO2 OCRL OGDH RRM2B PC COQ6 MECR PLCE1 CSAD PIGT ENPP1 COQ3 GALNT7 PFKL PGM1 PIGF PIK3C2B PIK3CB PIK3CD PI4KA ACP2 PLCB2 PLCB3 PANK1 PLD1 BCO1 DPM3 HMGCLL1 UGT1A7 UGT1A6 UGT1A1 IMPAD1 UCKL1 PPOX PNPO QRSL1 SPTLC3 PPT1 DHTKD1 GALNT10 POMGNT1 SMPD4 PIGV CSGALNACT1 ACSS2 ALG1 AGPAT3 BDH2 PDXP PTGIS GPAM GBA2 ALDH18A1 PCYT2 QARS BCKDHB ACSM3 MSMO1 GALNT11 XYLT2 ACOT1 SHMT1 ST3GAL2 LPIN3 SQLE SUOX BTD TK2 TM7SF2 TYMS UROS MOGS CAD VKORC1 ALG12 ALDH5A1 THTPA PANK3 ALG9 PGAP1 PTGES2 HSD3B7 PNPLA3 COASY PTDSS2 NUDT12 PIP4K2B POMK MRI1 PIGO ALG10 DGKZ SELENOI ALDH4A1 JMJD7-PLA2G4B HYAL2 DGAT1 GPAA1 CDS2 FPGT SUCLG2 ALDH1A2 GYG2 PAPSS2 PIGQ MTMR4 G6PC3 PIGM PIGB H6PD GDA FIG4 XYLB |
| Down regulated | 6.02E-06 | 33 | Small cell lung cancer | CDKN2B COL4A1 COL4A2 COL4A4 COL4A5 COL4A6 E2F1 E2F2 AKT1 AKT2 FN1 APAF1 BIRC2 BIRC3 IKBKB ITGA2B ITGA3 ITGAV LAMA5 LAMB1 LAMB2 LAMC1 NFKB1 NFKBIA PIK3CB PIK3CD RB1 CCND1 RXRA SKP2 TP53 TRAF5 PIK3R3 |
| Down regulated | 1.17E-05 | 39 | Lysosome | AP3S2 TCIRG1 AP3M2 AP1S1 HGSNAT CTSK CTSS AP1B1 AGA ABCA2 ARSG GGA3 GAA MFSD8 GALC GGA1 SLC17A5 AP3M1 GM2A SUMF1 IDUA LIPA ARSA ARSB MANBA NAGA NAGLU SLC11A2 ACP2 PPT1 SORT1 GNPTAB AP3B1 CTSF AP1G2 AP1M1 AP3D1 AP4M1 ENTPD4 |
| Down regulated | 4.77E-05 | 45 | Cellular senescence | RAD50 HIPK3 CDKN2B CHEK2 MAPK14 E2F1 E2F2 HIPK1 AKT1 AKT2 FOXM1 MTOR ITPR2 ITPR3 SMAD3 MRE11 NBN ATM NFATC3 NFATC4 NFKB1 CALML5 PIK3CB PIK3CD ATR PPP3CB MAPK3 MAP2K6 RB1 RBBP4 RBL1 RBL2 CCND1 MAPK12 TGFB2 TGFBR2 TP53 TSC2 CALM3 CAPN1 PIK3R3 CCNA2 CCNB1 CCNB2 CDK1 |
| Down regulated | 7.84E-05 | 37 | Cell cycle | CDKN2B DBF4 CHEK2 E2F1 E2F2 ANAPC2 ANAPC4 MAD2L1 SMAD3 MCM3 MCM6 ATM ANAPC5 PLK1 ATR PRKDC RAD21 RB1 RBL1 RBL2 CCND1 ANAPC1 SKP2 BUB1B TGFB2 TP53 SMC1A CDC7 CCNA2 CCNB1 PKMYT1 SMC3 CCNB2 ESPL1 CDK1 CDC20 CDC25B |
| Down regulated | 0.000119255 | 31 | Progesterone-mediated oocyte maturation | ADCY3 ADCY6 ADCY9 MAPK14 AKT1 AKT2 RPS6KA6 ANAPC2 ANAPC4 KIF22 MAD2L1 SPDYE3 SPDYE2 SPDYE5 ANAPC5 PIK3CB PIK3CD PLK1 PRKACA MAPK3 RPS6KA2 MAPK12 ANAPC1 AURKA PIK3R3 CCNA2 CCNB1 PKMYT1 CCNB2 CDK1 CDC25B |
| Down regulated | 0.000252176 | 17 | Homologous recombination | POLD3 TOPBP1 RAD54B MRE11 NBN ATM POLD1 BARD1 RAD51D BLM BRCA1 XRCC3 BRCC3 BRIP1 ABRAXAS1 RAD54L TOP3B |
| Down regulated | 0.000252176 | 73 | Human papillomavirus infection | TCIRG1 CHAD COL4A1 COL4A2 COL4A4 COL4A5 COL4A6 ATP6V0E2 DVL2 E2F1 AKT1 AKT2 FN1 MTOR TUBG2 TBK1 APC IFNAR1 IFNAR2 IKBKB ITGA2B ITGA3 ITGAV ITGB4 ITGB5 ITGB6 LAMA5 LAMB1 LAMB2 LAMC1 LFNG LLGL1 ATM NFKB1 NOTCH2 NOTCH3 PARD6A PIK3CB PIK3CD WNT4 ATR PPP2R5D PRKACA MAPK3 RB1 RBL1 RBL2 CCND1 RPS6KB1 MPP5 CREB3L2 STAT1 THBS2 THBS3 TLR3 TP53 TSC2 TUBG1 TYK2 WNT9A FZD5 FZD3 WNT10A FZD1 FZD6 MAML2 PIK3R3 OASL TRADD FADD CCNA2 IKBKE MAML1 |
| Down regulated | 0.000290344 | 20 | Fanconi anemia pathway | POLI ERCC4 FANCA FANCC FANCD2 FANCF FANCG UBE2T POLH ATR FANCI FANCM REV3L BLM BRCA1 RMI1 FAAP100 BRIP1 SLX4 TOP3B |
| Down regulated | 0.000641864 | 34 | Oocyte meiosis | STAG3 ADCY3 ADCY6 ADCY9 RPS6KA6 ANAPC2 ANAPC4 ITPR2 ITPR3 MAD2L1 SPDYE3 SPDYE2 SPDYE5 ANAPC5 CALML5 PLK1 PPP2R5D PPP3CB PRKACA MAPK3 RPS6KA2 MAPK12 ANAPC1 AURKA CALM3 CAMK2G SMC1A CCNB1 PKMYT1 SMC3 CCNB2 ESPL1 CDK1 CDC20 |
| Down regulated | 0.000646492 | 12 | Glycosylphosphatidylinositol (GPI)-anchor biosynthesis | PIGU PIGN PIGW PIGT PIGF PIGV PGAP1 PIGZ PIGO GPAA1 PIGQ PIGM |
| Down regulated | 0.000646492 | 19 | Fatty acid metabolism | CPT1C CPT2 EHHADH HACD2 ACSL1 FASN MCAT HADHA HSD17B4 ACADM ACADS ACADSB ACAT2 FADS1 HACD4 MECR PPT1 SCD FADS2 |

Table 2. The genes involved in the significantly up and down-regulated pathways.

| Direction | adj.Pval | nGenes | Pathways | Genes |
| --- | --- | --- | --- | --- |
| Down regulated | 1.92E-20 | 480 | Nucleotide binding | RHOBTB2 TUBG2 MSH2 POLA1 TUBD1 NNT ARL6 SIL1 AARS2 RHOT1 TUBG1 MMACHC RHOT2 PFKL RECQL4 ACOT11 RHOBTB3 LDHD MTHFR DHFR2 D2HGDH TUBB TMEM173 PDE4B DPYD BLM MSH5 DHFR RTEL1 NOX1 ACSS2 P2RX4 AKT1 UHMK1 ENPP1 CDKL5 PPP5C RAB27B WNK1 PDK3 PNPLA3 FOXRED2 SAMHD1 DMPK AKT2 ABCA2 BMPR1A PNPO OAS3 RAB5B STK38 ICK HSD17B8 HMGCR WDR77 GBP1 IDE PANK3 CAT STEAP4 LATS1 MYH10 ACVR1B PIF1 TP53 MAP3K6 CDC42BPA PPIP5K2 MMAA PANK1 SMARCA5 RRAGA HOXB13 LRWD1 BDH2 KDM1B MAP4K2 TAP1 CDC42BPG RAB1B MYO18A RABL6 MAP3K5 NUDT16 TAP2 ERCC6 TSSK4 CFTR DHX33 LIG3 ACSM3 REV3L LARS2 DDX11 AQR NLRP2 EHD2 RTEL1-TNFRSF6B DNAH5 YTHDC2 H6PD PIK3CB ENTPD2 ATP11B ALDH18A1 POLD1 ADCK1 ABCA7 PMS1 PKN2 ERBB3 MYLK PRKCQ FGFR2 MYO9A IARS2 ROCK1 FGFR3 COASY ATP11A ATP2B1 RPS6KA2 HLTF PRKACA RPS6KA6 SMC1A NSF TUBA3D MCM6 ACACB TOP2B UBE2T KIF22 RABL2B ATP8B1 HADHA CAD ATRX RAD54L MAST2 EIF2AK1 AURKA OAS1 KIF16B DHX32 KIF4A DLD XYLB MAP3K1 NUBP2 CDC7 P2RX6 DDX17 RAB36 TTLL12 IFT27 NIN DICER1 MTHFD1 KIF3B VWA8 MAPK3 LONP2 DHODH NME3 CLCN7 EEF2K EARS2 BCKDK CSK IKBKB SQLE ERCC2 MYH14 TYK2 LIG1 RAB3D ATP13A1 NOD1 DNM1 DDX58 SMC3 RPS6KB1 RECQL5 DHX58 ABCC3 MAP2K6 HIPK3 MVK ACAD10 OAS2 RFC5 FRK HINT3 MAPK14 MCM3 MDN1 RAB23 ASCC3 PTK7 CUL9 PRPF4B KIF20A RAD50 SMC4 PRKAR2A ABCC5 NEK4 RTKN TTL PIKFYVE ACTR1B DNAJC27 IFIH1 PASK STK25 DHCR24 FAM20B TTF2 ACADM CDK18 DARS2 NEK2 KIF14 AKAP7 ABCG2 NEK9 TTLL5 COQ6 ATL2 HELLS APAF1 KIF18A GPSM2 CIT ACADS NRK COQ8B CHD6 ABCC10 MCM8 XRCC3 TRAP1 PKMYT1 CHTF18 PEX1 EIF2AK4 MYO5C IVD TEP1 GTPBP3 SLC27A1 QRSL1 TRPM4 HELZ2 PAK4 DNMT1 UBE4B KIF3A ACLY TOP2A MCCC2 PRKAA1 EEFSEC FIGNL1 PIK3C2B CSNK1G2 DPH6 CMPK2 OASL DYNC1LI2 TTLL4 NEK3 ALPK3 BRIP1 SMC2 DDR1 NARS2 NEK1 DDX60 KIF23 KHK ADCY3 ACTR1A KIF11 KIF20B ENTPD1 DNA2 CENPE MMAB RAB15 NLRC5 KSR1 AFG3L2 ERBB2 TARS2 ATP8B2 SLC27A3 HCN3 ITPKB PARP1 SNRNP200 RABL2A ABCA12 DGKQ TBCK DDX46 RARS2 SHPRH EPHA1 RAB19 TAF1 CAMK2G MKI67 MTG1 DGKZ ATM HYOU1 DHX37 NUBPL ACAD8 ACSL1 GPD1L LRRK1 ABCA5 OBSCN SLFN13 MOV10 CDK20 UBE2L6 AIFM1 BUB1B FUK MYO1E HLCS ATP13A2 STARD9 GNE ARHGAP35 PKN3 TAOK1 NLRX1 ADCY9 PARS2 GBP4 SMC6 SMARCAD1 DHX57 HIPK1 STK36 TGFBR2 RYK KIF15 MST1R RAD54L2 FASTK SYK WNK2 ATP7A TK2 HSP90B1 KIF7 PLK1 ACSF2 SLC27A4 CDK12 MVD KIFC2 RAB26 ABCA3 TRANK1 PBK PPIP5K1 CSNK1G1 ARL13B FASN DUS1L CDK1 KIF5B MFN1 CLCN5 KSR2 PIK3CD SLFNL1 RAB33B QARS SUCLG2 SLFN11 DCAKD GRK2 GMPPB PC ADCY6 DDX23 ATR GK5 ARL10 MYO1D TYMS POLE DALRD3 MCMDC2 OPLAH RMI1 FARSA CIITA BBS10 AATK ACBD4 IDH2 ERCC6L2 UBA7 GDPGP1 EPHA10 MX2 EP400 TBK1 CHEK2 SRPK3 UBE2G2 NRBP2 CDK10 RAD51D ROR1 POMK PIP5K1C KIF18B KIF24 ERCC6L DYNC2H1 FANCM PALM3 RAB42 MAPK12 SBK1 ENTPD8 ACADSB DYNC1H1 RAD54B SHPK RAB40C TXNRD3 ATAD3A MYO1C SPG7 GRK6 RPS6KL1 UCKL1 PAPSS2 ABCA4 CDC42BPB MTOR MAP3K3 BMPR2 SKIV2L DHX16 DDX47 KIF4B PI4KA MICAL3 MARS2 PRKDC FPGT IKBKE STRADA AARSD1 ACACA DHRS11 PIP4K2B MYO19 HSD17B1 ALDH1B1 TM7SF2 ETFDH DHCR7 FAM114A2 CHST12 HS3ST5 TPX2 PPOX |
| Down regulated | 3.23E-20 | 362 | ATP binding | MSH2 AARS2 PFKL RECQL4 BLM MSH5 RTEL1 P2RX4 AKT1 UHMK1 CDKL5 PPP5C WNK1 PDK3 DMPK AKT2 ABCA2 BMPR1A OAS3 STK38 ICK IDE PANK3 LATS1 MYH10 ACVR1B PIF1 TP53 MAP3K6 CDC42BPA PPIP5K2 SMARCA5 RHOBTB3 MAP4K2 TAP1 CDC42BPG MYO18A MAP3K5 ENPP1 TAP2 ERCC6 TSSK4 CFTR DHX33 LIG3 ACSM3 LARS2 DDX11 AQR NLRP2 EHD2 RTEL1-TNFRSF6B DNAH5 YTHDC2 PIK3CB ENTPD2 ATP11B ALDH18A1 ADCK1 ABCA7 PMS1 PKN2 ERBB3 MYLK PRKCQ FGFR2 MYO9A IARS2 ROCK1 FGFR3 COASY ATP11A ATP2B1 RPS6KA2 HLTF PRKACA RPS6KA6 SMC1A NSF MCM6 ACACB TOP2B UBE2T KIF22 ATP8B1 CAD ATRX RAD54L MAST2 EIF2AK1 AURKA OAS1 KIF16B DHX32 KIF4A XYLB MAP3K1 NUBP2 CDC7 P2RX6 DDX17 TTLL12 DICER1 MTHFD1 KIF3B VWA8 MAPK3 LONP2 NME3 CLCN7 EEF2K EARS2 BCKDK CSK IKBKB ERCC2 MYH14 TYK2 LIG1 ATP13A1 NOD1 DDX58 SMC3 RPS6KB1 RECQL5 DHX58 ABCC3 MAP2K6 HIPK3 MVK OAS2 RFC5 FRK MAPK14 MCM3 MDN1 ASCC3 PTK7 CUL9 PRPF4B KIF20A RAD50 SMC4 ABCC5 NEK4 TTL PIKFYVE ACTR1B IFIH1 PASK STK25 FAM20B TTF2 CDK18 DARS2 NEK2 KIF14 ABCG2 NEK9 TTLL5 HELLS APAF1 KIF18A CIT NRK COQ8B CHD6 ABCC10 MCM8 XRCC3 TRAP1 PKMYT1 CHTF18 PEX1 EIF2AK4 MYO5C TEP1 QRSL1 TRPM4 HELZ2 PAK4 UBE4B ACSS2 KIF3A ACLY TOP2A MCCC2 PRKAA1 FIGNL1 PIK3C2B CSNK1G2 DPH6 CMPK2 OASL DYNC1LI2 TTLL4 NEK3 ALPK3 BRIP1 SMC2 DDR1 NARS2 NEK1 DDX60 KIF23 KHK ADCY3 ACTR1A KIF11 KIF20B ENTPD1 DNA2 CENPE MMAB NLRC5 KSR1 AFG3L2 ERBB2 TARS2 ATP8B2 SLC27A3 ITPKB SNRNP200 ABCA12 DGKQ TBCK DDX46 RARS2 SHPRH EPHA1 TAF1 CAMK2G MKI67 DGKZ ATM HYOU1 DHX37 NUBPL ACSL1 PANK1 LRRK1 ABCA5 OBSCN SLFN13 MOV10 CDK20 UBE2L6 BUB1B FUK MYO1E HLCS ATP13A2 STARD9 GNE PKN3 TAOK1 NLRX1 ADCY9 PARS2 SMC6 SMARCAD1 DHX57 HIPK1 STK36 TGFBR2 RYK KIF15 MST1R RAD54L2 FASTK SYK WNK2 ATP7A TK2 HSP90B1 KIF7 PLK1 ACSF2 CDK12 MVD KIFC2 ABCA3 TRANK1 PBK PPIP5K1 CSNK1G1 CDK1 KIF5B CLCN5 KSR2 PIK3CD SLFNL1 QARS SUCLG2 SLFN11 DCAKD GRK2 PC ADCY6 DDX23 ATR GK5 MYO1D DALRD3 MCMDC2 OPLAH FARSA CIITA BBS10 AATK ERCC6L2 UBA7 EPHA10 EP400 TBK1 CHEK2 SRPK3 UBE2G2 NRBP2 CDK10 RAD51D ROR1 POMK PIP5K1C KIF18B KIF24 ERCC6L DYNC2H1 FANCM PALM3 MAPK12 SBK1 ENTPD8 DYNC1H1 RAD54B SHPK ATAD3A MYO1C SPG7 GRK6 RPS6KL1 UCKL1 PAPSS2 ABCA4 CDC42BPB MTOR MAP3K3 BMPR2 SKIV2L DHX16 DDX47 KIF4B PI4KA MARS2 PRKDC IKBKE STRADA AARSD1 ACACA PIP4K2B MYO19 TPX2 |
| Down regulated | 3.23E-20 | 372 | Adenyl ribonucleotide binding | MSH2 AARS2 PFKL RECQL4 ACOT11 TMEM173 PDE4B BLM MSH5 RTEL1 ACSS2 P2RX4 AKT1 UHMK1 ENPP1 CDKL5 PPP5C WNK1 PDK3 PNPLA3 DMPK AKT2 ABCA2 BMPR1A OAS3 STK38 ICK IDE PANK3 LATS1 MYH10 ACVR1B PIF1 TP53 MAP3K6 CDC42BPA PPIP5K2 PANK1 SMARCA5 RHOBTB3 MAP4K2 TAP1 CDC42BPG MYO18A MAP3K5 TAP2 ERCC6 TSSK4 CFTR DHX33 LIG3 ACSM3 LARS2 DDX11 AQR NLRP2 EHD2 RTEL1-TNFRSF6B DNAH5 YTHDC2 PIK3CB ENTPD2 ATP11B ALDH18A1 ADCK1 ABCA7 PMS1 PKN2 ERBB3 MYLK PRKCQ FGFR2 MYO9A IARS2 ROCK1 FGFR3 COASY ATP11A ATP2B1 RPS6KA2 HLTF PRKACA RPS6KA6 SMC1A NSF MCM6 ACACB TOP2B UBE2T KIF22 ATP8B1 HADHA CAD ATRX RAD54L MAST2 EIF2AK1 AURKA OAS1 KIF16B DHX32 KIF4A XYLB MAP3K1 NUBP2 CDC7 P2RX6 DDX17 TTLL12 DICER1 MTHFD1 KIF3B VWA8 MAPK3 LONP2 NME3 CLCN7 EEF2K EARS2 BCKDK CSK IKBKB ERCC2 MYH14 TYK2 LIG1 ATP13A1 NOD1 DDX58 SMC3 RPS6KB1 RECQL5 DHX58 ABCC3 MAP2K6 HIPK3 MVK OAS2 RFC5 FRK MAPK14 MCM3 MDN1 ASCC3 PTK7 CUL9 PRPF4B KIF20A RAD50 SMC4 PRKAR2A ABCC5 NEK4 TTL PIKFYVE ACTR1B IFIH1 PASK STK25 FAM20B TTF2 CDK18 DARS2 NEK2 KIF14 ABCG2 NEK9 TTLL5 HELLS APAF1 KIF18A CIT NRK COQ8B CHD6 ABCC10 MCM8 XRCC3 TRAP1 PKMYT1 CHTF18 PEX1 EIF2AK4 MYO5C TEP1 QRSL1 TRPM4 HELZ2 PAK4 UBE4B KIF3A ACLY TOP2A MCCC2 PRKAA1 FIGNL1 PIK3C2B CSNK1G2 DPH6 CMPK2 OASL DYNC1LI2 TTLL4 NEK3 ALPK3 BRIP1 SMC2 DDR1 NARS2 NEK1 DDX60 KIF23 KHK ADCY3 ACTR1A KIF11 KIF20B ENTPD1 DNA2 CENPE MMAB NLRC5 KSR1 AFG3L2 ERBB2 TARS2 ATP8B2 SLC27A3 HCN3 ITPKB SNRNP200 ABCA12 DGKQ TBCK DDX46 RARS2 SHPRH EPHA1 TAF1 CAMK2G MKI67 DGKZ ATM HYOU1 DHX37 NUBPL ACSL1 LRRK1 ABCA5 OBSCN SLFN13 MOV10 CDK20 UBE2L6 BUB1B FUK MYO1E HLCS ATP13A2 STARD9 GNE PKN3 TAOK1 NLRX1 ADCY9 PARS2 SMC6 SMARCAD1 DHX57 HIPK1 STK36 TGFBR2 RYK KIF15 MST1R RAD54L2 FASTK SYK WNK2 ATP7A TK2 HSP90B1 KIF7 PLK1 ACSF2 CDK12 MVD KIFC2 ABCA3 TRANK1 PBK PPIP5K1 CSNK1G1 CDK1 KIF5B CLCN5 KSR2 PIK3CD SLFNL1 QARS SUCLG2 SLFN11 DCAKD GRK2 PC ADCY6 DDX23 ATR GK5 MYO1D DALRD3 MCMDC2 OPLAH FARSA CIITA BBS10 AATK ACBD4 ERCC6L2 UBA7 EPHA10 EP400 TBK1 CHEK2 SRPK3 UBE2G2 NRBP2 CDK10 RAD51D ROR1 POMK PIP5K1C KIF18B KIF24 ERCC6L DYNC2H1 FANCM PALM3 MAPK12 SBK1 ENTPD8 DYNC1H1 RAD54B SHPK ATAD3A MYO1C SPG7 GRK6 RPS6KL1 UCKL1 PAPSS2 ABCA4 CDC42BPB MTOR MAP3K3 BMPR2 SKIV2L DHX16 DDX47 KIF4B PI4KA MARS2 PRKDC IKBKE STRADA AARSD1 ACACA PIP4K2B MYO19 CHST12 HS3ST5 TPX2 |
| Down regulated | 3.87E-20 | 373 | Adenyl nucleotide binding | MSH2 SIL1 AARS2 PFKL RECQL4 ACOT11 TMEM173 PDE4B BLM MSH5 RTEL1 ACSS2 P2RX4 AKT1 UHMK1 ENPP1 CDKL5 PPP5C WNK1 PDK3 PNPLA3 DMPK AKT2 ABCA2 BMPR1A OAS3 STK38 ICK IDE PANK3 LATS1 MYH10 ACVR1B PIF1 TP53 MAP3K6 CDC42BPA PPIP5K2 PANK1 SMARCA5 RHOBTB3 MAP4K2 TAP1 CDC42BPG MYO18A MAP3K5 TAP2 ERCC6 TSSK4 CFTR DHX33 LIG3 ACSM3 LARS2 DDX11 AQR NLRP2 EHD2 RTEL1-TNFRSF6B DNAH5 YTHDC2 PIK3CB ENTPD2 ATP11B ALDH18A1 ADCK1 ABCA7 PMS1 PKN2 ERBB3 MYLK PRKCQ FGFR2 MYO9A IARS2 ROCK1 FGFR3 COASY ATP11A ATP2B1 RPS6KA2 HLTF PRKACA RPS6KA6 SMC1A NSF MCM6 ACACB TOP2B UBE2T KIF22 ATP8B1 HADHA CAD ATRX RAD54L MAST2 EIF2AK1 AURKA OAS1 KIF16B DHX32 KIF4A XYLB MAP3K1 NUBP2 CDC7 P2RX6 DDX17 TTLL12 DICER1 MTHFD1 KIF3B VWA8 MAPK3 LONP2 NME3 CLCN7 EEF2K EARS2 BCKDK CSK IKBKB ERCC2 MYH14 TYK2 LIG1 ATP13A1 NOD1 DDX58 SMC3 RPS6KB1 RECQL5 DHX58 ABCC3 MAP2K6 HIPK3 MVK OAS2 RFC5 FRK MAPK14 MCM3 MDN1 ASCC3 PTK7 CUL9 PRPF4B KIF20A RAD50 SMC4 PRKAR2A ABCC5 NEK4 TTL PIKFYVE ACTR1B IFIH1 PASK STK25 FAM20B TTF2 CDK18 DARS2 NEK2 KIF14 ABCG2 NEK9 TTLL5 HELLS APAF1 KIF18A CIT NRK COQ8B CHD6 ABCC10 MCM8 XRCC3 TRAP1 PKMYT1 CHTF18 PEX1 EIF2AK4 MYO5C TEP1 QRSL1 TRPM4 HELZ2 PAK4 UBE4B KIF3A ACLY TOP2A MCCC2 PRKAA1 FIGNL1 PIK3C2B CSNK1G2 DPH6 CMPK2 OASL DYNC1LI2 TTLL4 NEK3 ALPK3 BRIP1 SMC2 DDR1 NARS2 NEK1 DDX60 KIF23 KHK ADCY3 ACTR1A KIF11 KIF20B ENTPD1 DNA2 CENPE MMAB NLRC5 KSR1 AFG3L2 ERBB2 TARS2 ATP8B2 SLC27A3 HCN3 ITPKB SNRNP200 ABCA12 DGKQ TBCK DDX46 RARS2 SHPRH EPHA1 TAF1 CAMK2G MKI67 DGKZ ATM HYOU1 DHX37 NUBPL ACSL1 LRRK1 ABCA5 OBSCN SLFN13 MOV10 CDK20 UBE2L6 BUB1B FUK MYO1E HLCS ATP13A2 STARD9 GNE PKN3 TAOK1 NLRX1 ADCY9 PARS2 SMC6 SMARCAD1 DHX57 HIPK1 STK36 TGFBR2 RYK KIF15 MST1R RAD54L2 FASTK SYK WNK2 ATP7A TK2 HSP90B1 KIF7 PLK1 ACSF2 CDK12 MVD KIFC2 ABCA3 TRANK1 PBK PPIP5K1 CSNK1G1 CDK1 KIF5B CLCN5 KSR2 PIK3CD SLFNL1 QARS SUCLG2 SLFN11 DCAKD GRK2 PC ADCY6 DDX23 ATR GK5 MYO1D DALRD3 MCMDC2 OPLAH FARSA CIITA BBS10 AATK ACBD4 ERCC6L2 UBA7 EPHA10 EP400 TBK1 CHEK2 SRPK3 UBE2G2 NRBP2 CDK10 RAD51D ROR1 POMK PIP5K1C KIF18B KIF24 ERCC6L DYNC2H1 FANCM PALM3 MAPK12 SBK1 ENTPD8 DYNC1H1 RAD54B SHPK ATAD3A MYO1C SPG7 GRK6 RPS6KL1 UCKL1 PAPSS2 ABCA4 CDC42BPB MTOR MAP3K3 BMPR2 SKIV2L DHX16 DDX47 KIF4B PI4KA MARS2 PRKDC IKBKE STRADA AARSD1 ACACA PIP4K2B MYO19 CHST12 HS3ST5 TPX2 |
| Down regulated | 2.67E-18 | 534 | Small molecule binding | RHOBTB2 TUBG2 MSH2 ITPR3 POLA1 ERLIN1 TUBD1 FOLR1 NNT ARL6 GLE1 SIL1 ITPR2 AARS2 RHOT1 TUBG1 MMACHC DBT RHOT2 PFKL ERLIN2 PROM2 RECQL4 ACOT11 RHOBTB3 LDHD SCARA3 SLC19A1 SHMT1 MTHFR GLDC DHFR2 D2HGDH TUBB TMEM173 PDE4B DPYD BLM MSH5 DHFR RTEL1 NOX1 ACSS2 P2RX4 AKT1 UHMK1 TYMS ENPP1 CDKL5 PPP5C RAB27B WNK1 PDK3 HYAL2 PNPLA3 FOXRED2 SAMHD1 DMPK AKT2 OGDH ENG ABCA2 BMPR1A PNPO OAS3 RAB5B STK38 ICK HSD17B8 HMGCR WDR77 GBP1 IDE PANK3 CAT STEAP4 LATS1 MYH10 ACVR1B MMAB PIF1 TP53 MAP3K6 XPR1 CDC42BPA PPIP5K2 ASTN2 MMAA PANK1 SMARCA5 RRAGA HOXB13 HLCS LRWD1 BDH2 STARD4 KDM1B HIF1AN MAP4K2 TAP1 CDC42BPG RAB1B GRIN1 ADAP2 RXRA MYO18A RABL6 MAP3K5 NUDT16 TAP2 ERCC6 UGT1A1 UGT1A7 TSSK4 CFTR DHX33 LIG3 ACSM3 REV3L LARS2 POLR3B DDX11 AQR NLRP2 GRAMD1B EHD2 RTEL1-TNFRSF6B DNAH5 YTHDC2 H6PD PIK3CB ENTPD2 ATP11B ALDH18A1 POLD1 ADCK1 ABCA7 PMS1 PKN2 ERBB3 MYLK PRKCQ FGFR2 MYO9A IARS2 ROCK1 FGFR3 COASY ATP11A ATP2B1 RPS6KA2 HLTF PRKACA RPS6KA6 SMC1A HMMR NSF TUBA3D MCM6 SLC46A1 ACACB TOP2B UBE2T SLC1A3 KIF22 RABL2B ATP8B1 HADHA CAD ATRX RAD54L MAST2 EIF2AK1 AURKA OAS1 KIF16B DHX32 KIF4A DLD XYLB MAP3K1 NUBP2 CDC7 P2RX6 DDX17 RAB36 TTLL12 IFT27 NIN DICER1 MTHFD1 KIF3B VWA8 MAPK3 LONP2 DHODH NME3 CLCN7 EEF2K EARS2 BCKDK CSK IKBKB SQLE GYS1 ERCC2 ILVBL MYH14 TYK2 LIG1 RAB3D ATP13A1 NOD1 SLC1A1 DNM1 DDX58 SMC3 RPS6KB1 RECQL5 DHX58 ABCC3 MAP2K6 MANBA HIPK3 ACCS MVK ACAD10 OAS2 RFC5 FRK HINT3 MAPK14 MCM3 MDN1 RAB23 ASCC3 PTK7 CUL9 PRPF4B KIF20A RAD50 SMC4 PRKAR2A ABCC5 NEK4 RTKN TTL PIKFYVE ACTR1B DNAJC27 IFIH1 PASK STK25 DHCR24 FAM20B TTF2 MTR ACADM CDK18 P3H1 DARS2 NEK2 KIF14 AKAP7 ABCG2 NEK9 TTLL5 COQ6 ATL2 HELLS APAF1 KIF18A GPSM2 CIT ACADS NRK COQ8B CHD6 ABCC10 MCM8 XRCC3 TRAP1 PKMYT1 CHTF18 PEX1 EIF2AK4 MYO5C ALDH1A2 IVD TEP1 GTPBP3 SLC27A1 QRSL1 TRPM4 HELZ2 PAK4 DNMT1 UBE4B KIF3A ACLY TOP2A MCCC2 PRKAA1 EEFSEC FIGNL1 DDC PIK3C2B CSNK1G2 DPH6 CMPK2 OASL DYNC1LI2 TTLL4 NEK3 ALPK3 BRIP1 SMC2 DDR1 NARS2 NEK1 DDX60 KIF23 STRA6 KHK ADCY3 ACTR1A KIF11 KIF20B ENTPD1 DNA2 CENPE RBP5 CSAD RAB15 NLRC5 KSR1 AFG3L2 ERBB2 TARS2 ATP8B2 S100A8 SLC27A3 HCN3 ITPKB PARP1 SNRNP200 RABL2A ABCA12 DGKQ TBCK DDX46 RARS2 SHPRH EPHA1 RAB19 TAF1 CAMK2G MKI67 MTG1 DGKZ ATM HYOU1 DHX37 NUBPL ACAD8 ACSL1 GPD1L LRRK1 ABCA5 OBSCN SLFN13 MOV10 CDK20 UBE2L6 AIFM1 BUB1B FUK MYO1E ATP13A2 STARD9 GNE ARHGAP35 PKN3 TAOK1 NLRX1 ADCY9 PARS2 GBP4 SMC6 SMARCAD1 DHX57 HIPK1 STK36 ADORA1 TGFBR2 RYK KIF15 MST1R RAD54L2 FASTK SYK WNK2 ATP7A TK2 HSP90B1 KIF7 PLK1 ACSF2 SLC27A4 CDK12 MVD KIFC2 RAB26 ABCA3 TRANK1 PBK PPIP5K1 CSNK1G1 ARL13B FASN DUS1L CDK1 KIF5B MFN1 CLCN5 KSR2 PIK3CD SLFNL1 RAB33B QARS SPTLC3 SUCLG2 SLFN11 DCAKD GRK2 GMPPB PC ADCY6 DDX23 ATR GK5 ARL10 MYO1D POLE GRAMD1C DALRD3 MCMDC2 P4HTM OPLAH RMI1 FARSA CIITA BBS10 DHTKD1 OGFOD3 AATK ACBD4 IDH2 ERCC6L2 UBA7 GDPGP1 EPHA10 MX2 EP400 TBK1 CHEK2 SRPK3 UBE2G2 LCN12 NRBP2 CDK10 RAD51D ROR1 POMK PIP5K1C KIF18B KIF24 ERCC6L DYNC2H1 FANCM PALM3 RAB42 MAPK12 SBK1 ENTPD8 ACADSB DYNC1H1 RAD54B SHPK RAB40C TXNRD3 ATAD3A MYO1C SPG7 GRK6 RPS6KL1 UCKL1 PAPSS2 ABCA4 CDC42BPB MTOR MAP3K3 BMPR2 SKIV2L DHX16 DDX47 KIF4B PI4KA MICAL3 MARS2 PRKDC FPGT IKBKE STRADA AARSD1 ACACA DHRS11 PIP4K2B MYO19 HSD17B1 ALDH1B1 TM7SF2 ETFDH DHCR7 AGRN FAM114A2 CHST12 HS3ST5 NR1H3 TPX2 CAV1 CYP1B1 CYP4B1 PPOX |
| Down regulated | 8.85E-18 | 396 | Drug binding | MSH2 FOLR1 NKTR GLE1 AARS2 PFKL RECQL4 SLC19A1 SHMT1 GLDC TMEM173 DPYD BLM MSH5 RTEL1 ACSS2 P2RX4 AKT1 UHMK1 TYMS CALCR CDKL5 PPP5C WNK1 PDK3 CYP2D6 PCIF1 DMPK AKT2 ABCA2 PPP3CB BMPR1A PNPO OAS3 STK38 ICK IDE PANK3 LATS1 TOP2A MYH10 ACVR1B MMAB PIF1 TP53 MAP3K6 XPR1 CDC42BPA PPIP5K2 SMARCA5 HLCS RHOBTB3 METTL3 MAP4K2 TAP1 CDC42BPG GRIN1 MYO18A MAP3K5 ENPP1 TAP2 ERCC6 DHFR TSSK4 CFTR DHX33 LIG3 ACSM3 LARS2 DDX11 AQR NLRP2 EHD2 RTEL1-TNFRSF6B DNAH5 YTHDC2 PIK3CB ENTPD2 ATP11B ALDH18A1 ADCK1 ABCA7 PMS1 PKN2 ERBB3 MYLK PRKCQ FGFR2 MYO9A IARS2 ROCK1 FGFR3 COASY ATP11A ATP2B1 RPS6KA2 HLTF PRKACA RPS6KA6 SMC1A NSF MCM6 SLC46A1 ACACB TOP2B UBE2T KIF22 ATP8B1 CAD ATRX RAD54L MAST2 EIF2AK1 AURKA OAS1 KIF16B DHX32 KIF4A XYLB MAP3K1 NUBP2 CDC7 P2RX6 DDX17 TTLL12 DICER1 MTHFD1 KIF3B VWA8 MAPK3 LONP2 DHODH NME3 CLCN7 EEF2K EARS2 BCKDK CSK IKBKB ERCC2 MYH14 TYK2 LIG1 ATP13A1 NOD1 DDX58 SMC3 RPS6KB1 RECQL5 DHX58 ABCC3 MAP2K6 HIPK3 ACCS MVK OAS2 RFC5 FRK MAPK14 MCM3 MDN1 ASCC3 PTK7 CUL9 PRPF4B KIF20A RAD50 SMC4 ABCC5 NEK4 TTL PIKFYVE ACTR1B IFIH1 PASK STK25 FAM20B TTF2 MTR CDK18 DARS2 NEK2 KIF14 ABCG2 NEK9 TTLL5 HELLS APAF1 KIF18A CIT NRK COQ8B CHD6 ABCC10 MCM8 XRCC3 TRAP1 PKMYT1 CHTF18 PEX1 EIF2AK4 MYO5C TEP1 QRSL1 TRPM4 HELZ2 PAK4 UBE4B KIF3A ACLY MCCC2 PRKAA1 FIGNL1 DDC MMACHC PIK3C2B CSNK1G2 DPH6 CMPK2 OASL DYNC1LI2 TTLL4 NEK3 ALPK3 BRIP1 SMC2 DDR1 NARS2 NEK1 DDX60 KIF23 KHK ADCY3 ACTR1A KIF11 KIF20B ENTPD1 DNA2 CENPE CSAD NLRC5 KSR1 AFG3L2 ERBB2 FKBP10 CYP4B1 TARS2 ATP8B2 SLC27A3 ITPKB SNRNP200 ABCA12 DGKQ TBCK DDX46 RARS2 SHPRH EPHA1 TAF1 CAMK2G MKI67 DGKZ ATM HYOU1 DHX37 NUBPL ACSL1 PANK1 LRRK1 ABCA5 OBSCN SLFN13 MOV10 CDK20 UBE2L6 BUB1B FUK MYO1E ATP13A2 STARD9 GNE PKN3 TAOK1 NLRX1 ADCY9 PARS2 SMC6 SMARCAD1 DHX57 HIPK1 STK36 TGFBR2 RYK KIF15 MST1R RAD54L2 FASTK SYK WNK2 ATP7A TK2 HSP90B1 KIF7 PLK1 ACSF2 CDK12 MVD KIFC2 ABCA3 TRANK1 PBK PPIP5K1 CSNK1G1 FASN CDK1 KIF5B CLCN5 KSR2 PIK3CD SLFNL1 QARS SPTLC3 SUCLG2 SLFN11 DCAKD GRK2 PC ADCY6 DDX23 ATR GK5 MYO1D CHID1 DALRD3 MCMDC2 OPLAH FARSA CIITA BBS10 AATK ERCC6L2 UBA7 EPHA10 EP400 TBK1 CHEK2 SRPK3 UBE2G2 NRBP2 CDK10 RAD51D ROR1 POMK PIP5K1C KIF18B KIF24 ERCC6L DYNC2H1 FANCM PALM3 MAPK12 SBK1 ENTPD8 DYNC1H1 RAD54B SHPK ATAD3A MYO1C SPG7 GRK6 RPS6KL1 UCKL1 PAPSS2 ABCA4 CDC42BPB MTOR MAP3K3 BMPR2 SKIV2L DHX16 DDX47 KIF4B PI4KA MARS2 PRKDC IKBKE STRADA AARSD1 ACACA PIP4K2B MYO19 ITPR3 HIF1AN TPX2 CYP1B1 SIGMAR1 |
| Down regulated | 1.09E-17 | 573 | Anion binding | RHOBTB2 SNX1 TUBG2 CPNE3 MSH2 ITPR3 PICK1 GSDMD PLEKHA8 TUBD1 FOLR1 ARL6 SNX17 WDR35 GLE1 ITPR2 PACSIN1 AARS2 RHOT1 TUBG1 SYT11 MMACHC DBT RHOT2 PFKL SNX27 ANXA9 GSDMC SYT8 RECQL4 ACOT11 BBS5 ARFIP1 RHOBTB3 TUB LDHD PLA2G4F CLCN5 SYT12 SLC19A1 SHMT1 MTHFR GLDC D2HGDH TUBB TMEM173 PDE4B BLM SYT15 MSH5 TECPR1 DENND1C SYT3 CPTP RTEL1 ANXA8 ACSS2 P2RX4 AKT1 UHMK1 TYMS ENPP1 KRIT1 ARHGAP44 CDKL5 PPP5C ZFYVE16 RAB27B ARAP2 WNK1 PDK3 HYAL2 SNX13 KIF16B PNPLA3 FOXRED2 SAMHD1 EEA1 DMPK AKT2 OGDH ABCA2 PLEKHA1 BMPR1A GBF1 PNPO PHF12 OAS3 RAB5B C2CD5 STK38 ICK HSD17B8 HMGCR CCDC88A LANCL1 PASK GBP1 IDE PANK3 STEAP4 LATS1 MYH10 SNX14 ACVR1B APPL2 DAB2IP NUMA1 MYOF PIF1 TP53 MAP3K6 XPR1 CDC42BPA PPIP5K2 ASTN2 JPH2 MMAA PANK1 SMARCA5 OBSCN RRAGA MYO1E APPL1 LDLRAP1 RAPGEF6 EPB41 HLCS ATP13A2 ARHGAP35 RACGAP1 WDFY3 KDM1B HIF1AN MAP4K2 TAP1 THBS3 CDC42BPG RAB1B GRAMD2A GRIN1 ADAP2 ANKFY1 RXRA ARAP1 LIPI MYO18A RABL6 NF1 MAP3K5 NUDT16 TAP2 ERCC6 DHFR UGT1A1 UGT1A7 TSSK4 CFTR ARHGAP33 DHX33 LIG3 ACSM3 LARS2 DDX11 AQR NLRP2 GRAMD1B EHD2 RTEL1-TNFRSF6B DNAH5 YTHDC2 PIK3CB ENTPD2 ATP11B ALDH18A1 ADCK1 ABCA7 PMS1 PKN2 ERBB3 MYLK PRKCQ FGFR2 MYO9A IARS2 ROCK1 FGFR3 COASY ATP11A ATP2B1 RPS6KA2 HLTF PRKACA RPS6KA6 SMC1A HMMR NSF PLD1 TUBA3D MCM6 SLC46A1 ACACB TOP2B UBE2T SLC1A3 KIF22 RABL2B ATP8B1 HADHA CAD ATRX RAD54L MAST2 EIF2AK1 AURKA OAS1 DHX32 KIF4A DLD CCDC80 XYLB MAP3K1 NUBP2 CDC7 P2RX6 DDX17 RAB36 TTLL12 IFT27 NIN DICER1 MTHFD1 KIF3B VWA8 MAPK3 LONP2 DHODH NME3 CLCN7 EEF2K EARS2 BCKDK CSK IKBKB SQLE ERCC2 ILVBL APLP1 MYH14 TYK2 PTPRS LIG1 RAB3D ATP13A1 NOD1 SLC1A1 DNM1 DDX58 SMC3 RPS6KB1 RECQL5 DHX58 ABCC3 MAP2K6 HIPK3 ACCS MVK ACAD10 OAS2 RFC5 FRK MAPK14 MCM3 MDN1 RAB23 ASCC3 PTK7 CUL9 PRPF4B KIF20A RAD50 SMC4 PRKAR2A ABCC5 NEK4 RTKN TTL PIKFYVE ACTR1B DNAJC27 IFIH1 SPTBN1 FN1 STK25 DHCR24 FAM20B TTF2 ACADM CDK18 P3H1 DARS2 NEK2 KIF14 ABCG2 NEK9 TTLL5 COQ6 ATL2 HELLS SNX19 APAF1 KIF18A CIT ACADS NRK COQ8B CHD6 ABCC10 MCM8 XRCC3 TRAP1 PKMYT1 CHTF18 PEX1 EIF2AK4 MYO5C IVD TEP1 GTPBP3 QRSL1 TRPM4 HELZ2 PAK4 UBE4B KIF3A ACLY TOP2A MCCC2 PRKAA1 EEFSEC FIGNL1 DDC PIK3C2B CSNK1G2 DPH6 CMPK2 OASL DYNC1LI2 TTLL4 NEK3 ALPK3 BRIP1 SMC2 TLN1 DDR1 NARS2 NEK1 DDX60 KIF23 KHK ADCY3 ACTR1A KIF11 KIF20B ENTPD1 DNA2 CENPE MMAB CSAD RAB15 NLRC5 KSR1 AFG3L2 ERBB2 PTPRF TARS2 ATP8B2 S100A8 SLC27A3 HCN3 ITPKB SNRNP200 RABL2A FBLN7 ABCA12 NCEH1 DGKQ TBCK DDX46 RARS2 SHPRH EPHA1 RAB19 TAF1 CAMK2G MKI67 MTG1 DGKZ ATM HYOU1 DOC2A DHX37 NUBPL ACAD8 ACSL1 LRRK1 ABCA5 SLFN13 MOV10 ADAMTS3 CDK20 UBE2L6 AIFM1 BUB1B FUK STARD9 GNE PKN3 TAOK1 NLRX1 ADCY9 PARS2 GBP4 SMC6 SMARCAD1 DHX57 HIPK1 FSTL1 STK36 TGFBR2 RYK KIF15 MST1R RAD54L2 FASTK SYK WNK2 ATP7A TK2 HSP90B1 KIF7 PLK1 ACSF2 CDK12 MVD KIFC2 RAB26 ABCA3 TRANK1 PBK PPIP5K1 CSNK1G1 ARL13B FASN DUS1L CDK1 KIF5B MFN1 KSR2 PIK3CD SLFNL1 RAB33B QARS SPTLC3 SUCLG2 SLFN11 DCAKD GRK2 GMPPB SNX33 PC ADCY6 DDX23 ATR GK5 ARL10 MYO1D DALRD3 MCMDC2 P4HTM OPLAH FARSA CIITA BBS10 DHTKD1 OGFOD3 AATK ACBD4 ERCC6L2 UBA7 EPHA10 MX2 EP400 TBK1 CHEK2 SRPK3 UBE2G2 NRBP2 CDK10 RAD51D ROR1 POMK RTN4RL1 PIP5K1C KIF18B THBS2 PCLO KIF24 ERCC6L DYNC2H1 FANCM PALM3 RAB42 MAPK12 SBK1 ENTPD8 ACADSB DYNC1H1 RAD54B SHPK RAB40C TXNRD3 ATAD3A MYO1C SPG7 GRK6 RPS6KL1 UCKL1 PAPSS2 ABCA4 UNC13B CDC42BPB MTOR MAP3K3 BMPR2 SKIV2L DHX16 DDX47 KIF4B PI4KA MICAL3 MARS2 PRKDC FPGT IKBKE STRADA AARSD1 ACACA PIP4K2B MYO19 PIGU HSD17B1 CHMP3 SYTL2 PARP9 CXCL10 GPAA1 SCIN SEMA5A PTGES2 TMEM184A ETFDH LCN12 AGRN DPYD CHST12 CHST14 HS3ST5 TPX2 LRP1 FGFBP1 PIGK PPOX |
| Down regulated | 1.39E-17 | 496 | Transferase activity | BIRC3 TFB1M PIAS1 GYG2 PRKCQ HLTF AURKA XYLB MAP3K1 SUV39H1 IKBKB GYS1 TYK2 BIRC2 FRK KMT2A PIGZ PIAS3 ACVR1B AKT1 SHPRH TAF1 RNF144A CPT2 AGL SYK PLK1 TMEM129 UBA7 MTOR PRMT6 MRM1 ANKIB1 HS3ST1 ACSM3 CROT PIGQ BRCA1 POLR3B PHKA2 PIK3CB TARBP1 WNK1 PIGV POLD1 PKN2 ERBB3 MYLK FGFR2 FGFR3 COASY POLR1A NEDD4 PIGB NDST1 RPS6KA2 RPS6KA6 UBE2T FDFT1 KAT6A CAD HACE1 POMGNT1 MAST2 EIF2AK1 HUWE1 ALG9 ALG6 ANAPC5 OAS1 CDC7 POLRMT ZDHHC8 RNF215 PPIL2 MCAT POLR3H POLE2 RABGGTA MAGT1 MAPK3 PHKB NME3 SETD6 QPRT ERCC2 DOT1L DMPK AKT2 LFNG CCNJ BMPR1A FBXL15 RPS6KB1 MAP2K6 NSD2 CCND1 NAA40 MVK OAS3 OAS2 GNPTAB MAPK14 STK38 ICK FBXL4 PRPF4B CCNG1 RAD50 NEK4 PASK STK25 BIRC6 FAM20B GNPAT MTR CDK18 NEK2 FBXL5 TRIM32 GPAM ACAT2 TRMT1L NRK LPGAT1 COQ8B LRRC29 PKMYT1 METTL16 CASD1 MGAT3 EIF2AK4 ASH2L COLGALT1 PAK4 DNMT1 UBE4B LATS1 ACLY PRKAA1 PRMT7 PIK3C2B CSNK1G2 MEN1 CCNB1 CMPK2 KDELC1 CCNJL FBXL8 UGGT1 ST6GALNAC4 CMTR1 DDR1 NEK1 DBT SELENOI KHK HERC5 CHST4 ERBB2 PFKL MAP3K6 PARP1 UBR3 DGKQ CCNA2 SKP2 PPIP5K2 FBXL17 RMND5B EPHA1 CAMK2G DGKZ ATM AASDHPPT KAT14 B3GAT3 UHMK1 ATG10 ANAPC1 HS2ST1 CARNMT1 DPY19L4 CDK20 UBE2L6 ZDHHC5 KAT6B BUB1B ST3GAL2 FUK CCNB2 ZDHHC1 AGPAT3 ZDHHC12 PKN3 TAOK1 FDPS CCNF HENMT1 POGLUT1 TGFBR2 RNF123 MST1R PRMT9 HGSNAT WNK2 TRMT10B PIGO COMTD1 NSD1 POLL TK2 NNMT UGT1A6 RNF214 CDK12 KMT2D MAP4K2 PCMTD1 PPIP5K1 CSNK1G1 CPT1C CDK1 POLH C1GALT1C1 ZDHHC16 PIK3CD DPAGT1 FUT10 LCLAT1 DCAKD DPY19L1 ZDHHC24 CTU2 ATR GK5 ZDHHC21 B3GALT6 FUT2 SHMT1 POLE KDELC2 NSUN3 DPM3 B3GNT3 RNF135 TTC3 ALG12 FANCF CHST6 GDPGP1 EPHA10 TBK1 CHEK2 ZDHHC23 SRPK3 UBE2G2 DGAT1 NRBP2 CDK10 POMK TAF9B B3GLCT MAPK12 SBK1 NHLRC3 SHPK MAP3K5 GSTK1 PIGN PRIM1 UCKL1 PAPSS2 LTN1 MAP3K3 DZIP3 PJA2 PCMTD2 BMPR2 AGPAT1 B3GALT4 QTRT1 GTF2H4 NEURL1B NEURL4 POLR2J2 UGT1A1 PI4KA UGT1A7 HS3ST5 PRKDC FDXACB1 HMBS FNTB IKBKE STRADA PIGW TSSK4 MYCBP2 CDKL5 EXTL3 XYLT2 B4GALT7 RC3H2 ALDH18A1 ROCK1 PDK3 ST6GALNAC2 PRKACA CPNE3 PNPLA3 PCIF1 POFUT1 POLA1 PARP4 MGRN1 EEF2K UBR5 TRIM37 HIPK3 EFEMP1 ASH1L EEF2KMT PANK3 EEF1AKMT3 HECTD3 HERC2 TEP1 TRAF7 SETDB2 CHST12 BARD1 PARP9 MMAB SETDB1 GALNT2 ITPKB CDC42BPA CSGALNACT1 DLAT EXT2 MCM3AP EXTL2 DTX3L PDSS2 GALNT10 FASTK METTL3 HRASLS5 CHST14 IRF2BP1 GSTA4 CDC42BPG SPTLC3 PARP14 RNF26 B4GAT1 ALG10B TYMS GALNT11 PARP10 EHMT1 B4GALNT4 TRIM69 ZDHHC17 FBXL22 GRK6 KLHL9 CDC42BPB EEF1AKMT2 EHMT2 RNF208 PIP4K2B MED24 REV3L UBR7 POLA2 GLT8D1 ALG1 JADE2 ADCK1 GBA2 MGAT4A NCOA1 HADHA NAT14 TRMT2A TAB1 CDS2 POLI FAM173A BCKDK CSK HERC1 ILVBL FKTN MED31 EZH1 PPP1R9B DPH1 GALNT7 SEPSECS DTX4 LPCAT3 PTK7 CUL9 PRKAR2A HEMK1 PIKFYVE LANCL1 ATF2 ICMT FASTKD2 AKAP7 NEK9 RNF170 GTDC1 CIT PACSIN1 MED20 TMTC4 MED1 AKAP9 OSGEPL1 POMT1 TRIM22 COQ3 CLOCK STT3A OASL SMYD5 NEK3 ALPK3 FASTKD1 HERC6 ALG10 TMTC3 GIT2 GALNT6 FTO KSR1 PIGM TAMM41 GMPPA TBCK TRIM41 CDKN2B FAM57B PANK1 LRRK1 OBSCN GNE CALM3 TRIM58 HIPK1 STK36 WDFY3 RYK METTL17 GGT6 PBK FASN TRIM56 PRDM10 AKAP13 KSR2 MBOAT1 GRK2 HECTD4 ADCK5 GMPPB PTDSS2 ABO B3GNTL1 CIITA AATK MED12 ROR1 PCYT2 PIP5K1C PTAR1 ALG1L TRRAP RPS6KL1 TRIM59 FPGT FAM98A TSPAN17 POLD3 FBXO21 ANAPC4 CCL2 EFNB3 PIK3R3 CRIM1 PIGF |
| Down regulated | 1.02E-16 | 421 | Purine nucleotide binding | RHOBTB2 TUBG2 MSH2 POLA1 TUBD1 ARL6 SIL1 AARS2 RHOT1 TUBG1 RHOT2 PFKL RECQL4 ACOT11 RHOBTB3 TUBB TMEM173 PDE4B BLM MSH5 RTEL1 ACSS2 P2RX4 AKT1 UHMK1 ENPP1 CDKL5 PPP5C RAB27B WNK1 PDK3 PNPLA3 SAMHD1 DMPK AKT2 ABCA2 BMPR1A OAS3 RAB5B STK38 ICK GBP1 IDE PANK3 LATS1 MYH10 ACVR1B PIF1 TP53 MAP3K6 CDC42BPA PPIP5K2 MMAA PANK1 SMARCA5 RRAGA MAP4K2 TAP1 CDC42BPG RAB1B MYO18A RABL6 MAP3K5 NUDT16 TAP2 ERCC6 TSSK4 CFTR DHX33 LIG3 ACSM3 LARS2 DDX11 AQR NLRP2 EHD2 RTEL1-TNFRSF6B DNAH5 YTHDC2 PIK3CB ENTPD2 ATP11B ALDH18A1 ADCK1 ABCA7 PMS1 PKN2 ERBB3 MYLK PRKCQ FGFR2 MYO9A IARS2 ROCK1 FGFR3 COASY ATP11A ATP2B1 RPS6KA2 HLTF PRKACA RPS6KA6 SMC1A NSF TUBA3D MCM6 ACACB TOP2B UBE2T KIF22 RABL2B ATP8B1 HADHA CAD ATRX RAD54L MAST2 EIF2AK1 AURKA OAS1 KIF16B DHX32 KIF4A XYLB MAP3K1 NUBP2 CDC7 P2RX6 DDX17 RAB36 TTLL12 IFT27 NIN DICER1 MTHFD1 KIF3B VWA8 MAPK3 LONP2 NME3 CLCN7 EEF2K EARS2 BCKDK CSK IKBKB ERCC2 MYH14 TYK2 LIG1 RAB3D ATP13A1 NOD1 DNM1 DDX58 SMC3 RPS6KB1 RECQL5 DHX58 ABCC3 MAP2K6 HIPK3 MVK OAS2 RFC5 FRK MAPK14 MCM3 MDN1 RAB23 ASCC3 PTK7 CUL9 PRPF4B KIF20A RAD50 SMC4 PRKAR2A ABCC5 NEK4 RTKN TTL PIKFYVE ACTR1B DNAJC27 IFIH1 PASK STK25 FAM20B TTF2 CDK18 DARS2 NEK2 KIF14 ABCG2 NEK9 TTLL5 ATL2 HELLS APAF1 KIF18A CIT NRK COQ8B CHD6 ABCC10 MCM8 XRCC3 TRAP1 PKMYT1 CHTF18 PEX1 EIF2AK4 MYO5C TEP1 GTPBP3 QRSL1 TRPM4 HELZ2 PAK4 UBE4B KIF3A ACLY TOP2A MCCC2 PRKAA1 EEFSEC FIGNL1 PIK3C2B CSNK1G2 DPH6 CMPK2 OASL DYNC1LI2 TTLL4 NEK3 ALPK3 BRIP1 SMC2 DDR1 NARS2 NEK1 DDX60 KIF23 KHK ADCY3 ACTR1A KIF11 KIF20B ENTPD1 DNA2 CENPE MMAB RAB15 NLRC5 KSR1 AFG3L2 ERBB2 TARS2 ATP8B2 SLC27A3 HCN3 ITPKB SNRNP200 RABL2A ABCA12 DGKQ TBCK DDX46 RARS2 SHPRH EPHA1 RAB19 TAF1 CAMK2G MKI67 MTG1 DGKZ ATM HYOU1 DHX37 NUBPL ACSL1 LRRK1 ABCA5 OBSCN SLFN13 MOV10 CDK20 UBE2L6 BUB1B FUK MYO1E HLCS ATP13A2 STARD9 GNE ARHGAP35 PKN3 TAOK1 NLRX1 ADCY9 PARS2 GBP4 SMC6 SMARCAD1 DHX57 HIPK1 STK36 TGFBR2 RYK KIF15 MST1R RAD54L2 FASTK SYK WNK2 ATP7A TK2 HSP90B1 KIF7 PLK1 ACSF2 CDK12 MVD KIFC2 RAB26 ABCA3 TRANK1 PBK PPIP5K1 CSNK1G1 ARL13B CDK1 KIF5B MFN1 CLCN5 KSR2 PIK3CD SLFNL1 RAB33B QARS SUCLG2 SLFN11 DCAKD GRK2 GMPPB PC ADCY6 DDX23 ATR GK5 ARL10 MYO1D DALRD3 MCMDC2 OPLAH FARSA CIITA BBS10 AATK ACBD4 ERCC6L2 UBA7 EPHA10 MX2 EP400 TBK1 CHEK2 SRPK3 UBE2G2 NRBP2 CDK10 RAD51D ROR1 POMK PIP5K1C KIF18B KIF24 ERCC6L DYNC2H1 FANCM PALM3 RAB42 MAPK12 SBK1 ENTPD8 DYNC1H1 RAD54B SHPK RAB40C ATAD3A MYO1C SPG7 GRK6 RPS6KL1 UCKL1 PAPSS2 ABCA4 CDC42BPB MTOR MAP3K3 BMPR2 SKIV2L DHX16 DDX47 KIF4B PI4KA MARS2 PRKDC FPGT IKBKE STRADA AARSD1 ACACA PIP4K2B MYO19 FAM114A2 CHST12 HS3ST5 TPX2 |
| Down regulated | 1.17E-16 | 408 | Purine ribonucleoside triphosphate binding | RHOBTB2 TUBG2 MSH2 TUBD1 ARL6 AARS2 RHOT1 TUBG1 RHOT2 PFKL RECQL4 RHOBTB3 TUBB BLM MSH5 RTEL1 P2RX4 AKT1 UHMK1 CDKL5 PPP5C RAB27B WNK1 PDK3 DMPK AKT2 ABCA2 BMPR1A OAS3 STK38 ICK GBP1 IDE PANK3 LATS1 MYH10 ACVR1B PIF1 TP53 MAP3K6 CDC42BPA PPIP5K2 MMAA SMARCA5 RRAGA MAP4K2 TAP1 CDC42BPG RAB1B MYO18A RABL6 MAP3K5 ENPP1 NUDT16 TAP2 ERCC6 TSSK4 CFTR DHX33 LIG3 ACSM3 LARS2 DDX11 AQR NLRP2 EHD2 RTEL1-TNFRSF6B DNAH5 YTHDC2 PIK3CB ENTPD2 ATP11B ALDH18A1 ADCK1 ABCA7 PMS1 PKN2 ERBB3 MYLK PRKCQ FGFR2 MYO9A IARS2 ROCK1 FGFR3 COASY ATP11A ATP2B1 RPS6KA2 HLTF PRKACA RPS6KA6 SMC1A NSF TUBA3D MCM6 ACACB TOP2B UBE2T KIF22 RABL2B ATP8B1 CAD ATRX RAD54L MAST2 EIF2AK1 AURKA OAS1 KIF16B DHX32 KIF4A XYLB MAP3K1 NUBP2 CDC7 P2RX6 DDX17 RAB36 TTLL12 IFT27 NIN DICER1 MTHFD1 SAMHD1 KIF3B VWA8 MAPK3 LONP2 NME3 CLCN7 EEF2K EARS2 BCKDK CSK IKBKB ERCC2 MYH14 TYK2 LIG1 RAB3D ATP13A1 NOD1 DNM1 DDX58 SMC3 RPS6KB1 RECQL5 DHX58 ABCC3 MAP2K6 HIPK3 MVK OAS2 RFC5 RAB5B FRK MAPK14 MCM3 MDN1 RAB23 ASCC3 PTK7 CUL9 PRPF4B KIF20A RAD50 SMC4 ABCC5 NEK4 RTKN TTL PIKFYVE ACTR1B DNAJC27 IFIH1 PASK STK25 FAM20B TTF2 CDK18 DARS2 NEK2 KIF14 ABCG2 NEK9 TTLL5 ATL2 HELLS APAF1 KIF18A CIT NRK COQ8B CHD6 ABCC10 MCM8 XRCC3 TRAP1 PKMYT1 CHTF18 PEX1 EIF2AK4 MYO5C TEP1 GTPBP3 QRSL1 TRPM4 HELZ2 PAK4 UBE4B ACSS2 KIF3A ACLY TOP2A MCCC2 PRKAA1 EEFSEC FIGNL1 PIK3C2B CSNK1G2 DPH6 CMPK2 OASL DYNC1LI2 TTLL4 NEK3 ALPK3 BRIP1 SMC2 DDR1 NARS2 NEK1 DDX60 KIF23 KHK ADCY3 ACTR1A KIF11 KIF20B ENTPD1 DNA2 CENPE MMAB RAB15 NLRC5 KSR1 AFG3L2 ERBB2 TARS2 ATP8B2 SLC27A3 ITPKB SNRNP200 RABL2A ABCA12 DGKQ TBCK DDX46 RARS2 SHPRH EPHA1 RAB19 TAF1 CAMK2G MKI67 MTG1 DGKZ ATM HYOU1 DHX37 NUBPL ACSL1 PANK1 LRRK1 ABCA5 OBSCN SLFN13 MOV10 CDK20 UBE2L6 BUB1B FUK MYO1E HLCS ATP13A2 STARD9 GNE ARHGAP35 PKN3 TAOK1 NLRX1 ADCY9 PARS2 GBP4 SMC6 SMARCAD1 DHX57 HIPK1 STK36 TGFBR2 RYK KIF15 MST1R RAD54L2 FASTK SYK WNK2 ATP7A TK2 HSP90B1 KIF7 PLK1 ACSF2 CDK12 MVD KIFC2 RAB26 ABCA3 TRANK1 PBK PPIP5K1 CSNK1G1 ARL13B CDK1 KIF5B MFN1 CLCN5 KSR2 PIK3CD SLFNL1 RAB33B QARS SUCLG2 SLFN11 DCAKD GRK2 GMPPB PC ADCY6 DDX23 ATR GK5 ARL10 MYO1D DALRD3 MCMDC2 OPLAH FARSA CIITA BBS10 AATK ERCC6L2 UBA7 EPHA10 MX2 EP400 TBK1 CHEK2 SRPK3 UBE2G2 NRBP2 CDK10 RAD51D ROR1 POMK PIP5K1C KIF18B KIF24 ERCC6L DYNC2H1 FANCM PALM3 RAB42 MAPK12 SBK1 ENTPD8 DYNC1H1 RAD54B SHPK RAB40C ATAD3A MYO1C SPG7 GRK6 RPS6KL1 UCKL1 PAPSS2 ABCA4 CDC42BPB MTOR MAP3K3 BMPR2 SKIV2L DHX16 DDX47 KIF4B PI4KA MARS2 PRKDC FPGT IKBKE STRADA AARSD1 ACACA PIP4K2B MYO19 TPX2 |
| Down regulated | 1.39E-16 | 418 | Purine ribonucleotide binding | RHOBTB2 TUBG2 MSH2 TUBD1 ARL6 AARS2 RHOT1 TUBG1 RHOT2 PFKL RECQL4 ACOT11 RHOBTB3 TUBB TMEM173 PDE4B BLM MSH5 RTEL1 ACSS2 P2RX4 AKT1 UHMK1 ENPP1 CDKL5 PPP5C RAB27B WNK1 PDK3 PNPLA3 DMPK AKT2 ABCA2 BMPR1A OAS3 RAB5B STK38 ICK GBP1 IDE PANK3 LATS1 MYH10 ACVR1B PIF1 TP53 MAP3K6 CDC42BPA PPIP5K2 MMAA PANK1 SMARCA5 RRAGA MAP4K2 TAP1 CDC42BPG RAB1B MYO18A RABL6 MAP3K5 NUDT16 TAP2 ERCC6 TSSK4 CFTR DHX33 LIG3 ACSM3 LARS2 DDX11 AQR NLRP2 EHD2 RTEL1-TNFRSF6B DNAH5 YTHDC2 PIK3CB ENTPD2 ATP11B ALDH18A1 ADCK1 ABCA7 PMS1 PKN2 ERBB3 MYLK PRKCQ FGFR2 MYO9A IARS2 ROCK1 FGFR3 COASY ATP11A ATP2B1 RPS6KA2 HLTF PRKACA RPS6KA6 SMC1A NSF TUBA3D MCM6 ACACB TOP2B UBE2T KIF22 RABL2B ATP8B1 HADHA CAD ATRX RAD54L MAST2 EIF2AK1 AURKA OAS1 KIF16B DHX32 KIF4A XYLB MAP3K1 NUBP2 CDC7 P2RX6 DDX17 RAB36 TTLL12 IFT27 NIN DICER1 MTHFD1 SAMHD1 KIF3B VWA8 MAPK3 LONP2 NME3 CLCN7 EEF2K EARS2 BCKDK CSK IKBKB ERCC2 MYH14 TYK2 LIG1 RAB3D ATP13A1 NOD1 DNM1 DDX58 SMC3 RPS6KB1 RECQL5 DHX58 ABCC3 MAP2K6 HIPK3 MVK OAS2 RFC5 FRK MAPK14 MCM3 MDN1 RAB23 ASCC3 PTK7 CUL9 PRPF4B KIF20A RAD50 SMC4 PRKAR2A ABCC5 NEK4 RTKN TTL PIKFYVE ACTR1B DNAJC27 IFIH1 PASK STK25 FAM20B TTF2 CDK18 DARS2 NEK2 KIF14 ABCG2 NEK9 TTLL5 ATL2 HELLS APAF1 KIF18A CIT NRK COQ8B CHD6 ABCC10 MCM8 XRCC3 TRAP1 PKMYT1 CHTF18 PEX1 EIF2AK4 MYO5C TEP1 GTPBP3 QRSL1 TRPM4 HELZ2 PAK4 UBE4B KIF3A ACLY TOP2A MCCC2 PRKAA1 EEFSEC FIGNL1 PIK3C2B CSNK1G2 DPH6 CMPK2 OASL DYNC1LI2 TTLL4 NEK3 ALPK3 BRIP1 SMC2 DDR1 NARS2 NEK1 DDX60 KIF23 KHK ADCY3 ACTR1A KIF11 KIF20B ENTPD1 DNA2 CENPE MMAB RAB15 NLRC5 KSR1 AFG3L2 ERBB2 TARS2 ATP8B2 SLC27A3 HCN3 ITPKB SNRNP200 RABL2A ABCA12 DGKQ TBCK DDX46 RARS2 SHPRH EPHA1 RAB19 TAF1 CAMK2G MKI67 MTG1 DGKZ ATM HYOU1 DHX37 NUBPL ACSL1 LRRK1 ABCA5 OBSCN SLFN13 MOV10 CDK20 UBE2L6 BUB1B FUK MYO1E HLCS ATP13A2 STARD9 GNE ARHGAP35 PKN3 TAOK1 NLRX1 ADCY9 PARS2 GBP4 SMC6 SMARCAD1 DHX57 HIPK1 STK36 TGFBR2 RYK KIF15 MST1R RAD54L2 FASTK SYK WNK2 ATP7A TK2 HSP90B1 KIF7 PLK1 ACSF2 CDK12 MVD KIFC2 RAB26 ABCA3 TRANK1 PBK PPIP5K1 CSNK1G1 ARL13B CDK1 KIF5B MFN1 CLCN5 KSR2 PIK3CD SLFNL1 RAB33B QARS SUCLG2 SLFN11 DCAKD GRK2 GMPPB PC ADCY6 DDX23 ATR GK5 ARL10 MYO1D DALRD3 MCMDC2 OPLAH FARSA CIITA BBS10 AATK ACBD4 ERCC6L2 UBA7 EPHA10 MX2 EP400 TBK1 CHEK2 SRPK3 UBE2G2 NRBP2 CDK10 RAD51D ROR1 POMK PIP5K1C KIF18B KIF24 ERCC6L DYNC2H1 FANCM PALM3 RAB42 MAPK12 SBK1 ENTPD8 DYNC1H1 RAD54B SHPK RAB40C ATAD3A MYO1C SPG7 GRK6 RPS6KL1 UCKL1 PAPSS2 ABCA4 CDC42BPB MTOR MAP3K3 BMPR2 SKIV2L DHX16 DDX47 KIF4B PI4KA MARS2 PRKDC FPGT IKBKE STRADA AARSD1 ACACA PIP4K2B MYO19 CHST12 HS3ST5 TPX2 |
| Down regulated | 1.60E-16 | 420 | Ribonucleotide binding | RHOBTB2 TUBG2 MSH2 TUBD1 ARL6 AARS2 RHOT1 TUBG1 RHOT2 PFKL RECQL4 ACOT11 RHOBTB3 TUBB TMEM173 PDE4B BLM MSH5 RTEL1 ACSS2 P2RX4 AKT1 UHMK1 ENPP1 CDKL5 PPP5C RAB27B WNK1 PDK3 PNPLA3 DMPK AKT2 ABCA2 BMPR1A PNPO OAS3 RAB5B STK38 ICK GBP1 IDE PANK3 LATS1 MYH10 ACVR1B PIF1 TP53 MAP3K6 CDC42BPA PPIP5K2 MMAA PANK1 SMARCA5 RRAGA MAP4K2 TAP1 CDC42BPG RAB1B MYO18A RABL6 MAP3K5 NUDT16 TAP2 ERCC6 TSSK4 CFTR DHX33 LIG3 ACSM3 LARS2 DDX11 AQR NLRP2 EHD2 RTEL1-TNFRSF6B DNAH5 YTHDC2 PIK3CB ENTPD2 ATP11B ALDH18A1 ADCK1 ABCA7 PMS1 PKN2 ERBB3 MYLK PRKCQ FGFR2 MYO9A IARS2 ROCK1 FGFR3 COASY ATP11A ATP2B1 RPS6KA2 HLTF PRKACA RPS6KA6 SMC1A NSF TUBA3D MCM6 ACACB TOP2B UBE2T KIF22 RABL2B ATP8B1 HADHA CAD ATRX RAD54L MAST2 EIF2AK1 AURKA OAS1 KIF16B DHX32 KIF4A XYLB MAP3K1 NUBP2 CDC7 P2RX6 DDX17 RAB36 TTLL12 IFT27 NIN DICER1 MTHFD1 SAMHD1 KIF3B VWA8 MAPK3 LONP2 DHODH NME3 CLCN7 EEF2K EARS2 BCKDK CSK IKBKB ERCC2 MYH14 TYK2 LIG1 RAB3D ATP13A1 NOD1 DNM1 DDX58 SMC3 RPS6KB1 RECQL5 DHX58 ABCC3 MAP2K6 HIPK3 MVK OAS2 RFC5 FRK MAPK14 MCM3 MDN1 RAB23 ASCC3 PTK7 CUL9 PRPF4B KIF20A RAD50 SMC4 PRKAR2A ABCC5 NEK4 RTKN TTL PIKFYVE ACTR1B DNAJC27 IFIH1 PASK STK25 FAM20B TTF2 CDK18 DARS2 NEK2 KIF14 ABCG2 NEK9 TTLL5 ATL2 HELLS APAF1 KIF18A CIT NRK COQ8B CHD6 ABCC10 MCM8 XRCC3 TRAP1 PKMYT1 CHTF18 PEX1 EIF2AK4 MYO5C TEP1 GTPBP3 QRSL1 TRPM4 HELZ2 PAK4 UBE4B KIF3A ACLY TOP2A MCCC2 PRKAA1 EEFSEC FIGNL1 PIK3C2B CSNK1G2 DPH6 CMPK2 OASL DYNC1LI2 TTLL4 NEK3 ALPK3 BRIP1 SMC2 DDR1 NARS2 NEK1 DDX60 KIF23 KHK ADCY3 ACTR1A KIF11 KIF20B ENTPD1 DNA2 CENPE MMAB RAB15 NLRC5 KSR1 AFG3L2 ERBB2 TARS2 ATP8B2 SLC27A3 HCN3 ITPKB SNRNP200 RABL2A ABCA12 DGKQ TBCK DDX46 RARS2 SHPRH EPHA1 RAB19 TAF1 CAMK2G MKI67 MTG1 DGKZ ATM HYOU1 DHX37 NUBPL ACSL1 LRRK1 ABCA5 OBSCN SLFN13 MOV10 CDK20 UBE2L6 BUB1B FUK MYO1E HLCS ATP13A2 STARD9 GNE ARHGAP35 PKN3 TAOK1 NLRX1 ADCY9 PARS2 GBP4 SMC6 SMARCAD1 DHX57 HIPK1 STK36 TGFBR2 RYK KIF15 MST1R RAD54L2 FASTK SYK WNK2 ATP7A TK2 HSP90B1 KIF7 PLK1 ACSF2 CDK12 MVD KIFC2 RAB26 ABCA3 TRANK1 PBK PPIP5K1 CSNK1G1 ARL13B CDK1 KIF5B MFN1 CLCN5 KSR2 PIK3CD SLFNL1 RAB33B QARS SUCLG2 SLFN11 DCAKD GRK2 GMPPB PC ADCY6 DDX23 ATR GK5 ARL10 MYO1D DALRD3 MCMDC2 OPLAH FARSA CIITA BBS10 AATK ACBD4 ERCC6L2 UBA7 EPHA10 MX2 EP400 TBK1 CHEK2 SRPK3 UBE2G2 NRBP2 CDK10 RAD51D ROR1 POMK PIP5K1C KIF18B KIF24 ERCC6L DYNC2H1 FANCM PALM3 RAB42 MAPK12 SBK1 ENTPD8 DYNC1H1 RAD54B SHPK RAB40C ATAD3A MYO1C SPG7 GRK6 RPS6KL1 UCKL1 PAPSS2 ABCA4 CDC42BPB MTOR MAP3K3 BMPR2 SKIV2L DHX16 DDX47 KIF4B PI4KA MARS2 PRKDC FPGT IKBKE STRADA AARSD1 ACACA PIP4K2B MYO19 CHST12 HS3ST5 TPX2 |
| Down regulated | 7.37E-13 | 455 | Carbohydrate derivative binding | RHOBTB2 TUBG2 MSH2 TUBD1 ARL6 AARS2 RHOT1 TUBG1 RHOT2 PFKL RECQL4 ACOT11 PGLYRP4 RHOBTB3 TUBB TMEM173 PDE4B BLM MSH5 RTEL1 ACSS2 P2RX4 AKT1 UHMK1 ENPP1 CDKL5 PPP5C RAB27B WNK1 PDK3 HYAL2 PNPLA3 SAMHD1 DMPK AKT2 PLEKHA8 ENG ABCA2 BMPR1A PNPO OAS3 RAB5B STK38 ICK DROSHA FN1 GBP1 IDE PANK3 LATS1 MYH10 ACVR1B PIF1 TP53 MAP3K6 CDC42BPA PPIP5K2 MMAA PANK1 SMARCA5 RRAGA TGFBR2 MAP4K2 TAP1 THBS3 CDC42BPG RAB1B LIPI MYO18A RABL6 MAP3K5 NUDT16 TAP2 ERCC6 TSSK4 CFTR DHX33 LIG3 ACSM3 LARS2 POLR3B DDX11 AQR NLRP2 EHD2 RTEL1-TNFRSF6B DNAH5 YTHDC2 PIK3CB ENTPD2 ATP11B ALDH18A1 ADCK1 ABCA7 PMS1 PKN2 ERBB3 MYLK PRKCQ FGFR2 MYO9A IARS2 ROCK1 FGFR3 COASY ATP11A ATP2B1 RPS6KA2 HLTF PRKACA RPS6KA6 SMC1A HMMR NSF TUBA3D MCM6 ACACB TOP2B UBE2T KIF22 RABL2B ATP8B1 HADHA CAD ATRX RAD54L MAST2 EIF2AK1 AURKA OAS1 KIF16B DHX32 KIF4A CCDC80 XYLB MAP3K1 NUBP2 CDC7 P2RX6 DDX17 RAB36 TTLL12 IFT27 NIN DICER1 MTHFD1 KIF3B POLA1 VWA8 MAPK3 LONP2 DHODH NME3 CLCN7 EEF2K EARS2 BCKDK CSK IKBKB ERCC2 APLP1 MYH14 TYK2 PTPRS LIG1 RAB3D ATP13A1 NOD1 DNM1 DDX58 SMC3 RPS6KB1 RECQL5 DHX58 ABCC3 MAP2K6 HIPK3 MVK OAS2 RFC5 FRK MAPK14 MCM3 MDN1 RAB23 ASCC3 PTK7 CUL9 PRPF4B KIF20A RAD50 SMC4 PRKAR2A ABCC5 NEK4 RTKN TTL PIKFYVE ACTR1B DNAJC27 IFIH1 PASK STK25 FAM20B TTF2 CDK18 DARS2 NEK2 KIF14 ABCG2 NEK9 TTLL5 ATL2 HELLS APAF1 KIF18A CIT NRK COQ8B CHD6 ABCC10 MCM8 XRCC3 TRAP1 PKMYT1 CHTF18 PEX1 EIF2AK4 MYO5C TEP1 GTPBP3 QRSL1 TRPM4 HELZ2 PAK4 UBE4B KIF3A ACLY TOP2A MCCC2 PRKAA1 EEFSEC FIGNL1 PIK3C2B CSNK1G2 DPH6 CMPK2 OASL DYNC1LI2 TTLL4 NEK3 ALPK3 BRIP1 SMC2 DDR1 NARS2 NEK1 DDX60 KIF23 KHK ADCY3 ACTR1A KIF11 KIF20B ENTPD1 DNA2 CENPE MMAB RAB15 NLRC5 KSR1 AFG3L2 ERBB2 PTPRF TARS2 ATP8B2 SLC27A3 HCN3 ITPKB SNRNP200 RABL2A FBLN7 ABCA12 DGKQ TBCK DDX46 RARS2 SHPRH EPHA1 RAB19 TAF1 CAMK2G MKI67 MTG1 DGKZ ATM HYOU1 DHX37 NUBPL ACSL1 LRRK1 ABCA5 OBSCN SLFN13 MOV10 ADAMTS3 CDK20 UBE2L6 BUB1B FUK MYO1E HLCS ATP13A2 STARD9 GNE ARHGAP35 PKN3 TAOK1 NLRX1 ADCY9 PARS2 GBP4 EXTL2 SMC6 SMARCAD1 DHX57 HIPK1 FSTL1 STK36 ADORA1 RYK KIF15 MST1R RAD54L2 FASTK SYK WNK2 ATP7A TK2 HSP90B1 KIF7 PLK1 ACSF2 CDK12 MVD KIFC2 RAB26 ABCA3 TRANK1 PBK PPIP5K1 CSNK1G1 ARL13B CDK1 KIF5B MFN1 CLCN5 KSR2 PIK3CD SLFNL1 RAB33B QARS SUCLG2 SLFN11 DCAKD GRK2 GMPPB PC ADCY6 DDX23 ATR GK5 ARL10 MYO1D CHID1 DALRD3 MCMDC2 OPLAH FARSA CIITA BBS10 AATK ACBD4 ERCC6L2 UBA7 EPHA10 MX2 EP400 TBK1 CHEK2 SRPK3 UBE2G2 NRBP2 CDK10 RAD51D ROR1 POMK RTN4RL1 PIP5K1C KIF18B THBS2 KIF24 ERCC6L DYNC2H1 FANCM PALM3 RAB42 MAPK12 SBK1 ENTPD8 DYNC1H1 RAD54B SHPK RAB40C ATAD3A MYO1C SPG7 GRK6 RPS6KL1 UCKL1 PAPSS2 ABCA4 CDC42BPB MTOR MAP3K3 BMPR2 SKIV2L DHX16 DDX47 KIF4B PI4KA MARS2 PRKDC FPGT IKBKE STRADA AARSD1 ACACA PIP4K2B MYO19 PIGU PARP9 CXCL10 GPAA1 CTSK CTSS SEMA5A TMEM184A AGRN CHST12 HS3ST5 TPX2 LRP1 FGFBP1 PIGK |
| Down regulated | 8.25E-13 | 440 | Enzyme binding | ANKIB1 DBF4 ARHGAP44 RHOBTB2 ANAPC4 PKP2 UHRF1BP1 ARFGEF1 TBC1D25 TRAF5 CPNE3 RAB11FIP3 SH3BP1 PICK1 TAB1 PARD6A SH2D4A CAV1 RIC1 RGP1 CCNJ RAB11FIP2 RECQL5 CCND1 ARHGEF17 TCIRG1 ARHGDIB SASH1 CCNG1 RTKN STRN RIMS3 ARFGEF2 TRAP1 PAK4 YIPF2 RAB11FIP4 TBC1D5 LLGL1 TBC1D14 TBC1D8B CCNB1 CCNJL TBC1D4 PREB PLCE1 MAPK8IP3 FANCI NCOR1 SGSM2 EVI5L FANCD2 ADPRH TBCK CCNA2 USP6NL RNF144A UBE2L6 CCNB2 APPL1 LRRC14 DBF4B CCNF BICDL2 RHOBTB3 STXBP5 HPS6 LEO1 TBC1D2B TBC1D16 SPDYE5 QARS EPS8L2 SH2B1 AKAP5 MTA1 ARHGEF37 TBC1D9B SPDYE2 DENND6B DENND1C VPS52 EMP2 SPDYE3 MTCP1 RTEL1 IBTK MYCBP2 BRCA1 TPR WNK1 PKN2 RASGRP2 ELP1 HLTF PRKACA HACE1 AGO1 UNC13D LZTR1 HPS4 SBF1 PCIF1 CEP192 PARP4 TSC2 DMXL2 RAPGEF1 PPP3CB CDK5RAP3 CAMTA2 WFS1 PRKAR2A ECT2 XPO5 GGA3 ARHGEF16 APC RALGPS1 DAB2IP DENND4C SYTL2 ABI2 ITGAV RB1 ERBB2 SHPRH RAPGEF6 UVSSA ST5 HSP90B1 AKAP13 ADCY6 DENND4A DOK7 TMEM173 ANKFY1 HACD4 FLNA SFI1 DENND4B DZIP3 SMG5 CHML HACD2 UGT1A7 EID1 DVL2 CDKL5 PLEKHG6 PIAS1 CUL7 POLD1 MCM10 ROCK1 HYAL2 SREBF1 NSF NOTCH3 PPP1R12B DNAJC10 IPO11 AURKA TPX2 GCN1 KIF16B CBX5 TSPAN15 AP1B1 NIN RABGGTA E2F1 CSK HERC1 TNPO2 DNM1 ERLIN1 GBF1 RAPGEFL1 PPP1R9B RFC5 CUL9 DUSP22 EXOC2 HMGCR LMNB1 EHHADH CCDC88A STAT1 IL1R1 CDC20 CASP8AP2 AKAP7 PKD2 LRP1 FGD3 SDF2L1 XPO7 ATXN10 UBE4B ARHGEF9 FLOT2 ARHGEF11 SLF1 IPO8 LRP4 STX17 ARHGEF39 ARRB1 ADAM10 KSR1 TP53 AKT1 SYTL1 WDTC1 STRIP1 PARP1 IQSEC1 ERLIN2 PLA2R1 OBSCN MMS19 PPP1R9A SV2A RGL4 CALM3 PKN3 TGFBR2 SLC4A2 SYK ATP7A ARHGEF40 ANKRD2 SMAD3 CDK12 FADD STAT3 CDH2 KIF5B USP19 ANAPC2 BOK GLDC PTPN11 CDH5 NRIP1 GDPGP1 TBK1 IQGAP3 ARHGEF12 AP2A1 MAP3K5 CTNND1 IPO9 CDC42BPB MTOR INF2 PHACTR4 IPO7 UGT1A1 MICAL3 PRKDC IKBKE DET1 PJA2 CYFIP1 CFTR CASP10 ARHGAP33 ITGA3 NOX1 PKD1 SPDL1 YTHDC2 WWC3 TSPAN17 USP13 PMS1 ERBB3 NEDD4 PTPN3 SMG6 EXOC5 XRCC1 ADD2 KIFAP3 TOP2B UBE2T FAP TP73 WDR70 NCOA1 CAD MAST2 NOP14 MAVS ANAPC5 PUS7 WDR76 HDAC6 MSH2 MAP3K1 GGA1 TRIOBP TELO2 NFKBIA CDC25B KIF3B POLA1 TRADD MAPK3 LONP2 CEP152 IKBKB GYS1 HNRNPUL1 FAM83E DDX58 SUFU MTMR4 TRIM37 MAP2K6 SLC22A18 MLEC TNS2 FOXM1 GNB3 PTPN6 NEDD9 MAPK14 STK38 KIF20A DROSHA WWC1 ZNF346 SPTBN1 FN1 CHMP3 ATF2 DHCR24 ZNHIT6 GBP1 NEK2 KIF14 NEK9 SLF2 CAT OCRL MCM8 CEP250 PIN1 POT1 HERC2 TEP1 LATS1 KIF3A EXOC4 TOP2A PIAS3 TRIM22 DDC SYT11 AP3B1 SORT1 BORA CDK5RAP2 SORL1 RDX DBT KIF11 PARP9 PML PARN BCAS3 DYM PFKL ADAMTSL4 GOLGA4 DGKQ EPHA1 CDKN2B WWC2 UTRN BANK1 RRAGA PHF6 FBXW5 IFNAR2 HLCS MAML1 RACGAP1 RBBP4 PEX19 SMC6 FZD5 DTX3L MST1R FZD6 PLK1 STAT6 DIS3L STIM1 MAP4K2 SMAD1 LSM2 PARP14 CSPG4 CNTNAP2 ARHGAP1 TOM1L2 CALR FAM83H ERCC6L2 TUBB CHEK2 PKP3 AP3M1 BACE1 RXRA NOXA1 ARRDC1 HTT MAP1LC3C MYO1C PRC1 DCTN1 DIO2 FGFR1OP PVRIG PEX26 STRADA ERC1 CASP2 DUSP19 ACVR1B BARD1 UNC13B TBXA2R AKAP11 BLNK |
| Up regulated | 4.36E-14 | 452 | DNA-binding transcription factor activity | SOX8 TFAP2D SNAI2 RUNX3 TBPL1 ZIC2 FOXC1 PRDM1 PRDM6 GPBP1 KLF6 TCF7 TFAP2C LHX5 XBP1 SIX4 ELF4 KLF5 ZNF174 LHX2 LHX6 NKX3-2 ELK3 BACH2 PHF1 TBP CDX1 BCL6 ID2 DLX2 NFE2L2 TFAP2E PLAGL1 KLF9 BCL11A EGR1 EGR2 ESX1 SPDEF SOX4 RUNX2 SOX9 FOXA2 NR1D1 PRDM7 BCL11B KLF2 ATF4 SOX15 ZSCAN10 JUND GATA5 PRDM12 NPAS1 NFATC1 RARA PPARG ZMYM5 GRHL1 ETS1 EGR4 GATA4 KLF4 IRF4 ASCL1 IRF8 GATA6 LHX9 AFF3 DLX1 ZNF711 FOXO1 HHEX KLF10 ETS2 GRHL3 ISL2 ZNF222 IKZF3 GFI1 ZNF281 NKX6-1 KLF15 FOXQ1 SP8 SOX17 DRGX SOHLH1 E2F7 ZNF143 NKX3-1 SP2 ZNF646 ZNF597 IRF2BP2 GBX2 E2F6 ATF5 ZNF280A SIX2 FOXA3 ZNF296 SOX7 JUNB NHLH1 FOXB1 KLF11 ID4 ZNF131 OVOL1 NPAS4 ISX ETV4 FOXL1 FOXC2 JUN GATA2 ARID3B EGR3 PCGF5 MAFA NKX2-5 ZNF438 FOXL2 MAFF PURA IRF7 FOXD2 FOXD3 RFX8 MAFG MAFK MAFB SP5 ZNF155 FOXI3 SCRT2 SP9 ASCL5 ETV5 FOXD1 ZNF350 SCRT1 KLF14 SPIB LHX1 ZNF263 RORA TBX21 CREM HOXA1 GLI3 THAP1 ATF3 IRX2 DDIT3 ZBTB2 ZNF140 ARID5A MXD1 YBX1 MEF2A ENO1 TXK MECOM ESR1 HNF4A CRX HOXA5 AHR AEBP1 HAND1 HES1 TLX2 MEF2D NR4A3 TBX2 TWIST1 BATF3 TOX2 SNAI1 LRRFIP1 PAX8 GZF1 MEIS2 MYCN TBX3 LMO2 HEY2 SKIL MYC GCM1 EAF2 CREB5 NOTCH1 ZEB1 ARID5B CEBPG BACH1 IER2 PITX2 CREBRF HEY1 NFIL3 NR0B1 ZEB2 CNBP ZNF35 FOS HOXD8 HIC1 TGIF1 TAF7 PRKN NKRF ZKSCAN3 CEBPA GTF2IRD1 POU2F2 YBX3 TFE3 RUNX1T1 EBF4 PHF5A SMAD7 TSC22D1 MEIS3 PBX4 TLE4 PITX3 EBF3 GLI1 CUX2 NR2E1 SUB1 FOXP1 KCNIP3 KDM3A MYCL SATB2 HOXB8 SMAD9 NFYB TBX4 BHLHE41 NR4A1 FOSB FOXJ1 CBFA2T3 ARNTL TBR1 DMRT1 PGBD1 TAF4B DMRTB1 TBX19 MEIS1 CSRNP1 NR4A2 ZKSCAN2 TSC22D3 BTG2 PKNOX1 ZNF394 MSX1 EBF1 CITED2 PBX3 ZNF232 CTNNB1 BATF2 PCBP1 NPAS2 FOXN2 NEUROD2 CAMTA1 CEBPB ZNF483 FOSL1 FOXG1 BHLHA15 PCBP3 POU3F1 ZNF292 PAX5 TSC22D2 ZKSCAN5 ZSCAN5B ZNF165 DMBX1 ZBED9 SCX YBX2 ZNF200 SOX30 THAP3 ZNF275 HOXA9 ZNF416 TOX4 LRRFIP2 SALL4 ZNF14 ZNF574 DNAJC2 HBP1 IRX4 SFPQ RLF ZNF430 ZNF211 ZNF436 ZBTB1 HIVEP3 ZNF557 MNX1 ZNF426 CASZ1 NR0B2 EMX1 HLX DNAJC1 ZFP37 ZNF189 HMGA1 MBD1 DNMT3L ZNF787 PRDM16 ZNF697 OSR1 RBAK ZNF92 ZNF157 ZNF7 ZNF547 ZNF256 CXXC1 ZNF513 EN2 GFI1B PRRX2 ZNF610 ZNF528 ZNF701 ZNF83 INSM2 SHOX2 ZBTB43 ZNF311 BNC1 ZFPM2 ZNF778 ZNF212 ZNF16 HOXB9 ZNF160 ZNF415 ZNF581 ZNF562 HOPX ZIK1 ZNF540 ZNF57 ZNF556 ZNF554 ZNF596 ZBTB21 THAP2 ZNF266 ZNF654 ZNF408 ZNF77 ZNF114 ZNF543 ZNF552 HES7 ZNF771 ZNF816 ZFP3 ZNF707 ZNF329 ZNF101 TSHZ2 ZNF721 ZBTB7C ZNF829 ZNF267 ZNF555 ZNF286A ZNF383 ZNF669 ZNF567 ZNF600 ZNF250 ZNF836 ZNF565 ZNF34 ZNF799 ZNF846 ZNF431 ZFP28 ZNF100 ZNF398 ZNF441 ZNF695 ZNF665 ZNF841 ZNF433 ZNF460 ZNF461 ZNF44 ZNF790 ZNF823 ZNF121 ZNF560 ZNF334 ZNF442 ZNF583 ZNF511 ZNF627 ZNF277 TOX ZNF468 ZNF616 ZFP57 ZNF425 ZNF469 ZNF674 ZNF550 ZNF432 ZNF10 DUXA ZNF595 ZNF670 MAEL PKNOX2 ZFP36L1 BLZF1 CITED1 TCEAL1 ZNF622 TAF13 GAS7 SCML1 SP140 GABPB1 CIR1 LMO4 RCAN1 DRAP1 SUPT4H1 |
| Up regulated | 4.36E-14 | 85 | Structural constituent of ribosome | RPS20 RPS5 RPS19 RPS27A RPS3 FAU RPS29 RPL26L1 RPL31 RPL6 RPLP0 RPL3 RPS16 RPL19 MRPL27 RPS13 RPS12 RPL24 RPS15 RPL21 RPL23 RPL36 RPL27 DAP3 MRPL47 RPLP1 RSL24D1 RPL3L RPS11 RPL13A RPL11 RPS8 RPL10 RPL7 RPL30 RPL8 RPL29 MRPS18C RPL9 RPS14 RPL27A RPS9 RPS21 MRPL52 RPL38 RPL4 RPS27 RPL35A MRPL54 RPS27L RPS23 MRPL21 RPL12 RPS4X RPS28 MRPS6 RPLP2 RPL18 RPS18 RPL18A RPL28 RPL34 MRPL18 RPL22 MRPS15 RPL35 RPS6 RPS24 RPL32 RPS3A RPL37 RPL22L1 RPL39L RPL36AL RPL13 MRPL36 RPS7 RPS17 RPL14 RPL37A RPL10A RPL39 UBA52 MRPS24 MRPS36 |
| Up regulated | 4.55E-14 | 425 | DNA-binding transcription factor activity, RNA polymerase II-specific | SOX8 TFAP2D RUNX3 ZIC2 FOXC1 KLF6 TFAP2C LHX5 XBP1 SIX4 ELF4 KLF5 LHX2 LHX6 ELK3 BCL6 TFAP2E PLAGL1 KLF9 EGR1 EGR2 ESX1 SPDEF SOX4 RUNX2 SOX9 FOXA2 NR1D1 KLF2 ATF4 SOX15 JUND GATA5 NPAS1 RARA PPARG ZMYM5 ETS1 EGR4 GATA4 KLF4 IRF4 IRF8 GATA6 LHX9 FOXO1 HHEX KLF10 ETS2 ISL2 KLF15 FOXQ1 SP8 SOX17 DRGX SOHLH1 E2F7 SP2 IRF2BP2 GBX2 E2F6 ATF5 SIX2 FOXA3 SOX7 JUNB NHLH1 FOXB1 KLF11 ZNF131 OVOL1 NPAS4 ISX ETV4 FOXL1 FOXC2 JUN GATA2 ARID3B EGR3 FOXL2 PURA IRF7 FOXD2 FOXD3 SP5 FOXI3 SP9 ETV5 FOXD1 KLF14 SPIB LHX1 ZNF263 RORA TBX21 CREM ZNF174 HOXA1 GLI3 THAP1 ATF3 IRX2 ZBTB2 MAFF ZNF140 ARID5A MAFK SCRT1 SNAI2 TBPL1 PRDM1 MXD1 YBX1 MEF2A ENO1 TXK ESR1 CRX HOXA5 AHR AEBP1 HAND1 CDX1 TLX2 MEF2D NR4A3 BCL11A TBX2 TWIST1 BATF3 TOX2 SNAI1 LRRFIP1 GZF1 MEIS2 GRHL1 MYCN TBX3 LMO2 HEY2 SKIL MYC GCM1 ASCL1 EAF2 NOTCH1 ZEB1 ARID5B CEBPG BACH1 GRHL3 IER2 GFI1 ZNF281 PITX2 CREBRF HEY1 NFIL3 ZNF143 NKX3-1 NR0B1 ZEB2 CNBP DDIT3 HOXD8 HIC1 TGIF1 NKX2-5 PRKN NKRF ZKSCAN3 ZNF350 POU2F2 YBX3 TFE3 TCF7 EBF4 HNF4A MEIS3 TLE4 PITX3 EBF3 NKX3-2 GLI1 CUX2 BACH2 NR2E1 SUB1 HES1 KCNIP3 DLX2 NFE2L2 SATB2 BHLHE41 NR4A1 PAX8 FOSB BCL11B FOXJ1 ARNTL DMRT1 TBX19 MEIS1 CSRNP1 NR4A2 BTG2 PKNOX1 IKZF3 MSX1 NKX6-1 PBX3 PCBP1 FOS NEUROD2 CAMTA1 CEBPB FOSL1 BHLHA15 MAFA PCBP3 ZNF292 PAX5 MAFG DMBX1 MAFB CEBPA SCX YBX2 ZNF200 SOX30 THAP3 PRDM6 ZNF275 HOXA9 ZNF416 MECOM TOX4 LRRFIP2 SALL4 SMAD7 ZNF14 PBX4 ZNF574 DNAJC2 HBP1 TBP IRX4 FOXP1 SFPQ MYCL RLF ZNF430 HOXB8 SMAD9 NFYB TBX4 ZNF211 ZNF436 ZBTB1 HIVEP3 ZSCAN10 ZNF557 MNX1 ZNF426 CASZ1 NFATC1 NR0B2 EMX1 HLX DNAJC1 ZFP37 ZNF189 HMGA1 MBD1 DNMT3L ZNF787 PRDM16 DMRTB1 ZNF697 OSR1 DLX1 RBAK CREB5 ZNF92 ZNF157 ZNF711 ZNF7 ZNF547 ZNF256 CXXC1 ZKSCAN2 ZNF222 ZNF394 ZNF513 EBF1 EN2 GFI1B PRRX2 ZNF646 ZNF610 ZNF528 ZNF701 ZNF83 ZNF232 ZNF597 BATF2 INSM2 SHOX2 ZBTB43 ZNF311 ZNF280A BNC1 ZFPM2 ZNF35 ZNF778 ZNF212 NPAS2 ZNF16 ZNF296 HOXB9 FOXN2 ZNF160 ZNF415 ZNF581 ZNF562 HOPX ZIK1 ZNF540 ZNF57 ZNF556 ZNF554 ZNF596 ZNF483 ZBTB21 THAP2 ZNF266 ZNF654 ZNF408 ZNF77 FOXG1 ZNF114 ZNF543 ZNF552 HES7 ZNF771 ZNF816 ZFP3 ZNF707 ZNF329 ZNF101 TSHZ2 ZNF721 ZNF438 ZBTB7C POU3F1 ZNF829 ZNF267 ZNF555 ZNF286A ZNF383 ZNF669 ZNF567 ZNF600 ZNF250 ZNF836 ZNF565 ZNF34 RFX8 ZNF799 ZNF846 ZKSCAN5 ZNF431 ZFP28 ZNF100 ZNF398 ZNF441 ZSCAN5B ZNF165 ZNF695 ZNF665 ZNF841 ZNF433 ZNF460 ZNF461 ZNF44 ZNF790 ZNF823 ZNF121 ZNF560 ZNF334 ZNF442 ZNF583 ZNF511 ZNF627 ZNF277 TOX ZNF468 ZNF616 ZFP57 ZNF155 ZNF425 SCRT2 ZNF469 ASCL5 ZNF674 ZNF550 ZNF432 ZNF10 DUXA ZNF595 ZNF670 TSC22D1 ID2 PRDM12 TBR1 MAEL TSC22D3 PKNOX2 ID4 ZFP36L1 TSC22D2 GTF2IRD1 PRDM7 ZNF622 |
| Up regulated | 7.23E-14 | 529 | Transcription regulator activity | SOX8 TFAP2D SNAI2 RUNX3 TBPL1 ZIC2 FOXC1 PRDM1 PRDM6 GPBP1 KLF6 LMCD1 RUNX1T1 TCF7 RRN3 TFAP2C LHX5 XBP1 SIX4 TBL1X ELF4 TAF7L KLF5 ZNF174 MED26 AEBP1 LHX2 TLE4 LHX6 C1QBP NKX3-2 ELK3 BACH2 PHF1 TBP SUB1 CDX1 BCL6 HES1 ID2 DLX2 NFE2L2 TFAP2E PLAGL1 GTF3C3 KLF9 BCL11A ENY2 EGR1 EGR2 BHLHE41 ESX1 SPDEF SOX4 RUNX2 SOX9 FOXA2 CITED1 NR1D1 UXT PRDM7 BCL11B KLF2 ATF4 SOX15 CBFA2T3 ZSCAN10 JUND GATA5 PRDM12 NPAS1 MED18 NFATC1 RARA NR0B2 DNAJB1 PPARG WBP2 ZMYM5 MED10 GRHL1 ETS1 HEY2 EGR4 GATA4 KLF4 IRF4 ASCL1 TLE3 IRF8 TOB1 GATA6 BCL10 LHX9 AFF3 DLX1 ZNF711 FOXO1 HHEX KLF10 ETS2 GRHL3 MED8 ISL2 ZNF222 IKZF3 GFI1 ZNF281 NKX6-1 KLF15 FOXQ1 CITED2 SP8 HEY1 SOX17 DRGX SOHLH1 E2F7 COPS2 CDYL2 ZNF143 NKX3-1 SP2 ZNF646 ZNF597 CTNNB1 IRF2BP2 GBX2 E2F6 ATF5 NR0B1 ZNF280A SIX2 FOXA3 ZNF296 SOX7 JUNB NHLH1 FOXB1 KLF11 ID4 ZNF131 OVOL1 ZCCHC12 NPAS4 ISX DRAP1 ETV4 FOXL1 FOXC2 BASP1 JUN TAF7 HES7 GATA2 ARID3B EGR3 PCGF5 MAFA NKX2-5 ZNF438 FOXL2 TOB2 MAFF PURA IRF7 FOXD2 FOXD3 RFX8 TLE1 MAFG TAF13 MAFK MAFB SP5 ZNF155 FOXI3 SCRT2 SP9 ASCL5 TCP10L ETV5 FOXD1 ZNF350 SCRT1 KLF14 SPIB LHX1 ZNF263 RORA TBX21 CREM HOXA1 GLI3 THAP1 ATF3 IRX2 DDIT3 ZBTB2 ZNF140 ARID5A YAF2 WWTR1 MXD1 YBX1 MEF2A ENO1 TXK MECOM ESR1 HNF4A CRX HOXA5 AHR LPXN HAND1 TLX2 MEF2D NR4A3 TBX2 TWIST1 BATF3 TOX2 SNAI1 LRRFIP1 PAX8 GZF1 MED6 MEIS2 MYCN TBX3 LMO2 SKIL MYC GCM1 GTF2B CIR1 FGF2 TGFB1I1 EAF2 CREB5 NOTCH1 ZEB1 ANKRD1 ARID5B CEBPG BACH1 RBPMS NRG1 MED27 IER2 TFB2M PITX2 CREBRF NFIL3 TAF10 ZEB2 CNBP ZFPM2 ZNF35 FOS KAT5 HOXD8 HIC1 TGIF1 NPM1 BCOR PRKN UBE2L3 NKRF ZKSCAN3 HSPA1A NME2 CEBPA GTF2IRD1 POU2F2 YBX3 TFE3 SIRT6 ACTN2 EBF4 PHF5A TRIB3 SMAD7 TSC22D1 MEIS3 PBX4 PITX3 EBF3 AIP GLI1 CUX2 NR2E1 FOXP1 KCNIP3 KDM3A MYCL SATB2 HOXB8 SMAD9 NFYB TBX4 NR4A1 FOSB FOXJ1 ARNTL TBR1 DMRT1 PGBD1 TAF4B PRDM16 DMRTB1 TBX19 MEIS1 CSRNP1 NR4A2 ZKSCAN2 MED19 TSC22D3 BTG2 PKNOX1 ZNF394 MSX1 RYBP SFMBT1 EBF1 PCBD1 PBX3 ZNF232 BATF2 PCBP1 NPAS2 FOXN2 NEUROD2 CAMTA1 CEBPB ZNF483 FOSL1 FOXG1 BHLHA15 PCBP3 POU3F1 ZNF292 PAX5 TSC22D2 ZKSCAN5 ZSCAN5B C1D ZNF165 DMBX1 L3MBTL3 HSBP1L1 HSBP1 ZBED9 MCIDAS SCX JMJD6 CRYM EDF1 HMGA1 NUPR1 CCDC62 YBX2 ZNF200 SOX30 THAP3 ZNF275 HOXA9 ZNF416 TOX4 LRRFIP2 SALL4 ZNF14 ZNF574 DNAJC2 HBP1 IRX4 SFPQ RLF ZNF430 ZNF211 ZNF436 ZBTB1 HIVEP3 ZNF557 MNX1 ZNF426 CASZ1 EMX1 HLX DNAJC1 ZFP37 ZNF189 MBD1 DNMT3L ZNF787 ZNF697 OSR1 RBAK ZNF92 ZNF157 ZNF7 ZNF547 ZNF256 CXXC1 ZNF513 EN2 GFI1B PRRX2 ZNF610 ZNF528 ZNF701 ZNF83 INSM2 SHOX2 ZBTB43 ZNF311 BNC1 ZNF778 ZNF212 ZNF16 HOXB9 ZNF160 ZNF415 ZNF581 ZNF562 HOPX ZIK1 ZNF540 ZNF57 ZNF556 ZNF554 ZNF596 ZBTB21 THAP2 ZNF266 ZNF654 ZNF408 ZNF77 ZNF114 ZNF543 ZNF552 ZNF771 ZNF816 ZFP3 ZNF707 ZNF329 ZNF101 TSHZ2 ZNF721 ZBTB7C ZNF829 ZNF267 ZNF555 ZNF286A ZNF383 ZNF669 ZNF567 ZNF600 ZNF250 ZNF836 ZNF565 ZNF34 ZNF799 ZNF846 ZNF431 ZFP28 ZNF100 ZNF398 ZNF441 ZNF695 ZNF665 ZNF841 ZNF433 ZNF460 ZNF461 ZNF44 ZNF790 ZNF823 ZNF121 ZNF560 ZNF334 ZNF442 ZNF583 ZNF511 ZNF627 ZNF277 TOX ZNF468 ZNF616 ZFP57 ZNF425 ZNF469 ZNF674 ZNF550 ZNF432 ZNF10 DUXA ZNF595 ZNF670 MAEL PKNOX2 ZFP36L1 HDAC9 CCNE1 FHL2 BLZF1 BTG1 CDK7 GRIP1 IL31RA TCEAL1 ZNF622 NACA GAS7 SCML1 SP140 PQBP1 GABPB1 NFKBIB HTATIP2 SSX1 CBX4 LMO4 SAP18 RCAN1 PAWR SIAH2 SUPT4H1 |
| Up regulated | 1.36E-12 | 484 | Molecular function regulator | NGEF VAV3 VAV1 CX3CL1 CCL26 SEMA3B GCLM PRSS8 GAL HSP90AA1 GCKR PPP1R15A PTHLH ARHGAP28 FXYD5 SEMA6A ADA2 IL11 OSM TIMP3 NCF4 BMP7 RENBP TIMP1 RGCC CCL22 LHB OAZ1 CCNE1 CNTD2 CLEC11A TFPI2 CCL24 SERPINE1 SEC61B AMBP CUBN DKK1 CSF3 PPY AREG GRPEL1 MDK TBC1D30 COX6A1 CCNC VEGFA HBEGF IL12B HRG CISH CCL20 AUP1 SGK1 CCND2 PGF SOCS2 NPPB CSTA TBC1D15 INHBA SERPINA7 CKS2 PREX1 CXCL6 BMP4 IL1B IL37 BMP2 WNK4 LIF PRKRIP1 ATP1B2 APOE GDF15 ADRM1 EPS8L1 PYY CCNA1 NGF CCNH ANXA1 PKIB KCNMB4 SERPINE2 DNAJB2 IL36G IL36A RPLP1 MSTN CCNG2 ANXA3 INHBE REM2 OSGIN1 PIK3R5 BCL10 AZIN2 EIF2B5 TSLP VIP GNRH1 SERPINH1 FXYD4 VEGFC KCNE4 BMP6 AZIN1 NODAL NRG1 NCF1 RCAN1 CSTB IL6R INHBB SERPINI1 CCNL1 CXCL3 UCN AIMP1 GRM2 CSF2 GDF9 TAGAP GEM VEGFD SCN3B SERPINB8 RRAD CACNG2 GPRC5B HEXIM2 CXCL8 SLC30A1 GPRC5C SERPINB9 CKS1B SH3BP5L INHBC TBC1D10C CLCF1 KCNE5 ERFE ANXA2 RGPD6 CSF1 GAST SOCS3 SOCS1 HEXIM1 SEMA4D QRFP CGB5 CGB7 SERPINA1 GUCA2A SERPINB2 PPP1R14C CSNK2B IL31 SERPINB5 CGB8 LEFTY1 SERPINE3 MTRNR2L3 INAFM1 TBC1D3G TBC1D3D CCL3L3 WNT3 WNT1 LGALS3 SPRY2 CRLF1 APBA3 LTF NEDD4L DNAJA2 CST7 DNAJA1 MT3 IGBP1 APOH CYTH4 AHSA1 GSKIP ANGPT4 WFDC2 WNT2 DNAJB6 STX1A PCOLCE DOCK8 BAG1 VSIR PFN1 RANGRF LAMTOR3 BAG2 FGF1 SUMO1 ARHGEF2 ITGB1BP1 HSPH1 CCZ1 ARHGAP9 SLPI KCNS1 CDKN1A RPL23 TNFSF14 EDN2 DOCK4 SEC14L1 CACNG6 DNAJB1 CGA CAB39 IL6 RASGEF1B FGF2 WARS DNMT3L APP RGS16 CALM2 CAMK2D BOD1 IGFBP3 DENND2A C9orf72 NOTCH1 RGS10 IL18 ITPR1 BAG3 SPOCK1 RASGRP3 RABGEF1 GPSM1 DNAJB4 PCOLCE2 SPDYA PRKCD DLC1 VCP A2ML1 NKX3-1 IGF2 DUS2 NLRP7 RAB3IL1 RHOH IL12A MAP2K1 RGS14 ADRB2 RASGRP4 BCL2 RPS7 IL17D CHRNA7 DENND2C CDK5R1 SMCR8 JUN NPM1 RGS6 RGS7 SPATA13 RPS27L LYPD6 PDGFA RASGEF1A TNF RACK1 DDX3X FNIP1 MIF PINX1 IL32 TG KITLG MCF2L2 RASGRF1 SCT ACAP1 PPP2R2C ARHGEF1 PHACTR3 DOCK3 ARHGAP4 THPO HSP90AB1 MLN HSCB TRIB3 WFDC1 AMH CDC37 CYTH2 FGF21 RAB3A HAMP NAMPT HSPB1 TNFSF8 PSMD3 CALCA IL23A PHACTR1 THBS4 STC2 FGF12 IL1A KCNIP3 RAP1A NCF2 RGS2 HPCAL4 SIPA1L2 RGS4 ARTN TNFSF4 SPP1 PPP1R3C SPINK4 WFDC3 IL17C TNFSF9 MCF2L ADM2 RAC2 VGF ARHGAP22 ARHGEF6 CDKN1C STARD8 GMFG SESN2 RAP1GAP2 ALOX5AP STARD13 DOCK10 HTR2B KLF4 BCAR3 CMTM3 RGL1 ARF1 UCN2 PSD2 RASGEF1C DOCK11 ITPRIP ADM CDC42EP2 LYPD1 PPP1R1C CMTM7 FLCN FGF18 PPP2R2B DGKI NBL1 STC1 GIP FNDC5 TFF1 TNFSF13 FGF19 BSND CAMK2N1 IL20 IL24 ARHGAP25 NPPC COL6A3 PSMD6 CAMK2N2 SPINK1 CDC20B CASP3 PPP1R36 SPRED1 SCG5 ANGPTL4 FNTA GDNF CMTM8 NRTN RLN3 APLN CDK5R2 IL16 PPP1R3B TRIB1 LTA RIN1 PSMD2 CST6 CABP4 NUPR1 METRNL UMODL1 MYOZ1 CEP295NL GREM2 TNFSF15 PPP1R27 DAZAP2 FLRT2 CDNF RASA3 RINL C17orf99 VSTM1 SRGAP1 SPRED2 PPP1R10 GPSM3 COL28A1 FAM19A5 CERS1 ELFN1 UBE2L3 WNT11 NRP1 STX4 MAPK8 PSMD14 NCKAP1L PRDX5 SH3BP4 FST TANK RPL11 FOXL2 IRGM ABCC9 GNAS CACNG1 NRXN2 CPEB2 ADGRV1 ZP3 SIRPA ATP6V1H DRD4 NLRP1 ESR1 JAG1 ENDOU NLRP12 PPP4C SHH ZEB2 YWHAG MAL SNCB CHGB SPOCK2 RUNDC3A ERRFI1 SET MAPK8IP1 MMP24 SAG RCBTB2 DNAJC1 BNIP2 RGS5 IQGAP2 RGS20 MMP16 ANXA5 ARF4 KCNS3 ALK SFN ANKLE2 NMB MMP17 GCGR |
| Up regulated | 2.83E-12 | 973 | Nucleic acid binding | FKBP4 RPS20 AKAP8L UTP18 NOP16 CELF2 MRTO4 NOP58 USP36 ZC3H11A YBX3 MRPS24 YBX1 DNTTIP2 CFAP20 RPL31 BUD23 ENO1 FSCN1 RBM7 ARHGEF1 HSP90AA1 BZW1 RPS5 YTHDC1 TRIP6 RPL6 RPLP0 RPS18 HSP90AB1 LGALS1 SNU13 RPL3 EIF3D PHF5A EIF5 PSMC1 TPD52L2 NOP56 MAPRE1 ALG13 PIN4 RBM3 UBE2I EIF3E TRMT1 NOVA2 FAM32A RPS16 CCDC9 RPS19 NOP53 RPL18A DNAJC2 SSBP1 HSPB1 PDAP1 SEC61B EDF1 CPEB3 GTPBP4 GLRX3 RPL28 RPL19 PFN1 DDX5 MRPL27 LRRC59 FRG1 HSPA8 RPS13 RSRC2 KRT18 MAGOHB RPS12 HSPA9 SUB1 CPEB4 RPL24 FXR1 EIF1B SF3B6 RPS15 RTN4 CCT4 HSPE1 SRSF7 SUMO1 RPL22 KIAA1324 SFPQ MRPS15 RPF1 EBNA1BP2 PRDX1 SYF2 RPS25 SLIRP SMNDC1 TCP1 ETF1 ZCCHC17 RPL21 DDX39A RPL23 EIF2S2 SBDS HNRNPH2 NGDN RPL36 EIF3G SYNE1 RBM39 RPL27 KHDRBS3 LGALS3 RAN UTP3 DAP3 RBM38 DMGDH TPT1 CCDC59 PRPF38B YARS PRPF38A DHX34 MSI1 TES MRPL44 TRA2B TXN RPL35 RPS6 CPEB2 RTCA RPS24 SRP14 EIF4A3 DUS3L RPS11 RPL13A NOSIP RPL11 RPS8 SF3B4 ARF1 RPS27A HSPD1 DYNC1LI1 RPL32 RPS3A BTF3 CCT6A RPL10 RPL7 EIF3H SERPINH1 RPS3 FAU ALDOA SAP18 UBC NIFK ADK SCAF4 RPL30 SUPV3L1 EIF4A2 RBPMS HIST1H4H CSRP1 ZYX CSTB CHTOP RPL8 EIF4A1 RPL29 MAGOH C1orf52 ZNF326 RBM15 TFB2M CCT3 SNIP1 SLBP NSA2 RPS14 RP9 VCP ZCCHC24 RPL27A ACAA2 RPL13 SRP68 COA6 HIST1H1E XIRP1 STIP1 PCBP1 CNBP YWHAG LSM3 RPS9 TBCA RPS21 MRM3 RPS7 ZNF622 EIF1 RBM4B RPL4 LSM1 MEX3C JUN HNRNPA0 RPS27 RPP25 CLK3 CDC42EP4 APOBEC3B NQO1 NPM1 CFAP65 MEX3D ARL6IP4 ANXA2 NGRN RPS17 RPL35A MEX3B UTP11 PCBP3 MRPL54 HIST1H1B RPS27L PURA CCDC137 ZFP36L1 UBE2L3 NKRF RPS23 HIST1H1C S100A16 RPL14 SPATS2L PPIA MRPL21 RPF2 RPL37A RPL12 AKAP17A RPS4X YTHDF2 RPL10A HSPA1B HSPA1A PPP1R10 RACK1 CSNK1E FASTKD5 DDX3X EXOSC6 NOL7 RPS28 PEG10 ZCCHC3 HIST1H4K DUSP14 HIST1H4I HIST1H4E MRPL45 RECQL TFAP2D ERCC1 SNAI2 SNAPC1 TBPL1 RPL26L1 ZIC2 PRDM1 PRDM6 KLF6 MEF2A MOV10L1 TCF7 TFAP2C LHX5 ORC6 DAZL CDC6 XBP1 SIX4 RAE1 EEF1A2 DNTTIP1 TAF7L ZNF174 GABPB1 BRF2 TFPT ZNF574 HOXA5 CHCHD2 LHX2 LHX6 SETX PITX3 C1QBP NKX3-2 CUX2 MRPL18 BACH2 RBM24 PHF1 TBP CDX1 BCL6 HES1 KDM3A DLX2 NFE2L2 MEF2D TFAP2E PLAGL1 DCLRE1B SATB2 NR4A3 BCL11A MTRF1 EXOSC8 NFYB BHLHE41 NR4A1 ESX1 BATF3 ZBP1 HIST1H2BJ FOSB SNRPB2 NR1D1 PRDM7 BCL11B ATF4 APOBEC3A PRKRIP1 JUND GATA5 PRDM12 NFATC1 RARA PPARG WBP2 H3F3B KDM6B TOE1 GRHL1 ANXA1 HEY2 EGR4 GATA4 APTX RSL24D1 GTF2B ASCL1 SRRM4 TAF4B GATA6 MBD1 ZNF473 CTU1 MAEL LHX9 OSR1 DQX1 AFF3 DLX1 EIF2B5 KDM6A ZNF711 NXF3 ARID5B FOXO1 HHEX NR4A2 TDRD9 GRHL3 HIST1H2BD CIART MED8 DEDD2 ZNF394 CELF5 U2AF1L4 IKZF3 MEIOB GFI1 ZNF281 ATF3 H3F3A MSX1 MRPS18C EIF4E3 CWC22 RPL22L1 NKX6-1 ZNF513 SLC4A1AP AIMP1 H2AFZ PITX2 HEY1 EN2 DRGX C12orf50 E2F7 ZNF143 RBPMS2 NKX3-1 RBFOX3 ZNF646 ZNF597 BATF2 IRF2BP2 GBX2 HEXIM2 E2F6 ATF5 NR0B1 ZNF280A FOS SIX2 ZNF296 JUNB CAMTA1 NHLH1 JMJD1C ZNF131 OVOL1 ENDOV DDIT3 ZNF408 ISX FOSL1 BASP1 MLF1 TAF7 EIF3K HES7 GATA2 ARID3B EGR3 HIST1H2AC HIST1H2BC PCGF5 HIST3H2A DDN NOP10 MAFA NKX2-5 HIST2H3D ZNF438 XPOT HIST2H2BE MAFF RBM11 HEXIM1 H1FNT H2AFX FBLL1 ZNF836 LONP1 YRDC RFX8 ARID5A HIST3H2BB MAFG HIST1H3J HIST1H3D ZNF841 TAF13 HIST1H2BK MAFK HIST2H2AA3 HIST2H2BF MAFB SUPT4H1 CPEB1 SCRT2 ASCL5 HIST1H2BN APOBEC3G MRPS6 ZNF350 SCRT1 HIST2H2AA4 LHX1 HIST1H2BG HIST1H3G HIST1H2BE HIST1H2BO HIST1H3E HIST1H3I HIST1H3A U2AF1L5 HIST1H2AL HIST1H2AE HIST1H3C HIST1H3H EIF3I EIF3J GTF3C3 EIF3M ZNF263 CRY1 VIM POU2F2 SARS SOX30 FOXC1 MXD1 RORA JMJD6 TBX21 TXK RRN3 WNT11 ESR1 CREM HNF4A SMAD7 TBL1X ELF4 PQBP1 KLF5 CRX MEIS3 HOXA1 AHR GLI3 AEBP1 HDAC5 GLI1 ELK3 ENDOU HAND1 TLX2 CR2 COLEC11 EGR1 TBX2 TWIST1 EGR2 SLPI TOX2 SNAI1 SPDEF SOX4 RPP40 LRRFIP1 WNT1 SOX9 PAX8 FOXA2 GZF1 CITED1 PRDX5 FOXJ1 RFTN1 THAP1 IGHMBP2 ARNTL MEIS2 MYCN PIWIL4 ETS1 TBX3 LMO2 SKIL HLX KLF4 MYC IRF4 GCM1 HMGA1 IRF8 PRDM16 SDE2 MEIS1 EAF2 NOTCH1 ANKRD1 TUT1 CCT5 CEBPG CXXC1 KLF10 ETS2 IER2 TIPARP KLF15 CREBRF SLU7 NFIL3 LARP6 TERF2IP SP2 DUS2 MRPL58 ING2 ZEB2 ZNF35 NPAS2 IRX2 BCL2 KLF11 ISG20 CEBPB ZBTB21 NPAS4 ETV4 HOXD8 NUPR1 FOXL1 FOXC2 HIC1 TGIF1 BEND3 EXOSC4 ZBTB2 FOXL2 PRKN IRF7 POU3F1 FOXD2 FOXD3 ZKSCAN3 ELAVL3 ZNF140 PIWIL2 C1D TNF CRIP1 RPP21 NME2 ETV5 CEBPA FOXD1 PINX1 SCX KLF14 SOX8 YBX2 GTF2IRD1 ZNF200 LTF RUNX3 THAP3 GPBP1 ZNF275 ZNRD1 TFE3 POLB REXO2 HOXA9 RUNX1T1 SP140 DDX43 DUSP12 ZNF416 MECOM ZFAND6 EBF4 BRAP TOX4 TFIP11 SALL4 RNMT POLR2C UBXN8 ZNF14 PBX4 HBP1 DNAJB6 ZFAND5 EBF3 NR2E1 TARS IRX4 FOXP1 EEF1B2 KCNIP3 POLE4 CHD5 GADD45A MYCL RLF ZNF430 KLF9 SET HOXB8 SMAD9 PAIP2 TBX4 ZNF211 EEPD1 RUNX2 ZNF436 SSX1 ZBTB1 HIVEP3 KLF2 RBM48 ELL3 SOX15 ZSCAN10 ZNF557 MNX1 NPAS1 ZNF426 CASZ1 HABP4 NR0B2 MED6 EMX1 TBR1 DNAJC1 ZFP37 ZNF189 ENPP2 DMRT1 THUMPD2 ASCC1 ADAMTS17 CBX4 GRB7 APP ZNF787 DMRTB1 LMO4 ZNF697 TBX19 TAF1A ZNF385B CSRNP1 RPL37 ZMAT2 PNLDC1 RBAK CREB5 ZNF92 ZNF157 ZNF7 ZEB1 EEF1AKMT1 PDCD4 NOCT XRCC4 ZNF547 ZNF256 SREK1IP1 ELOC RABGEF1 ZKSCAN2 BACH1 ZFAND3 BRPF1 RCAN1 ISL2 ZNF222 PKNOX1 ZNF761 RNASEK-C17orf49 NLRP3 TDRD10 HELQ EIF5A2 RYBP RPL9 TIGD6 EBF1 FOXQ1 SP8 SOX17 PKNOX2 SOHLH1 ZNF503 GFI1B RNASE7 TAF1D TAF10 ZMAT1 MARS PBX3 PRRX2 ZNF610 ZNF528 ZNF701 ZNF83 ZNF232 MAJIN INSM2 CDKN2AIP SHOX2 ZBTB43 ZNF311 BNC1 ZFPM2 SF3B5 ZNF778 ZNF212 FOXA3 ZNF16 HOXB9 FOXN2 ZNF160 ZNF415 SOX7 ZNF581 ZNF562 HOPX NEUROD2 ZIK1 ZNF540 FOXB1 ZNF57 ZNF556 ZNF554 SMN1 BANP ZNF596 ZNF483 THAP2 NABP1 TIGD3 GTF2IRD2B ZCCHC12 ZNF266 ZNF654 BANF1 EIF1AD DRAP1 ZNF77 FOXG1 KCMF1 ZNF843 CHD9 RBM44 ZNF114 ZNF543 ZNF552 EXD1 ZNF771 PSTK ZNF816 BHLHA15 YOD1 ZFP3 ZNF707 ZNF329 ZNF101 SNRPE TSHZ2 ZNF721 BCOR ALYREF ZNF703 ZBTB7C SMYD3 ZNF829 ZNF267 POLR1D ZNF555 NANOS3 ZNF286A EXD3 SAMD11 C11orf95 ZNF383 ZNF669 GTF2F2 NANOS2 ZNF292 ZNF567 ZNF600 SYCP2 PAX5 ZNF250 GTF2IRD2 ZNF565 ZNF34 ZNF799 NACA ZNF846 ZKSCAN5 ZNF431 ZFP28 ZNF100 ZNF398 ZNF441 ZSCAN5B ZNF165 ZNF695 ZNF665 DMBX1 ZNF433 ZNF460 ZNF461 ZNF44 ZNF790 ZNF823 ZNF121 ZNF560 ZNF334 ZNF442 ZNF583 ZNF511 ZNF627 ARC ZNF830 ZNF277 TOX TSEN15 RBM20 OOEP SP5 GPANK1 ZNF468 ZNF616 ZFP57 ZNF155 ZNF425 ZBTB10 SMN2 DPF3 RPS29 ZNF888 FOXI3 SP9 ZNF630 ZNF469 KLLN ZBED9 EIF6 ZNF674 ZNF550 TRNP1 ZNF432 ZNF10 ZNF486 C17orf49 BCL2L2-PABPN1 DUXA SPIB ZNF595 ZNF670 SULT2B1 CARS MMP12 TEX19 ACR BLZF1 TRIM26 TSPYL2 SCML1 RPL18 RPL34 RNF11 PSMA1 RPL3L LENEP RPL38 RPL39 |
| Up regulated | 2.83E-12 | 362 | Protein dimerization activity | SLC11A1 ASNS HSP90AA1 NLRC4 TYRP1 CUBN FXR1 CDHR3 CDH15 ANXA1 DMRT1 BCL2L10 BCL2A1 CDH13 DMRTB1 MCL1 TENM4 BCL2 NECTIN3 GGN PDE2A BCL2L2-PABPN1 CRLF1 FLT4 BUD23 STK10 ST6GAL1 ENO1 TPD52 ACTN2 CST7 CEACAM6 TFAP2C HSP90AB1 XBP1 HMOX1 HNF4A DNTTIP1 GLA CDADC1 PSMD7 CRYM GABPB1 RIPK2 BNIP3L RPS19 RASIP1 GARS HSPB1 AHR VWF TPI1 VEGFA STC2 ACVR1 GADD45A CR2 DCLRE1B VPS4B GLIPR2 LRRFIP1 BMP2 CITED1 PRDX5 ZBTB1 APOE GDF15 PDLIM4 RARA THAP1 PPARG GSTM3 SDS TUBB2B TLR2 AOX1 WARS ATP6V1C2 NOTCH1 HSPB8 BMP6 CEBPG CDA SCUBE1 ABCG1 IKZF3 ATF3 S100A11 S100P AIMP1 ACSL6 GDNF ADRB2 ADRA1B FOS IL17D AGXT TRMT112 NPAS4 DDIT3 CHRNA7 CLCF1 BNIP3 JUN ERN1 PLD6 NPM1 NOG ADRA2C CSF1 NSMCE3 RBM11 KRT10 ADRB3 MME PDGFA S100A10 S100A6 FICD RACK1 HLA-G PDCD6 SCX IKBKG HSPB6 SOX8 ITGAL NOS2 MGST1 BID WWTR1 GCLM MXD1 PRDM6 DGAT2 MEF2A TFE3 JMJD6 PTGS2 EBF4 SLC8B1 WHRN LGALS1 PVALB ST13 NTSR1 MYOM1 RENBP POLR2C HOMER2 KCNN1 MAG NAMPT STX1A EBF3 ENO3 SLC6A4 GRPEL1 CHKA HAND1 IL12B HES1 POLE4 ID2 ODC1 MEF2D TFAP2E MYCL NR4A3 PGF NFYB INHBA TWIST1 BHLHE41 NR4A1 HIST1H2BJ SOX4 SOX9 SOX15 SYT5 KIF1A PLVAP NPAS1 ANO1 H3F3B ALOX5AP ARNTL SYT6 MYCN HEY2 MYC SDCBP RBP4 MSTN ASCL1 TAF4B ASGR1 MYOM3 MEIS1 STOM LCN2 PTS BTBD11 QDPR ANO4 BCL2L11 NR4A2 CABYR HIST1H2BD HIST1H4H PKNOX1 ITGB2 CHRNB2 SQSTM1 H3F3A CRYBA2 H2AFZ EBF1 HEY1 SOX17 VEGFD SOHLH1 ABTB2 RBPMS2 TPM4 CTNNB1 E2F6 NPAS2 APLN NEUROD2 NHLH1 ID4 CEBPB ADCY5 SLC22A1 BANF1 DRAP1 CIDEA GJC3 RBM44 ERFE HES7 BHLHA15 HIST1H2AC HIST1H2BC GREM2 HIST3H2A USH1G IZUMO1 NKX2-5 HIST2H3D HIST2H2BE ADSSL1 POLR1D H2AFX S100A16 SYCP2 CTSE HIST3H2BB MAFG HIST1H3J HIST1H3D DMBX1 TAF13 HIST1H2BK TPM2 HIST2H2AA3 HIST2H2BF MAFB ASCL5 HIST1H2BN CRYBB2 CEBPA HIST2H2AA4 HIST1H4K HIST1H2BG HIST1H3G HIST1H2BE HIST1H2BO HIST1H3E HIST1H3I HIST1H3A HIST1H4I HIST1H2AL HIST1H4E HIST1H2AE HIST1H3C HIST1H3H RRAGD RRAGB OLFM4 CARS RRAGC PRDX6 ARF1 MITD1 NBL1 TMCC1 CD247 SARS KCNH2 ACHE IRAK3 ADA2 ACOT7 CHMP4B VAPA ZNF174 AMBP CRYAB PPP2CA CACYBP SFPQ MASP1 TPST2 SNX9 AOC3 NR0B2 GCH1 RAN MTUS2 IRAK2 GRHL1 STK26 GABBR2 CAMK2D PSPH RPL7 HHEX SUPV3L1 RBPMS XDH IL6R INHBB RCHY1 CHMP4C CRYL1 HPRT1 IL12A NR0B1 TPST1 TAF7 FZD9 S100A13 PTPRT ATP2A1 ASMT SUPT4H1 SYNE1 KCNB1 ACOX2 EXD1 BHLHB9 P2RX7 TARS ATF4 SOD1 MAFA APOBEC3G NCF4 NOTCH4 ACTN3 |
| Up regulated | 5.99E-11 | 226 | RNA polymerase II regulatory region sequence-specific DNA binding | TFAP2D TBPL1 ZIC2 PRDM1 PRDM6 MEF2A TCF7 TFAP2C LHX5 XBP1 HOXA5 LHX2 LHX6 PITX3 NKX3-2 CUX2 BACH2 TBP CDX1 BCL6 HES1 DLX2 MEF2D TFAP2E PLAGL1 NR4A3 NFYB BHLHE41 NR4A1 ESX1 BATF3 FOSB NR1D1 PRDM7 ATF4 JUND GATA5 PRDM12 NFATC1 RARA PPARG KDM6B HEY2 EGR4 GATA4 GTF2B ASCL1 GATA6 LHX9 OSR1 DLX1 KDM6A ZNF711 HHEX NR4A2 CIART MED8 ATF3 MSX1 NKX6-1 ZNF513 PITX2 HEY1 DRGX NKX3-1 BATF2 IRF2BP2 GBX2 ATF5 FOS JUNB NHLH1 ZNF131 DDIT3 ISX FOSL1 JUN HES7 GATA2 ARID3B EGR3 PCGF5 DDN MAFA NKX2-5 MAFF PURA NKRF RFX8 MAFG MAFK MAFB ASCL5 LHX1 SNAI2 SARS FOXC1 MXD1 YBX1 KLF6 RORA ENO1 TXK ESR1 CREM ELF4 KLF5 CRX GLI3 AEBP1 HDAC5 GLI1 ELK3 HAND1 BCL11A EGR1 TBX2 TWIST1 EGR2 TOX2 SNAI1 SOX4 LRRFIP1 SOX9 PAX8 FOXA2 GZF1 THAP1 H3F3B ARNTL MEIS2 GRHL1 MYCN ETS1 TBX3 LMO2 SKIL MYC IRF4 GCM1 IRF8 EAF2 NOTCH1 ARID5B KLF10 ETS2 IER2 ZNF281 H3F3A KLF15 H2AFZ CREBRF NFIL3 ZNF143 SP2 E2F6 CNBP CEBPB NPAS4 ETV4 HOXD8 FOXC2 TGIF1 ZBTB2 IRF7 FOXD3 ZKSCAN3 ZNF350 SCX SCRT1 SOX8 CRY1 POU2F2 YBX3 TFE3 HOXA9 EBF4 SIX4 HNF4A EBF3 KCNIP3 KDM3A NFE2L2 SFPQ GADD45A SATB2 RUNX2 BCL11B FOXJ1 TBR1 KLF4 DMRT1 TBX19 MEIS1 ZEB1 CEBPG BACH1 IKZF3 FOXQ1 PKNOX2 E2F7 PBX3 SIX2 HOXB9 NEUROD2 OVOL1 BHLHA15 BCOR FOXL2 SMYD3 PAX5 DMBX1 ZNF277 SP5 CEBPA FOXD1 WBP2 RPS3 ETV5 SPIB RUNX3 AHR NPAS2 ZNF431 TNF SCRT2 |
| Up regulated | 3.55E-10 | 303 | Sequence-specific DNA binding | TFAP2D SNAPC1 TBPL1 ZIC2 PRDM1 PRDM6 MEF2A TCF7 TFAP2C LHX5 ORC6 CDC6 XBP1 SIX4 ZNF174 BRF2 HOXA5 CHCHD2 LHX2 LHX6 SETX PITX3 NKX3-2 CUX2 BACH2 PHF1 TBP CDX1 BCL6 HES1 KDM3A DLX2 NFE2L2 SFPQ MEF2D TFAP2E PLAGL1 SATB2 NR4A3 BCL11A NFYB BHLHE41 NR4A1 ESX1 BATF3 FOSB NR1D1 PRDM7 BCL11B ATF4 JUND GATA5 PRDM12 NFATC1 RARA PPARG KDM6B GRHL1 HEY2 EGR4 GATA4 GTF2B ASCL1 GATA6 MBD1 MAEL LHX9 OSR1 DLX1 KDM6A ZNF711 FOXO1 HHEX NR4A2 GRHL3 CIART MED8 ATF3 MSX1 NKX6-1 ZNF513 PITX2 HEY1 EN2 DRGX E2F7 NKX3-1 ZNF597 BATF2 IRF2BP2 GBX2 E2F6 ATF5 NR0B1 FOS SIX2 ZNF296 JUNB CAMTA1 NHLH1 JMJD1C ZNF131 OVOL1 DDIT3 ISX FOSL1 JUN HES7 GATA2 ARID3B EGR3 PCGF5 DDN MAFA NKX2-5 ZNF438 MAFF PURA NKRF RFX8 MAFG MAFK MAFB ASCL5 LHX1 ZNF263 SNAI2 POU2F2 SARS SOX30 FOXC1 MXD1 YBX1 KLF6 RORA TBX21 ENO1 TXK RRN3 ESR1 CREM ELF4 KLF5 CRX MEIS3 HOXA1 AHR GLI3 AEBP1 HDAC5 GLI1 ELK3 HAND1 TLX2 EGR1 TBX2 TWIST1 EGR2 TOX2 SNAI1 SPDEF SOX4 LRRFIP1 SOX9 PAX8 FOXA2 GZF1 FOXJ1 THAP1 H3F3B ARNTL MEIS2 MYCN ETS1 TBX3 LMO2 SKIL HLX KLF4 MYC IRF4 GCM1 HMGA1 IRF8 PRDM16 EAF2 NOTCH1 ARID5B CEBPG CXXC1 KLF10 ETS2 CHTOP IER2 ZNF281 H3F3A KLF15 H2AFZ CREBRF NFIL3 ZNF143 TERF2IP SP2 ZEB2 CNBP ZNF35 IRX2 BCL2 CEBPB ZBTB21 NPAS4 ETV4 HOXD8 FOXL1 FOXC2 HIC1 TGIF1 BEND3 ZBTB2 PRKN IRF7 POU3F1 FOXD2 FOXD3 ZKSCAN3 LONP1 ZNF140 ARID5A CRIP1 FOXD1 ZNF350 SCX SCRT1 KLF14 SOX8 CRY1 YBX3 TFE3 HOXA9 EBF4 HNF4A PBX4 EDF1 EBF3 NR2E1 SUB1 IRX4 FOXP1 KCNIP3 GADD45A HOXB8 RUNX2 ZSCAN10 MNX1 EMX1 TBR1 DMRT1 DMRTB1 LMO4 TBX19 MEIS1 CSRNP1 ZEB1 BACH1 ISL2 PKNOX1 IKZF3 NLRP3 FOXQ1 SOX17 PKNOX2 PBX3 PRRX2 SHOX2 PCBP1 FOXA3 HOXB9 FOXN2 NEUROD2 FOXB1 FOXG1 BHLHA15 BCOR FOXL2 SMYD3 PAX5 DMBX1 ZNF277 SP5 FOXI3 ETV5 CEBPA SPIB WBP2 RPS3 MMP12 RUNX3 HSPD1 NPAS2 ZNF431 TNF SCRT2 TSPYL2 |
| Up regulated | 7.45E-10 | 234 | Transcription regulatory region sequence-specific DNA binding | TFAP2D TBPL1 ZIC2 PRDM1 PRDM6 MEF2A TCF7 TFAP2C LHX5 XBP1 SIX4 BRF2 HOXA5 LHX2 LHX6 SETX PITX3 NKX3-2 CUX2 BACH2 TBP CDX1 BCL6 HES1 KDM3A DLX2 NFE2L2 SFPQ MEF2D TFAP2E PLAGL1 NR4A3 NFYB BHLHE41 NR4A1 ESX1 BATF3 FOSB NR1D1 PRDM7 ATF4 JUND GATA5 PRDM12 NFATC1 RARA PPARG KDM6B HEY2 EGR4 GATA4 GTF2B ASCL1 GATA6 LHX9 OSR1 DLX1 KDM6A ZNF711 HHEX NR4A2 CIART MED8 ATF3 MSX1 NKX6-1 ZNF513 PITX2 HEY1 DRGX NKX3-1 BATF2 IRF2BP2 GBX2 ATF5 FOS SIX2 JUNB NHLH1 JMJD1C ZNF131 DDIT3 ISX FOSL1 JUN HES7 GATA2 ARID3B EGR3 PCGF5 DDN MAFA NKX2-5 MAFF PURA NKRF RFX8 MAFG MAFK MAFB ASCL5 LHX1 SNAI2 SARS FOXC1 MXD1 YBX1 KLF6 RORA ENO1 TXK RRN3 ESR1 CREM ELF4 KLF5 CRX GLI3 AEBP1 HDAC5 GLI1 ELK3 HAND1 BCL11A EGR1 TBX2 TWIST1 EGR2 TOX2 SNAI1 SOX4 LRRFIP1 SOX9 PAX8 FOXA2 GZF1 FOXJ1 THAP1 H3F3B ARNTL MEIS2 GRHL1 MYCN ETS1 TBX3 LMO2 SKIL KLF4 MYC IRF4 GCM1 HMGA1 IRF8 EAF2 NOTCH1 ARID5B KLF10 ETS2 IER2 ZNF281 H3F3A KLF15 H2AFZ CREBRF NFIL3 E2F7 ZNF143 SP2 E2F6 CNBP CEBPB NPAS4 ETV4 HOXD8 FOXC2 TGIF1 ZBTB2 PRKN IRF7 FOXD3 ZKSCAN3 ZNF350 SCX SCRT1 SOX8 CRY1 POU2F2 YBX3 TFE3 HOXA9 EBF4 HNF4A EBF3 KCNIP3 GADD45A SATB2 RUNX2 BCL11B TBR1 DMRT1 LMO4 TBX19 MEIS1 ZEB1 CEBPG CXXC1 BACH1 IKZF3 FOXQ1 PKNOX2 PBX3 HOXB9 NEUROD2 OVOL1 BHLHA15 BCOR FOXL2 SMYD3 PAX5 DMBX1 ZNF277 SP5 CEBPA FOXD1 WBP2 RPS3 ETV5 SPIB RUNX3 AHR NPAS2 ZNF431 TNF SCRT2 |
| Up regulated | 7.45E-10 | 241 | Structural molecule activity | RPS20 RPS5 RPS19 RPS27A RPS3 FAU RPS29 RPL26L1 COL9A2 RPL31 TUBE1 MYBPC2 RPL6 RPLP0 RPL3 TUBB1 MYOM1 PLLP SGTA RPS16 RPL19 COL1A1 MRPL27 RPS13 RPS12 RPL24 RPS15 RPL21 RPL23 BFSP1 TUBA4A RPL36 RPL27 DAP3 ANXA1 MRPL47 TUBB2A TUBB2B RPLP1 RSL24D1 COL2A1 RPL3L RPS11 RPL13A MYOM3 RPL11 RPS8 RPL10 RPL7 SEPT14 RPL30 POM121L2 RPL8 RPL29 LCE3D SPRR3 SPRR2D MRPS18C ARPC2 RPL9 RPS14 RPL27A SPRR1B CMTM8 RPS9 COL24A1 RPS21 MAL MRPL52 RPL38 RPL4 TUBB6 RPS27 IGSF22 RPL35A MRPL54 NCMAP RPS27L PSMD13 RPS23 MAGI2 MYBPC1 COL27A1 MRPL21 COL13A1 RPL12 MBP RPS4X PRR9 COL11A2 COL15A1 COL28A1 RPS28 SPRR2A MRPS6 TUBB3 ACTG1 MAPK8IP2 VIM MYL6 LAMTOR3 ARPC3 CSTA IVL RPLP2 KRT10 HSPB6 KRT33A ELN RPL18 SLC9A3R2 RPS18 CRYBB1 NEFH LAMA1 SRPX2 HOMER2 NEFM LIM2 RPL18A PCOLCE CLDN15 AEBP1 RPL28 KRT37 RPL34 CRYAB TECTA VWF KRT18 MGP MRPL18 RPL22 MRPS15 NID1 MFAP2 MAPK8IP1 CLTA KRT17 GFAP KRT34 KRT33B EMILIN2 NES PRPH KRT7 RPL35 RPS6 RPS24 CILP COL6A1 COL6A2 GRIP2 RPL32 MUC4 RPS3A RPL37 POMZP3 SNTG1 HMCN2 INA DLG2 PDLIM3 GRIP1 SEC13 CLDN14 MATN1 SYNC CRYGC COL6A3 CRYBA2 FBLN2 RPL22L1 RPL39L RPL36AL RPL13 RGS14 MUC17 KRT8 KRT78 KRT86 KRT9 MRPL36 KRT20 RPS7 TLN2 MMRN2 SPTBN2 CLTB RPS17 CLDN5 KRT6B CLDN24 KRT3 KRT16 KRT14 ZP3 PRELP RPL14 CLDN4 CRYBA4 LAMA2 LAMB3 RPL37A RPL10A RPL39 KRT6A KRT81 UBA52 TUBA4B CRYBB2 SPON1 NEXN TCAP MRPS24 SH3RF1 ANK1 MAP2 JAG1 MYL2 MAP1B MRPS36 TUFT1 MPZ DEDD2 MAP1A MYL1 SPAG4 ACTN2 EPB41L4B TFPI2 APOE HMGA1 ACTA1 BICD1 TPM4 SMTN OPTC TPM2 RACK1 ACTN3 |
| Up regulated | 3.08E-09 | 258 | Transcription regulatory region DNA binding | TFAP2D TBPL1 ZIC2 PRDM1 PRDM6 MEF2A TCF7 TFAP2C LHX5 XBP1 SIX4 TAF7L ZNF174 GABPB1 BRF2 HOXA5 LHX2 LHX6 SETX PITX3 NKX3-2 CUX2 BACH2 TBP CDX1 BCL6 HES1 KDM3A DLX2 NFE2L2 SFPQ MEF2D TFAP2E PLAGL1 NR4A3 NFYB BHLHE41 NR4A1 ESX1 BATF3 FOSB NR1D1 PRDM7 ATF4 JUND GATA5 PRDM12 NFATC1 RARA PPARG KDM6B HEY2 EGR4 GATA4 GTF2B ASCL1 GATA6 LHX9 OSR1 DLX1 KDM6A ZNF711 ARID5B HHEX NR4A2 CIART MED8 IKZF3 GFI1 ATF3 MSX1 NKX6-1 ZNF513 PITX2 HEY1 DRGX NKX3-1 BATF2 IRF2BP2 GBX2 ATF5 FOS SIX2 JUNB NHLH1 JMJD1C ZNF131 DDIT3 ISX FOSL1 BASP1 JUN TAF7 HES7 GATA2 ARID3B EGR3 PCGF5 DDN MAFA NKX2-5 MAFF PURA NKRF RFX8 ARID5A MAFG MAFK MAFB ASCL5 ZNF350 LHX1 SNAI2 SARS FOXC1 MXD1 YBX1 KLF6 RORA TBX21 ENO1 TXK RRN3 WNT11 ESR1 CREM HNF4A SMAD7 TBL1X ELF4 KLF5 CRX AHR GLI3 AEBP1 HDAC5 GLI1 ELK3 HAND1 BCL11A EGR1 TBX2 TWIST1 EGR2 TOX2 SNAI1 SOX4 LRRFIP1 WNT1 SOX9 PAX8 FOXA2 GZF1 CITED1 PRDX5 FOXJ1 THAP1 H3F3B ARNTL MEIS2 GRHL1 MYCN ETS1 TBX3 LMO2 SKIL KLF4 MYC IRF4 GCM1 HMGA1 IRF8 EAF2 NOTCH1 KLF10 ETS2 IER2 ZNF281 H3F3A TIPARP KLF15 H2AFZ CREBRF NFIL3 E2F7 ZNF143 SP2 E2F6 CNBP KLF11 CEBPB NPAS4 ETV4 HOXD8 FOXC2 TGIF1 NPM1 ZBTB2 PRKN IRF7 FOXD3 ZKSCAN3 LONP1 TNF ETV5 CEBPA SCX SCRT1 SOX8 CRY1 POU2F2 YBX3 TFE3 HOXA9 EBF4 EBF3 KCNIP3 GADD45A SATB2 RUNX2 BCL11B ELL3 TBR1 DMRT1 CBX4 LMO4 TBX19 MEIS1 ZEB1 CEBPG CXXC1 BACH1 FOXQ1 SOX17 PKNOX2 PBX3 HOXB9 NEUROD2 OVOL1 BHLHA15 BCOR FOXL2 SMYD3 PAX5 DMBX1 ZNF277 SP5 FOXD1 WBP2 RPS3 FOXA3 MMP12 SPIB RUNX3 NPAS2 SOX7 ZNF431 SCRT2 |
| Up regulated | 3.08E-09 | 241 | Sequence-specific double-stranded DNA binding | TFAP2D TBPL1 ZIC2 PRDM1 PRDM6 MEF2A TCF7 TFAP2C LHX5 ORC6 CDC6 XBP1 SIX4 BRF2 HOXA5 LHX2 LHX6 SETX PITX3 NKX3-2 CUX2 BACH2 TBP CDX1 BCL6 HES1 KDM3A DLX2 NFE2L2 SFPQ MEF2D TFAP2E PLAGL1 NR4A3 NFYB BHLHE41 NR4A1 ESX1 BATF3 FOSB NR1D1 PRDM7 ATF4 JUND GATA5 PRDM12 NFATC1 RARA PPARG KDM6B HEY2 EGR4 GATA4 GTF2B ASCL1 GATA6 LHX9 OSR1 DLX1 KDM6A ZNF711 HHEX NR4A2 CIART MED8 ATF3 MSX1 NKX6-1 ZNF513 PITX2 HEY1 DRGX NKX3-1 BATF2 IRF2BP2 GBX2 ATF5 FOS SIX2 JUNB NHLH1 JMJD1C ZNF131 DDIT3 ISX FOSL1 JUN HES7 GATA2 ARID3B EGR3 PCGF5 DDN MAFA NKX2-5 MAFF PURA NKRF RFX8 MAFG MAFK MAFB ASCL5 LHX1 SNAI2 SARS FOXC1 MXD1 YBX1 KLF6 RORA ENO1 TXK RRN3 ESR1 CREM ELF4 KLF5 CRX AHR GLI3 AEBP1 HDAC5 GLI1 ELK3 HAND1 BCL11A EGR1 TBX2 TWIST1 EGR2 TOX2 SNAI1 SOX4 LRRFIP1 SOX9 PAX8 FOXA2 GZF1 FOXJ1 THAP1 H3F3B ARNTL MEIS2 GRHL1 MYCN ETS1 TBX3 LMO2 SKIL KLF4 MYC IRF4 GCM1 HMGA1 IRF8 EAF2 NOTCH1 ARID5B KLF10 ETS2 IER2 ZNF281 H3F3A KLF15 H2AFZ CREBRF NFIL3 E2F7 ZNF143 SP2 E2F6 CNBP CEBPB NPAS4 ETV4 HOXD8 FOXC2 TGIF1 BEND3 ZBTB2 PRKN IRF7 FOXD3 ZKSCAN3 CRIP1 ZNF350 SCX SCRT1 SOX8 CRY1 POU2F2 YBX3 TFE3 HOXA9 EBF4 HNF4A EBF3 SUB1 KCNIP3 GADD45A SATB2 RUNX2 BCL11B TBR1 DMRT1 LMO4 TBX19 MEIS1 ZEB1 CEBPG CXXC1 BACH1 IKZF3 FOXQ1 PKNOX2 PBX3 HOXB9 NEUROD2 OVOL1 BHLHA15 BCOR FOXL2 SMYD3 PAX5 DMBX1 ZNF277 SP5 CEBPA FOXD1 WBP2 RPS3 ETV5 SPIB RUNX3 HSPD1 NPAS2 ZNF431 TNF SCRT2 TSPYL2 |
